# On the Road towards Small-Molecule Programmed Cell Death 1 Ligand 1 Positron Emission Tomography Tracers: A Ligand-Based Drug Design Approach

**DOI:** 10.3390/ph16071051

**Published:** 2023-07-24

**Authors:** Karsten Bamminger, Verena Pichler, Chrysoula Vraka, Tina Nehring, Katharina Pallitsch, Barbara Lieder, Marcus Hacker, Wolfgang Wadsak

**Affiliations:** 1 CBmed GmbH—Center for Biomarker Research in Medicine, 8010 Graz, Austria; 2 Department of Biomedical Imaging and Image-Guided Therapy, Division of Nuclear Medicine, Medical University of Vienna, 1090 Vienna, Austria; 3 Department of Pharmaceutical Sciences, Division of Pharmaceutical Chemistry, University of Vienna, 1090 Vienna, Austria; 4 Institute of Organic Chemistry, University of Vienna, 1090 Vienna, Austria; 5 Department of Physiological Chemistry, University of Vienna, 1090 Vienna, Austria

**Keywords:** programmed cell death 1 ligand 1, positron emission tomography, carbon-11, radiotracer design

## Abstract

PD-1/PD-L1 immune checkpoint blockade for cancer therapy showed promising results in clinical studies. Further endeavors are required to enhance patient stratification, as, at present, only a small portion of patients with PD-L1-positive tumors (as determined by PD-L1 targeted immunohistochemistry; IHC) benefit from anti-PD-1/PD-L1 immunotherapy. This can be explained by the heterogeneity of tumor lesions and the intrinsic limitation of multiple biopsies. Consequently, non-invasive in vivo quantification of PD-L1 on tumors and metastases throughout the entire body using positron emission tomography (PET) imaging holds the potential to augment patient stratification. Within the scope of this work, six new small molecules were synthesized by following a ligand-based drug design approach supported by computational docking utilizing lead structures based on the (2-methyl-[1,1′-biphenyl]-3-yl)methanol scaffold and evaluated in vitro for potential future use as PD-L1 PET tracers. The results demonstrated binding affinities in the nanomolar to micromolar range for lead structures and newly prepared molecules, respectively. Carbon-11 labeling was successfully and selectively established and optimized with very good radiochemical conversions of up to 57%. The obtained insights into the significance of polar intermolecular interactions, along with the successful radiosyntheses, could contribute substantially to the future development of small-molecule PD-L1 PET tracers.

## 1. Introduction

Immune checkpoint inhibitors and their application in immunotherapy have led to tremendous progress in oncological practice [1]. The PD-1/PD-L1 immune checkpoint works as an inhibitory regulatory mechanism to prevent excessive immune reactions and autoimmunity for self-tolerance. Cancer cells can exploit this mechanism for immune evasion by overexpressing programmed cell death 1 ligand 1 (PD-L1), thereby promoting cancer development and progression through negatively regulating T-cell-mediated immune responses and suppressing migration, proliferation and effector function of T cells, eventually inducing T cell exhaustion, a dysfunctional state of T cells lacking effector function accompanied by an increased expression of inhibitory receptors [2]. Several immune checkpoint inhibitors (ICIs) have been developed so far to block this ligand-receptor interaction, thereby reactivating the host immune system to fight cancer. Monoclonal antibodies targeting PD-1, e.g., pembrolizumab, or PD-L1, e.g., atezolizumab, have come into the spotlight, leading to marketing authorization, and many more are in clinical trials [3].

Positron emission tomography (PET) with PD-L1-targeting ligands could overcome current limitations associated with PD-L1 quantification of primary and metastatic tumors by immunohistochemistry (IHC). Several studies have reported the successful application of radiolabeled antibodies for PD-L1 PET imaging. For example, a study by Hettich et al. [4] utilized a PD-L1-specific antibody labeled with zirconium-89 to visualize PD-L1 expression in mouse models of lung cancer. The PET images accurately reflected the heterogeneity of PD-L1 expression in the tumors, demonstrating the potential of this technique for patient stratification and monitoring treatment response. Recently, Bensch et al. [5] showed that pre-treatment PD-L1 PET quantification using the zirconium-89 labeled anti-PD-L1 antibody atezolizumab ([^89^Zr]Zr-atezolizumab) correlated better with clinical response than IHC or RNA-sequencing. [^89^Zr]Zr-atezolizumab uptake was a strong predictor of response to atezolizumab treatment, including PFS and OS.

Despite its potential, PET imaging of PD-L1 using radiolabeled antibodies has some limitations. One major drawback is the relatively slow clearance of the radiotracer from non-target tissues, which can lead to high background signal and reduced imaging contrast. Strategies to enhance tumor-to-background ratio include the use of smaller antibody fragments or peptide-based PD-L1-targeting ligands, which clear more rapidly from non-target tissues, like minibodies [6], nanobodies [7], monobodies (adnectines) [8] and affibodies [9] radiolabeled with, e.g., copper-64, gallium-68, zirconium-89 or fluorine-18 for PD-L1 PET imaging [10]. Further research and clinical validation are needed to optimize imaging properties, evaluate their safety profile and establish their clinical utility in PD-L1 PET imaging. In addition, endeavors have been undertaken to advance the development of tracers based on peptides and small molecules, although the body of published literature pertaining to the latter is significantly constrained. A detailed review on the development of radiotracers for the PD-1/PD-L1 axis has been published recently by Krutzek et al. [10].

**Figure 1:**
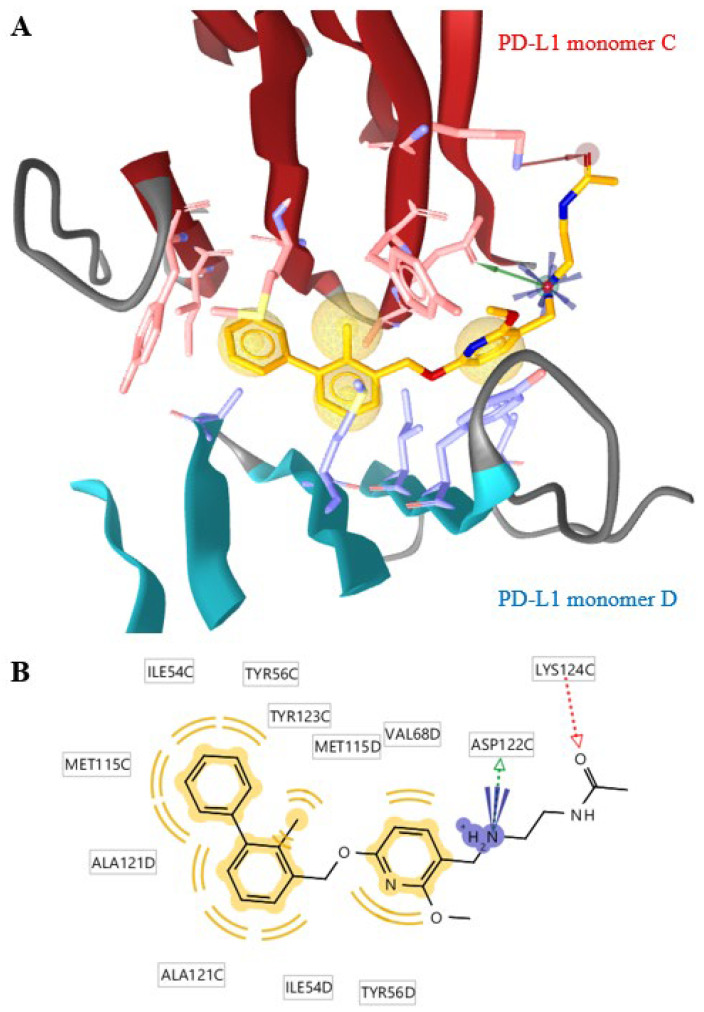
The binding mechanism of lead structure **2** determined by crystallography (PDB: 5J89) using the LigandScout 4.4 software. (**A**) A cut-out of the crystal structure of one inhibitor molecule (orange) and two PD-L1 proteins (dark red and teal) are shown (mixed ribbon/surface representation). The pharmacophore is represented by hydrophobic features (yellow spheres), a positive ionizable area (blue spikes), a hydrogen bond donor (green vector) and a hydrogen bond acceptor (red vector). The interaction of **2** is dominated by hydrophobic interactions with both PD-L1 monomers C (red sticks) and monomer D (blue sticks). (**B**) The distant phenyl ring of the 2-methylbiphenyl moiety is stabilized by T-stacking interaction with the phenyl ring of the tyrosine sidechain (TYR56C) and π–alkyl interactions of methionine (MET115C) and alanine (ALA121D) sidechains. The methylphenyl ring interacts with alanine (ALA121C) and methionine (MET115D), and the methyl group of this ring fits into a pocket formed by monomer C. The two rings are twisted by about 45°. The interactions of the methoxypyridyl ring include π–π stacking with tyrosine (TYR56D) and other nonpolar interactions with monomer D. The external N-(2-amino-ethyl)acetamide unit only interacts with monomer C and seems to be more accessible for chemical modifications than the rigid inner ring system. The grid box was automatically determined by LigandScout^®^ and comprises around 30 × 30 × 30 Å.

The objective of this study was to employ a ligand-based drug design approach to develop PD-L1 selective PET tracers based on two available lead structures. The design process was guided by molecular docking to facilitate the identification of suitable modifications for optimizing the selectivity of the potential PET tracers towards PD-L1. Lead structures demonstrated low IC_50_ values of 6–100 nM and 18 nM, respectively, in a homogeneous time-resolved fluorescence (HTRF) assay [11]. X-ray crystallography and size-exclusion chromatography revealed that these compounds induce dimerization of PD-L1 by forming a hydrophobic pocket (Figure 1), and dimerization remained stable upon dissociation of ligands [12,13]. The nonpolar interactions are the driving force for binding to and inducing dimerization of PD-L1, while polar contributions are unfavorable according to computational methods [13,14]. Hence, the flexible, polar residues at the exit of this pocket could be accessible for minor modifications without tremendous adverse impact on binding affinity. Therefore, the lead structures were chemically modified to make them susceptible for carbon-11 or fluorine-18 labeling, i.e., heteroatom methylation or fluoroethylation. Moreover, compounds were evaluated in vitro regarding stability, lipophilicity and *h*PD-L1 binding affinity. Eventually, radiolabeling was established, and the reaction conditions were optimized for radiochemical conversion, and the radioligand exhibiting the highest binding affinity was evaluated for metabolic stability.

## 2. Results and Discussion

Starting from available lead structures **1** and **2**, ligand docking experiments were performed against human PD-L1 (PDB: 5J89) using the LigandScout software. For validation, docking software is considered reliable when a generated pose is very similar to the original pose in the protein-ligand crystal structure reaching a root mean square deviation (RMSD) of <1.5–2 Å between the docked and original pose [15]. In our case, a RMSD of 0 Å was achieved by redocking the original ligand, highlighting its reliability. The crystal structure of *h*PD-L1 shows the dimeric organization of the protein units, with the lead structure(s) bound in a sandwich-like blot between two dimers with the terminal carboxylic acid or amide functionality reaching out of the hydrophobic binding pocket [12]. Based on minor interaction of the eastern part of both lead structures with the binding cleft, we started our chemical modification at the structural point with minimal target interaction. Based on compound **1**, the terminal carboxylate group was equipped with a methyl (**1a**) or a fluoroethyl group (**1b**) to allow the envisioned radiolabeling with either carbon-11 or fluorine-18. Methylation of compound **2** at different positions led to the generation of a tertiary amine (**2a**), tertiary amide (**2b**) as well as a double methylated compound (**2c**), while fluoroethylation led to the corresponding fluoroethylated derivative (**2d**). Comparison of the pharmacophore revealed the loss of the interaction with ASP122C, by leaving the main pharmacophoric interactions unchanged (Supplementary Figures S2–S8). The impact of the structural changes on the docking parameters, i.e., binding affinity score and affinity, were marginal. Overall, pharmacophore assessment predicted minor effects of methylation (compound **1a**, **2a**, **2b** and **2c**) and fluoroethylation (**1b**, **2d**) on binding affinity (Table 1).

**Scheme 1:**
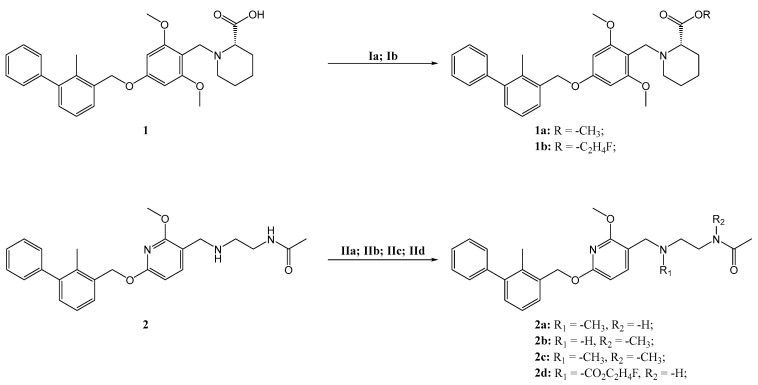
Synthesis scheme and reaction conditions: (**Ia**) MeI (3.0 eq.), Cs_2_CO_3_ (1.5 eq.), DMSO, 60 °C, 24 h, 5%; (**Ib**) 2-fluoroethyl tosylate (4.0 eq.), Cs_2_CO_3_ (3.2 eq.), DMSO, 100 °C, 10 min, 61%; (**IIa**) MeI (3.0 eq.), MeCN, 50 °C, 30 min, 41%; (**IIb**) Boc_2_O (1.2 eq.), THF, rt, 24 h, 85%; MeI (6.0 eq.), TBAH (5.0 eq.), MeCN, 100 °C, 10 min, 9%; conc. HCl, MeCN, rt, 24 h, 68%; (**IIc**) MeI (3.0 eq.), TBAH (1.2 eq.), 100 °C, 20 min, 8%; (**IId**) 2-fluoroethyl tosylate (4.0 eq.), Cs_2_CO_3_ (3.2 eq.), DMSO, 100 °C, 10 min, 75%.

Selected small molecules derived from the lead structures were subsequently synthesized by O- and N-methylation and fluoroethylation (Scheme 1) to obtain the respective non-radioactive reference compounds for biological assessment and to set up radiosynthesis and quality control. Briefly, methyl ester **1a** was obtained by Steglich esterification, conversion into an acyl imidazole and nucleophilic substitution with methyl iodide, although only the latter resulted in a sufficiently pure product (Supplementary Table S1). Fluoroethylated compound **1b** was readily obtained using 2-fluoroethyl *p*-toluenesulfonate under basic conditions. Synthesis of **2a**, **2b** and **2c** was straightforward using methyl iodide. No additional base was required for the synthesis of monomethylated **2a**, while an additional base was needed for the formation of dimethylated compound **2c**. Substance **2b** was selectively produced by Boc protection of the secondary amine prior to methylation of the amide, followed by acidic Boc deprotection. Fluoroethylation in the presence of cesium carbonate resulted in **2d** in good yield. In general, the conversions into methylated products were incomplete, as determined by analytical HPLC measurements, although methyl iodide was used in excess. As a result, product **1a**, **2b** and **2c** were achieved in low overall yields of 5–8%, and **2a** was isolated with a moderate yield of 41% (Scheme 1). Interestingly, when **2** was used as a substrate for fluoroethylation, no conversion occurred when *N,N*-diisopropylethylamine or potassium *tert*-butoxide was used as a base (Supplementary Table S1). The use of cesium carbonate led to carbamate formation of the amine functionality [16,17], which then functioned as a nucleophile for fluoroethylation, resulting in product **2d**. Dealkylation attempts of the lead structures’ aryl ethers for the synthesis of radiolabeling precursors using Lewis acids [18] (AlCl_3_, BBr_3_) did not achieve the desired outcome.

Because no in-solution stability data were available, all lead structures and compounds were tested for their stability in DMSO/HEPES buffer over an extended period of time (20 days) before further in vitro analysis. In general, compounds were more stable when stored at 4–8 °C compared to room temperature. Compounds **2**, **2a**, **2c** and **2d** were highly stable in solution with marginal decomposition of less than 2% of the parent compound, while **1**, **1a**, **1b** and **2b** remained intact to over 85% within the 20-day time period (Supplementary Figures S9–S16).

**Figure 2:**
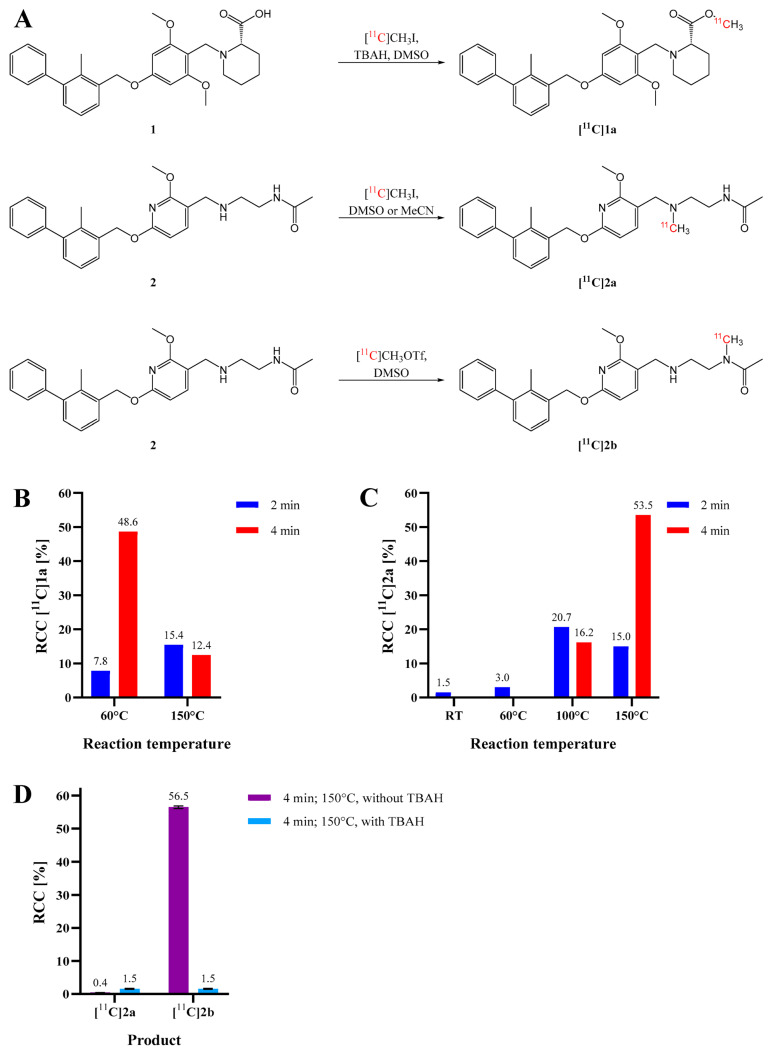
Scheme of radiosynthesis (**A**) and radiochemical conversion (RCC) of (**B**) **[^11^C]1a** and (**C**) **[^11^C]2a** at various reaction conditions using [^11^C]CH_3_I and 2 mg/mL precursor without a base. (**D**) RCC of **[^11^C]2a** and **[^11^C]2b** at different reaction conditions using [^11^C]CH_3_OTf with or without aqueous TBAH as a base. Standard deviations are represented as error bars (n = 2).

Further efforts were made to establish radiolabeling strategies with respect to future structurally related compounds. Small-scale carbon-11 radiosyntheses of small molecules **[^11^C]1a**, **[^11^C]2a** and **[^11^C]2b** were performed and peaked in a radiochemical conversion (RCC) of 49%, 54% and 57%, respectively, as determined by radio-HPLC (Figure 2A).

**Figure 3:**
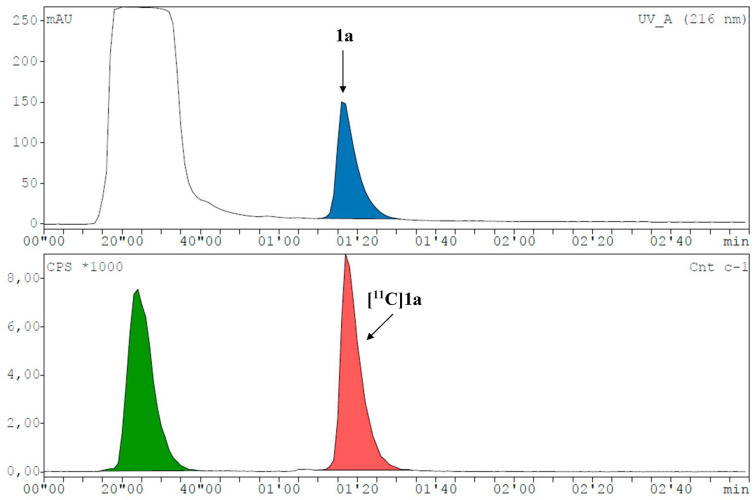
Representative radio-HPLC chromatogram (UV channel top, radioactivity channel bottom) for the synthesis of **[^11^C]1a** with a RCC of 48.6% (corrected for decay during HPLC measurement). Precursor **1** eluted at the dead time of the system together with DMSO.

**Figure 4:**
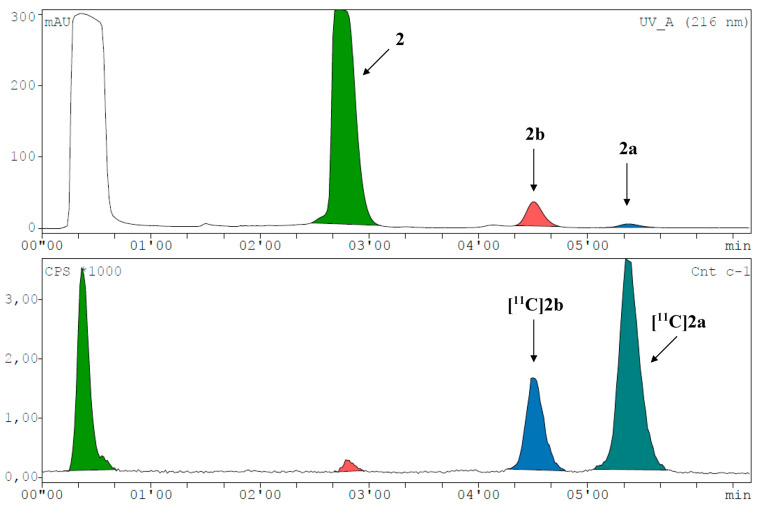
Representative radio-HPLC chromatogram (UV channel top, radioactivity channel bottom) spiked with reference compounds for the synthesis of **[^11^C]2a** and **[^11^C]2b** with RCCs of 53.5% and 19.6% (corrected for decay during HPLC measurement), respectively.

**Figure 5:**
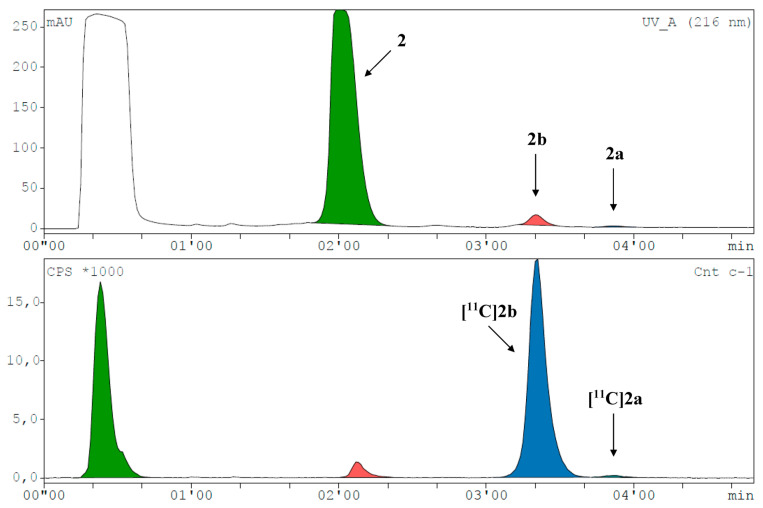
Representative radio-HPLC chromatogram (UV channel top, radioactivity channel bottom) spiked with reference compounds for the selective synthesis of **[^11^C]2b** with a RCC of 56.5% (corrected for decay during HPLC measurement).

**[^11^C]1a** was preferably produced using [^11^C]CH_3_I in MeCN at 60 °C for 4 min (Figure 2B and Figure 3). As **[^11^C]2a** and **[^11^C]2b** originate from the same precursor molecule, the selective production of these constitutional isomers was a special challenge. [^11^C]CH_3_I as methylating agent yielded preferably the tertiary amine (54%), and the tertiary amide was identified as the major by-product with around 20% at 150 °C and a reaction time of 4 min (Figure 2C and Figure 4, Supplementary Figure S18E). However, **[^11^C]2b** was preferentially formed by applying the more reactive [^11^C]CH_3_OTf synthon (Figure 2D and Figure 5) with only marginal amounts of the by-product **[^11^C]2a**. The addition of a base, like TBAH, completely quenched the reaction. However, by-product formation was mainly a matter of an increase in radiochemical conversion rather than purity, as both products were clearly separated by HPLC. In summary, we were able to selectively radiolabel both constitutional isomers in high RCC.

**Figure 6:**
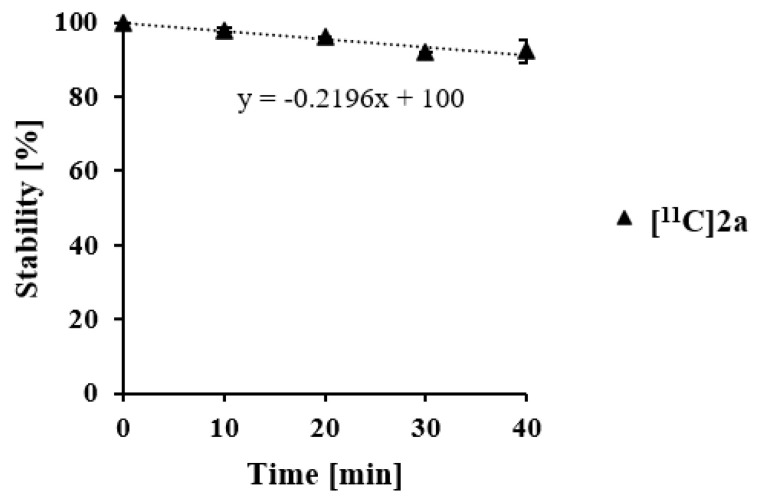
Metabolic stability of **[^11^C]2a** in human liver microsomes over time (n = 2).

**[^11^C]2a** was further evaluated for metabolic stability using human liver microsomes. **[^11^C]2a** remained metabolically stable with ~90% intact tracer after incubation for 40 min (Figure 6).

Physicochemical parameters were calculated and measured for cross-validation and to establish structure–activity relationships. Measured ${µ\mathrm{HPLC}\;\log P}_{\mathrm{OW}}^{\mathrm{pH}\;7.4}$ values ranged from 3.16 to 5.02 (Table 2). Modification of **2** (µHPLC log*D* 3.88) resulted in increasing lipophilicity ranked in the following order: **2b** < **2a** < **2d** < **2c**. Methylation and fluoroethylation of the carboxyl group of **1** (µHPLC log*D* 3.16) culminated in the highest measured lipophilicity of 4.90 and 5.02 for **1a** and **1b**, respectively. The calculated physicochemical parameters predicted the increase in lipophilicity in dependency of the chemical modification (Table 2). However, calculated values deviated significantly from measured values by overestimating the lipophilic character of our compounds. Given their molecular weight, high lipophilicity (µHPLC log*D*) and low topological polar surface area (tPSA), these compounds should easily permeate cell membranes and could potentially penetrate the blood–brain barrier [19,20].

*h*PD-L1 binding affinities of the developed compounds were assessed by means of an HTRF binding assay including the antibody atezolizumab, the macrocyclic peptide PD-1/PD-L1 Inhibitor 3 and lead structures **1** and **2** as positive controls. Binding affinities (IC_50_) were found to be in the micro- to nanomolar range (101–9880 nM) for small molecules, while mean IC_50_ values of 4.07 nM and 113 nM were determined for reference compounds atezolizumab and commercially available PD-1/PD-L1 Inhibitor 3, respectively. We were not able to reproduce the high *h*PD-L1 affinity values for the lead structures as published by Bristol Myers Squibb [11] (Table 3 and Supplementary Figure S17). Similar findings were recently published for the macrocyclic peptide BMS-78 [21]. This discrepancy may result from different protein concentrations applied within different assays. Affinities of our lead structure **1** (IC_50_: 202 ± 27 nM) derivatives were in the micromolar range with **1b** (1440 ± 144 nM) exhibiting better binding affinity than **1a** (5760 ± 613 nM). Substance **2a** had the most promising IC_50_ of 430 ± 62 nM, with a 4.3-fold reduced affinity compared to lead structure **2** (101 ± 10 nM). The constitutional isomer **2b** showed similar *h*PD-L1 binding affinity to **2a** (IC_50_ of 524 ± 67 nM), whereas the dimethylated compound **2c** demonstrated lower affinity (IC_50_ of 1310 ± 185 nM). Introduction of a sterically demanding fluoroethyl carbamate moiety (compound **2d**) further decreased the binding affinity to 9880 ± 1390 nM. A clear trend of measured binding affinity depending on of the sterical demand of the introduced group(s) can be drawn based on our results. The measured affinity trend was inverse for calculated binding affinity scores.

Although the 2-methylbiphenyl core of the molecules was found to be the main pharmacophore responsible for binding to *h*PD-L1 [12], we further deduced that (i) there is a potential underestimation of the sterical hindrance within our computational docking model, (ii) the polar residues of the molecules and associated hydrogen bonds located at the exit of the binding pocket may also have a significant impact on binding affinity and that (iii) increasing lipophilicity leads to additional non-specific binding that impedes ligand binding during the HTRF assay.

We used Spearman’s rank correlation to investigate the direction and strength of our associated variables (binding affinity score, affinity and physicochemical parameters) and to statistically assess our interpretation. Non-significant weak correlations were found between calculated physicochemical parameters and measured HTRF IC_50_ values (Supplementary Table S2): clog*P* (ρ = 0.29, *p* = 0.49), tPSA (ρ = 0.16, *p* = 0.71), clog*D* (ρ = 0.31, *p* = 0.46) and ${µ\mathrm{HPLC}\;\log P}_{\mathrm{OW}}^{\mathrm{pH}\;7.4}$ (ρ = 0.31, *p* = 0.46). A moderate negative correlation was found for binding affinity score (ρ = −0.52, *p* = 0.18) and affinity (ρ = −0.64, *p* = 0.09). In summary, the applied in silico methods were not successful in predicting *h*PD-L1 binding affinity. The unaccounted flexibility of the PD-L1 protein structure is a general limitation of static docking experiments, among others.

Moreover, a set of efficiency indices was computed to assess the impact of chemical modifications as a means to optimize the selection of fragments/leads during the drug development process. These indices encompassed ligand efficiency (LE), binding efficiency index (BEI), ligand lipophilicity efficiency (LLE) and ligand-efficiency-dependent lipophilicity efficiency (LELP). These parameters were evaluated with respect to molecular size (non-hydrogen atom count), molecular weight, lipophilicity and a combination of molecular size and lipophilicity, respectively [22]. Generally, higher values of these indices indicate improved efficiency. However, it is important to note that the optimal ranges for LLE and LELP may differ based on the specific target and therapeutic field. The results obtained from our analysis (as presented in Table 4) indicate that the reduction in binding affinity was not compensated by the concurrent increase in molecular size (LE) or molecular weight (BEI). Additionally, the elevation in lipophilicity pushed the compounds into a domain associated with potentially unfavorable pharmacokinetic characteristics (LEE, LELP).

## 3. Experiments

### 3.1. General Information

All solvents and chemicals were obtained from commercial suppliers and used without further purification unless otherwise stated.

### 3.2. Methods

#### 3.2.1. High-Performance Liquid Chromatography

For high-performance liquid chromatography (HPLC) measurements after non-radioactive syntheses (setups 1–7), an Agilent 1200 series LC system (Agilent Technologies Inc., Santa Clara, CA, USA) paired with an Agilent 1100 series autosampler, an XBridge C18 HPLC column, (5 µm, 4.6 × 150 mm; Waters Corporation, Eschborn, Germany) and GINA Star Software (Raytest Isotopenmessgeräte GmbH, Straubenhardt, Germany) for data acquisition were used. Solvent “A” consisted of 90% *v/v* acetonitrile (MeCN) (Merck KGaA, Darmstadt, Germany) plus 10% *v/v* Milli-Q H_2_O (Merck KGaA, Darmstadt, Germany) and solvent “B” of a 10 mM sodium phosphate (Merck KGaA, Darmstadt, Germany) buffer adjusted to pH 7.4 with 1 mol/L NaOH (Merck KGaA, Darmstadt, Germany). The flow rate was set to 1.5 mL/min.

Setup 1: An isocratic mixture of 80% A: 20% B was used as mobile phase.

Setup 2: A mobile phase gradient of 50% A: 50% B to 80% A: 20% B within 10 min and a hold until the end of the run was used.

Setup 3: An isocratic mixture of 60% A: 40% B was used as mobile phase.

For semi-preparative purification, an Agilent 1200 series LC system was paired with a SUPELCOSIL™ ABZ+ HPLC column, 5 μm, 25 cm × 10 mm (Merck KGaA, Darmstadt, Germany). Solvent “A” consisted of 90% *v/v* MeCN plus 10% *v/v* Milli-Q H_2_O and solvent “B” of 10 mM sodium phosphate buffer adjusted to pH 7.4 with 1 mol/L NaOH. The flow rate was set to 5 mL/min.

Setup 4: A mobile phase gradient of 50% A: 50% B to 75% A: 25% B within 10 min and a hold until the end of the run was used.

Setup 5: An isocratic mobile phase of 75% A: 25% B was used.

Setup 6: A mobile phase gradient of 50% A: 50% B to 60% A: 40% B within 10 min and a hold until the end of the run was used.

Setup 7: An isocratic mobile phase of 60% A: 40% B was used.

For high-performance liquid chromatography measurements after radiosynthesis, an Agilent Technologies 1620 Infinity system was utilized with an Aqua^®^ C18, 5 µm, 125 Å, LC column 150 × 4.6 mm (Phenomenex Inc., Aschaffenburg, Germany) as stationary phase and GINA Star Software for data acquisition.

Setup 8: A mobile phase of 80% A: 20% B and a flow rate of 1.0 mL/min was used. “A” consisted of 90% *v/v* MeCN in Milli-Q H2O and “B” of 10 mM sodium phosphate buffer pH 7.4 with 1 mol/L NaOH. For biocide purposes, a spatula tip NaN3 (Merck KGaA, Darmstadt, Germany) was added to “B”.

Setup 9: An isocratic mobile phase of 50% A: 50% B and a flow rate of 1.0 mL/min was used. “A” consisted of 90% *v/v* MeCN in Milli-Q H2O and “B” of 50 mM ammonium dihydrogen phosphate (Honeywell International Inc., Charlotte, NC, USA) adjusted to pH 9.3 with 5 mol/L NaOH. For biocide purposes, a spatula tip’s worth of NaN3 was added to “B” and eventually filtered through a pleated filter (Cytiva, Marlborough, MA, USA).

Setup 10: For log*D* measurements, an Agilent 1200 series was paired with an Agilent 1100 autosampler and Agilent 1100 UV detector, an apHera™ column (10 × 6 mm, 5 μm; Merck KGaA, Darmstadt, Germany), GINA Star Software for data acquisition and a mobile phase gradient of 10% A and 90% B to 100% A within 9.4 min and back to starting conditions until minute 12. An equilibration time of 2 min before measurements was set. Solvent “A” consisted of methanol (Merck KGaA, Darmstadt, Germany) and solvent “B” of 10 mM sodium phosphate buffer pH 7.4. The flow rate was set to 1.5 mL/min.

Setup 11: For semi-preparative purification after radiosynthesis, the GE TRACERlab™ FX2 C synthesis module (General Electric Medical Systems, Uppsala, Sweden) was paired with a Sykam S1122 pump (Sykam, Eresing, Germany), a BlueShadow UV detector (KNAUER Wissenschaftliche Geräte GmbH, Berlin, Germany) and a SUPELCOSIL™ ABZ+ HPLC column, 5 μm, 25 cm × 10 mm. The solvent consisted of 55% MeCN and 45% 10 mM sodium phosphate buffer adjusted to pH 7.4 with 1 mol/L NaOH. The flow rate was set to 5 mL/min.

#### 3.2.2. Characterization

^1^H-NMR, ^13^C-NMR and ^19^F-NMR spectra were recorded in CDCl_3_ or DMSO-d_6_ (Merck KGaA, Darmstadt, Germany) on Bruker AV NEO 400, AV NEO 500 WB or AV III 600 spectrometers (Bruker, Mannheim, Germany). Spectra evaluation was performed using MestReNova 14.2 software (Mestrelab Research S.L., Santiago de Compostela, Spain). Full-scan HRMS spectra (*m*/*z* 50–1600) of the compounds dissolved in acetonitrile/methanol and 1% H2O were obtained by direct infusion measurements on a maXis ESI-Qq-TOF mass spectrometer (Bruker, Mannheim, Germany). The sum formulas of the detected ions were determined using Compass DataAnalysis 4.0 (Bruker, Mannheim, Mannheim, Germany) based on the mass accuracy (Δ*m*/*z* ≤ 5 ppm) and isotopic pattern matching (SmartFormula algorithm).

### 3.3. Ligand Docking Experiments

Compound structures were protonated to pH 7.4 using MarvinSketch 22.13 software. Ligand docking was then performed with LigandScout 4.4 software (Inte:Ligand GmbH, Vienna, Austria) using the AutoDock Vina 1.1 program and PDB code 5J89 (PD-L1 monomer C and D). Water and ethylene glycol molecules were removed prior to docking. Docking was performed in triplicates for more consistent results using the default settings (Exhaustiveness: 8; max. number of modes: 9; max. energy difference: 3).

### 3.4. Syntheses

#### 3.4.1. Synthesis of Methyl (*S*)-1-(2,6-Dimethoxy-4-((2-methyl-[1,1′-biphenyl]-3-yl)methoxy)benzyl)piperidine-2-carboxylate (**1a**)

In a Wheaton vial (Thermo Fisher Scientific Inc., Waltham, MA, USA), (*S*)-1-(2,6-dimethoxy-4-((2-methyl-[1,1′-biphenyl]-3-yl)methoxy)benzyl)piperidine-2-carboxylic acid (lead structure **1**) (15.0 mg, 31.5 μmol) (Selleck Chemicals Llc, Houston, TX, USA) was dissolved in 0.5 mL anhydrous DMSO (Merck KGaA, Darmstadt, Germany). Cesium carbonate (15.0 mg, 46.0 µmol) (Merck KGaA, Darmstadt, Germany) and methyl iodide (5.89 μL, 94.6 μmol) (Merck KGaA, Darmstadt, Germany) were added subsequently to this solution, which was stirred at 60 °C for 24 h. The resulting solution was diluted with 1 mL 10 mM sodium phosphate buffer pH 7.4 and 1 mL Milli-Q H_2_O. The resulting suspension was centrifuged for 4 min at 21,500× *g*. The supernatant was removed, and the precipitate was washed with Milli-Q H_2_O and centrifuged again. After removal of the supernatant, the precipitate was dissolved in dichloromethane (Merck KGaA, Darmstadt, Germany) and dried over MgSO_4_. Isolation of the product was performed by preparative TLC using pre-coated silica gel 40 F_254_ TLC plates (Merck KGaA, Darmstadt, Germany) and a 1:1 mixture of *n*-hexane (Merck KGaA, Darmstadt, Germany) and ethyl acetate (Honeywell International Inc., Charlotte, NC, USA). The spots were visualized using a UV lamp (Vilber Lourmat Deutschland GmbH, Eberhardzell, Germany) at 254 nm, and the corresponding product spot (R_f_ = 0.39) was scraped off the plate with a spatula. The silica gel particles containing the methylated product were mixed with a small amount (~2.5 mL) of ethyl acetate. The mixture was filtrated through a 0.22 µm Millex-GV PVDF filter. Remaining traces of ethyl acetate were removed at 40 °C facilitated by a slow flow of nitrogen gas, leaving the solid, colorless product (0.78 mg, 5% yield).

Purity: 95.39% as determined by HPLC setup 2, UV detector: 254 nm.

^1^H-NMR (600 MHz, CDCl_3_): δ 7.44–7.41 (m, 3H), 7.37–7.24 (m, 5H), 6.23 (s, 2H), 5.08 (s, 2H), 3.86–3.79 (m, 2H), 3.76 (s, 3H), 3.75 (s, 6H), 3.12–3.07 (m, 2H), 2.27 (s, 3H), 2.22–2.18 (m, 1H), 1.85–1.82 (m, 1H), 1.68–1.53 (m, 4H), 1.25–1.19 (m, 2H).

^13^C-NMR (151 MHz, CDCl_3_): δ 175.17, 160.39, 160.00, 143.17, 142.10, 135.29, 134.66, 130.45, 129.54, 128.50, 128.24, 127.02, 125.79, 106.64, 91.28, 69.39, 63.54, 55.74, 51.54, 50.47, 46.46, 30.62, 25.57, 23.08, 16.39.

ESI-MS ([M + H]^+^): *m/z* calculated ([C_30_H_35_NO_5_ + H]^+^) = 490.2588. Found = 490.2591.

#### 3.4.2. Synthesis of 2-Fluoroethyl (*S*)-1-(2,6-Dimethoxy-4-((2-methyl-[1,1′-biphenyl]-3-yl)methoxy)benzyl)piperidine-2-carboxylate (**1b**)

Lead structure **1** (10.0 mg, 21.0 µmol) was dissolved in 0.5 mL anhydrous DMSO. Cesium carbonate (22.0 mg, 67.5 µmol) and 2-fluoroethyl *p*-toluenesulfonate (14.3 µL, 84.1 µmol) (TCI Deutschland GmbH, Eschborn, Germany) were added subsequently, and the reaction mixture was stirred at 100 °C for 10 min. The reaction solution was diluted with 400 µL sodium phosphate buffer pH 7.4 (semi-prep. HPLC solvent “B”), and the product was purified by semi-preparative HPLC using HPLC setup 4. The organic solvent was removed under reduced pressure and elevated temperature (40 °C). The resulting suspension was extracted twice with ethyl acetate. The pooled organic phases were washed once with Milli-Q H_2_O and once with a sat. NaCl (brine) solution (Merck KGaA, Darmstadt, Germany) and dried over Na_2_SO_4_ (Merck KGaA, Darmstadt, Germany). The solvent was removed under reduced pressure and the residue dried over silica gel leaving the solid, colorless product (6.7 mg, 61% yield).

Purity: 99.98% as determined by HPLC setup 2, UV detector: 254 nm.

^1^H-NMR (600 MHz, CDCl_3_): δ 7.44–7.41 (m, 3H), 7.37–7.24 (m, 5H), 6.23 (s, 2H), 5.08 (s, 2H), 4.69–4.60 (m, *J_H,F_* = 47 Hz, 2H), 4.48–4.33 (m, 2H), 3.89–3.81 (td, *J* = 16 Hz, *J* = 14 Hz, 2H), 3.76 (s, 6H), 3.22 (dd, *J* = 4.4 Hz, *J* = 3.9 Hz, 1H), 3.12–3.10 (m, 1H), 2.27 (s, 3H), 2.25–2.23 (m, 1H), 1.87–1.85 (m, 1H), 1.73–1.70 (m, 1H), 1.61–1.57 (m, 2H), 1.29–1.25 (m, 2H).

^13^C-NMR (151 MHz, CDCl_3_): δ 174.52, 160.38, 159.89, 143.18, 142.10, 135.30, 134.67, 130.45, 129.54, 128.49, 128.24, 127.02, 125.79, 106.92, 91.36, 81.61 (d, *J* = 170 Hz), 69.39, 63.12 (d, *J* = 20 Hz), 62.92, 55.81, 55.79, 50.07, 46.36, 30.59, 25.61, 22.83, 16.39.

^19^F-{^1^H}-NMR (470 MHz, CDCl_3_): δ −224.24 (s, 1F).

ESI-MS ([M + H]^+^): *m*/*z* Calculated ([C_31_H_36_FNO_5_ + H]^+^) = 522.2650. Found = 522.2647

#### 3.4.3. Synthesis of *N*-(2-(((2-Methoxy-6-((2-methyl-[1,1′-biphenyl]-3-yl)methoxy)pyridin-3-yl)methyl)(methyl)amino)ethyl)acetamide (**2a**)

*N*-(2-(((2-methoxy-6-((2-methyl-[1,1′-biphenyl]-3-yl)methoxy)pyridin-3-yl)methyl)amino)ethyl)acetamide (lead structure **2**) (4.55 mg, 10.8 µmol) (Selleck Chemicals Llc, Houston, TX, USA) was dissolved in 1 mL anhydrous MeCN (Merck KGaA, Darmstadt, Germany) in a Wheaton vial, methyl iodide (2.0 µL, 32.8 µmol) was added and the reaction mixture was stirred at 50 °C for 30 min. The product was purified by prep. TLC with 3:2 ethyl acetate/methanol as mobile phase (R_f_ = 0.63) and extracted from the silica particles with methanol. After filtration through Celite^®^ and cotton wool (Merck KGaA, Darmstadt, Germany), methanol was removed at 50 °C in a N_2_ stream, yielding a colorless solid (1.93 mg, 41% yield).

Purity: 98.06% as determined by HPLC setup 3, UV detector: 254 nm.

^1^H-NMR (400 MHz, CDCl_3_): δ 7.46–7.30 (m, 7H), 7.25–7.23 (m, 2H), 6.37 (d, *J* = 7.9 Hz, 1H), 6.20 (br s, 1H), 5.42 (s, 2H), 3.97 (s, 3H), 3.42 (s, 2H), 3.37 (q, *J* = 5.6 Hz, 2H), 2.52 (t, *J* = 5.6 Hz, 2H), 2.28 (s, 3H), 2.20 (s, 3H), 1.97 (s, 3H).

ESI-MS ([M + H]^+^): *m/z* calculated ([C_26_H_31_N_3_O_3_ + H]^+^) = 434.2438. Found = 434.2435.

#### 3.4.4. Synthesis of *N*-(2-(((2-Methoxy-6-((2-methyl-[1,1′-biphenyl]-3-yl)methoxy)pyridin-3-yl)methyl)amino)ethyl)-*N*-methylacetamide (**2b**)

Lead structure **2** (10.0 mg, 23.8 µmol) was dissolved in 1 mL anhydrous THF (Merck KGaA, Darmstadt, Germany), di-*tert*-butyl dicarbonate (6.24 mg, 28.6 µmol) (Merck KGaA, Darmstadt, Germany) was added and the reaction was stirred at room temperature overnight. TLC with 4:1 ethyl acetate/methanol showed nearly quantitative conversion (R_f_ = 0.82). THF was evaporated, and ethyl acetate (1 mL) was added. The organic phase was subsequently washed with 0.5 mol/L aqueous HCl, sat. aqueous NaHCO_3_ (Merck KGaA, Darmstadt, Germany) solution and brine and dried over Na_2_SO_4_. Ethyl acetate was evaporated yielding a colorless, highly viscous liquid (10.6 mg, 85% yield). The Boc-protected intermediate (10.6 mg, 20.3 µmol) was dissolved in 1 mL anhydrous MeCN in a N_2_ atmosphere. Tetrabutylammonium hydroxide (TBAH; as 54–56% aq. Solution, 48.2 µL, 102 µmol) (Merck KGaA, Darmstadt, Germany) and methyl iodide (7.6 µL, 122 µmol) were added subsequently, and the reaction was stirred at 100 °C for 10 min. The product was purified by semi-prep. HPLC using HPLC setup 5. The organic solvent was removed under reduced pressure. The remaining aqueous solution was extracted twice with ethyl acetate, and the combined organic phases washed with Milli-Q H_2_O and brine and dried over Na_2_SO_4_. Ethyl acetate was removed under reduced pressure, yielding a colorless, highly viscous liquid (1.01 mg, 9% yield). Concentrated aqueous HCl (1.6 µL, 190 µmol) (Merck KGaA, Darmstadt, Germany) was added to the methylated and Boc-protected intermediate (1.01 mg, 1.90 µmol) dissolved in 1 mL anhydrous MeCN and stirred in an inert atmosphere at room temperature overnight. TLC with 4:1 ethyl acetate/methanol showed successful conversion (R_f_ = 0.10). The product was purified by semi-prep. HPLC setup 4. The organic solvent was removed under reduced pressure, and the resulting suspension was extracted twice with ethyl acetate. The pooled organic phases were washed with Milli-Q H_2_O and dried over Na_2_SO_4_. The organic solvent was removed under reduced pressure, yielding a colorless solid (0.56 mg, 68% yield).

Purity: 96.02% as determined by HPLC setup 3, UV detector: 216 nm.

^1^H-NMR (600 MHz, CDCl_3_): δ 7.45–7.40 (m, 4H), 7.36–7.30 (m, 3H), 7.24–7.21 (m, 2H), 6.35 and 6.33 (d*, *J* = 7.9 Hz, 1H), 5.42 and 5.41 (s*, 2H), 3.97 and 3.96 (s*, 3H), 3.70 and 3.69 (s*, 2H), 3.50 and 3.41 (t*, *J* = 6.7 Hz, 2H), 3.01 and 2.91 (s, 3H), 2.77 and 2.75 (t*, *J* = 6.7 Hz, 2H), 2.27 (s*, 3H), 2.12 and 2.08 (s*, 3H). Note: Certain peaks are split due to the presence of amide rotamers and are denoted with an asterisk.

ESI-MS ([M + H]^+^): *m/z* calculated ([C_26_H_31_N_3_O_3_ + H]^+^) = 434.2438. Found = 434.2435.

#### 3.4.5. Synthesis of *N*-(2-(((2-Methoxy-6-((2-methyl-[1,1′-biphenyl]-3-yl)methoxy)pyridin-3-yl)methyl)(methyl)amino)ethyl)-*N*-methylacetamide (**2c**)

Lead structure **2** (5.29 mg, 12.6 µmol) was dissolved in 0.5 mL anhydrous DMSO, TBAH (7.17 µL, 15.1 µmol) and methyl iodide (2.36 µL, 37.8 µmol) were subsequently added and the reaction solution was stirred at 100 °C for 20 min. DMSO was removed using solid-phase extraction: A Sep-Pak C18 Plus Light cartridge (Waters Corporation, Germany) was equilibrated in advance with 10 mL MeCN and washed with 20 mL Milli-Q H_2_O. The reaction solution was mixed with 9.5 mL Milli-Q H_2_O and pushed through the column, which was subsequently washed with 5 mL Milli-Q H_2_O. Substances adsorbed on the column material were eluted with 2 mL methanol. A mixture containing **2a** and **2c** was isolated by prep. TLC with 3:2 ethyl acetate/methanol as mobile phase (R_f_ = 0.6) and extracted from the silica particles with methanol. The mixture was further purified by semi-prep. HPLC setup 6. The organic solvent was removed under reduced pressure. The remaining solution was extracted three times with ethyl acetate, and the combined organic phases were washed with Milli-Q H_2_O and dried over Na_2_SO_4_. Ethyl acetate was evaporated at 35 °C facilitated by a slow flow of nitrogen gas, leaving the solid, colorless product (0.46 mg, 8% yield).

Purity: 98.11% as determined by HPLC setup 3, UV detector: 216 nm.

^1^H-NMR (600 MHz, CDCl_3_): δ 7.49–7.40 (m, 4H), 7.36–7.30 (m, 3H), 7.24–7.21 (m, 2H), 6.37 and 6.36 (d*, *J* = 7.9 Hz, 1H), 5.42 and 5.41 (s*, 2H), 3.95z and 3.95 (s*, 3H), 3.53 and 3.39 (m and t*, *J* = 7.2 Hz, 2H), 3.46 (s, 2H), 3.00 and 2.89 (s, 3H), 2.55 (t, *J* = 7.2 Hz, 2H), 2.27 (s, 3H), 2.08 and 2.06 (s*, 3H). Note: Certain peaks are split due to the presence of amide rotamers and are denoted with an asterisk.

ESI-MS ([M + H]^+^): *m/z* calculated ([C_27_H_33_N_3_O_3_ + H]^+^) = 448.2595. Found = 448.2591.

#### 3.4.6. Synthesis of 2-Fluoroethyl (2-Acetamidoethyl)((2-methoxy-6-((2-methyl-[1,1′-biphenyl]-3-yl)methoxy)pyridin-3-yl)methyl)carbamate (**2d**)

Lead structure **2** (10.0 mg, 23.8 µmol) was dissolved in 0.5 mL anhydrous DMSO. Cesium carbonate (25.0 mg, 76.7 µmol) and 2-fluoroethyl *p*-toluenesulfonate (16.3µL, 95.2µmol) were added subsequently, and the resulting solution was stirred at 100 °C for 10 min. The reaction mixture was diluted with 500 µL sodium phosphate buffer pH 7.4 (semi-prep. HPLC solvent “B”), and the product was purified by semi-preparative HPLC using HPLC setup 7. The organic solvent was removed under reduced pressure at elevated temperature (40 °C). The resulting suspension was extracted with ethyl acetate. The organic phase was washed once with Milli-Q H_2_O and brine and dried over Na_2_SO_4_. The organic solvent was removed under reduced pressure yielding a colorless solid (2.7 mg, 25% yield).

Purity: 99.55% as determined by HPLC setup 3, UV detector: 254 nm.

^1^H-NMR (600 MHz, DMSO-d_6_): δ 7.91 (br s, 1H), 7.45 (m, 4H), 7.37 (tt, *J* = 7.5 Hz, *J* = 1.3 Hz, 1H), 7.30 (m, 2H), 7.26 (t, *J* = 7.6 Hz, 1H), 7.18 (dd, *J* = 7.6 Hz, *J* = 1.4 Hz, 1H), 6.43 (d, *J* = 8.0 Hz, 1H), 5.41 (s, 2H), 4.65–4.52 (m, *J* = 48 Hz, 2H), 4.31 (d, *J* = 11 Hz, 2H), 4.27–4.22 (m, *J* = 30 Hz, 2H), 3.91 (s, 3H), 3.24 (t, *J* = 6.6 Hz, 2H), 3.17 (m, 2H), 2.21 (s, 3H), 1.76 (s, 3H).

^13^C-NMR (151 MHz, DMSO-d_6_): δ 169.29, 161.07 (*J* = 7.0 Hz), 159.61, 155.42 (*J* = 16 Hz), 142.14, 141.41, 140.78, 135.85, 133.84, 129.53, 129.15, 128.23, 126.94, 125.47, 110.87 (*J* = 18 Hz), 101.06, 82.03 (*J* = 166 Hz), 66.26, 64.30 (*J* = 19 Hz), 53.33, 46.19 (*J* = 61 Hz), 44.72 (*J* = 20 Hz), 36.85 (*J* = 69 Hz), 22.52 (*J* = 7.0 Hz), 15.91.

^19^F-NMR (565 MHz, DMSO-d_6_): δ −223.13 (tt, *J* = 48 Hz, *J* = 30 Hz, 1F).

ESI-MS ([M + H]^+^): *m/z* calculated ([C_28_H_32_FN_3_O_5_ + H]^+^) = 510.2399. Found = 510.2428.

### 3.5. In-Solution Stability Measurements

For stability measurements of lead structures **1** and **2** as well as substances **1a, 1b, 2a, 2b**, **2c** and **2d,** each compound was dissolved separately in 1:1 DMSO/HEPES buffer. The buffer contained 10 mM HEPES (Merck KGaA, Darmstadt, Germany) adjusted to pH 7.5 with NaOH and 150 mM NaCl. The solutions were stored in an amber glass vial (Thermo Fisher Scientific Inc., Waltham, MA, USA) at room temperature (24 °C) or in the fridge (0 °C) and analyzed over a period of 3 weeks via HPLC, measuring three technical replicates. HPLC setup 1 was used for **1a** and **1b** and HPLC setup 3 for **1**, **2**, **2a, 2b, 2c** and **2d**. Detection was performed at 216 nm. The area under the curve (AUC) was used as the evaluation parameter and plotted over time.

### 3.6. Lipophilicity and Calculated Physicochemical Properties

The measurements of lipophilicity of precursors and products were performed according to the HPLC method of Donovan and Pescatore [24] and Vraka et al. [25]. An internal standard mixture consisting of 1% *v/v* toluene (Merck KGaA, Darmstadt, Germany) and 0.438 mmol/L triphenylene (Merck KGaA, Darmstadt, Germany) in methanol was added to sample solutions of approx. 1 mg/mL dissolved in DMSO.

After separation by HPLC setup 10 and determination of retention times by simultaneous detection at 254 and 280 nm in three technical replicates, the calculation of ${\log P}_{\mathrm{OW}}^{\mathrm{pH}\;7.4}$ (log*D*) was performed as described before [25]. Three log*P* values of the reference substances were taken from the literature, resulting in a mean log*P* value of the analyte (${µ\mathrm{HPLC}\;\log P}_{\mathrm{OW}}^{\mathrm{pH}\;7.4}{}_{\mathrm{analyte}})$. Furthermore, the values were compared to calculated log*P* (clog*P*) and topological polar surface area (tPSA) values from ChemDraw 20.1 (PerkinElmer, Inc., Waltham, MA, USA) as well as clog*D*_pH7.4_ values from MarvinSketch 22.13 (ChemAxon Ltd., Budapest, Hungary).

### 3.7. Binding Affinity Measurements

A commercially available homogeneous time-resolved fluorescence (HTRF) PD-1/PD-L1 Binding Assay Kit (Cisbio Bioassays SAS, Codolet, France, part no. 64PD1PEG) was used to determine in vitro binding affinities towards human PD-L1. The assay was prepared and performed according to the binding assay kit protocol using white, flat-bottom, low-volume Greiner 384-well plates (Merck KGaA, Darmstadt, Germany) and an HTRF-compatible Flexstation 3 Multi-Mode Microplate Reader (Molecular Devices LLC., San Jose, CA, USA) for read-out. Tenfold dilution series of the small-molecule compounds were prepared at a constant final DMSO concentration of 0.2%, as it is recommended to keep DMSO below 0.5% (Supplementary Figure S1). A threefold dilution series without DMSO was used for the antibody atezolizumab (MedChemExpress, Monmouth Junction, NJ, USA) and a tenfold dilution series without DMSO for the peptide PD-L1/PD-1 Inhibitor 3 (Selleck Chemicals Llc, Houston, TX, USA). Assay validation was monitored using the provided PD-1/PD-L1 antibody from the assay kit. Experiments were repeated for a total of three times. IC_50_ calculation was performed with GraphPad Prism 8 (GraphPad Software, Inc., Boston, MA, USA) using the variable slope (four parameters) dose–response fit. Data normalization was performed for inter-assay comparison of multiple experiments according to the procedure advised by Cisbio. The ratio between the detected wavelengths was calculated (Formula (1)), and the background fluorescence signal was subtracted from the ratio to obtain the delta ratio (ΔR) (Formula (2)) from which delta F (ΔF), which reflects the signal to background ratio of the assay, can be calculated (Formula (3)). For normalization, ΔF/ΔF_max_ was calculated (Formula (4)) and plotted against the sample concentration on a logarithmic scale. Data normalization had no impact on IC_50_ calculation.

(1) $$\mathrm{Ratio}=\frac{A\;(665\;\mathrm{nm})}{B\;(620\;\mathrm{nm})}{\;\times 10}^{4}$$

(2) $$\Delta {R=\mathrm{Ratio}}_{\mathrm{sample}}-{\;\mathrm{Ratio}}_{\mathrm{background}}$$

(3) $$\Delta F=\frac{{\mathrm{Ratio}}_{\mathrm{sample}}-{\;\mathrm{Ratio}}_{\mathrm{background}}}{{\mathrm{Ratio}}_{\mathrm{background}}}\%$$

(4) $$y_{\mathrm{normalized}}=\frac{\Delta F}{\Delta F_{\max}}$$

### 3.8. Radiosyntheses with Carbon-11

Small scale radiosyntheses were performed using a GE TRACERlab™ FX2 C module (General Electric Medical Systems, Uppsala, Sweden). Radionuclide production and production of [^11^C]methylating agents was performed as described before [26]. In short, [^11^C]CO_2_ was produced in a GE PETtrace cyclotron (General Electric Medical Systems, Uppsala, Sweden) by irradiation of a gas target containing N_2_ and 0.5% O_2_ using the ^14^N(p,α)^11^C nuclear reaction with up to 16.5 MeV protons. [^11^C]CO_2_ was reduced to [^11^C]CH_4_ by H_2_ gas and nano-powdered nickel as a catalyst at 400 °C. [^11^C]CH_4_ was converted into [^11^C]CH_3_I with I_2_ at 720–740 °C by a radical reaction. Subsequently, [^11^C]CH_3_I was trapped in the solvent (i.e., MeCN or DMSO). Alternatively, [^11^C]CH_3_I was passed through a silver triflate containing column at 200 °C for [^11^C]CH_3_OTf production, which was then used as a methylation reagent. A total of 100 µL of the [^11^C]CH_3_I or [^11^C]CH_3_OTf containing solution was added to the precursor solution.

For the radiosynthesis of **[^11^C]1a**, compound **1** was used as a precursor, and radiolabeling was performed with 100 µL [^11^C]CH_3_I solution, 1 mg precursor in 400 µL DMSO and 1 eq. tetrabutylammonium hydroxide (TBAH) at 60 or 150 °C for 2 or 4 min.

For the radiosynthesis of **[^11^C]2a** and **[^11^C]2b,** 100 µL [^11^C]CH_3_I solution was added to 0.5 or 1 mg of compound **2** dissolved in 400 µL MeCN or DMSO and stirred at room temperature, 60 °C, 100 °C or 150 °C for 2 or 4 min. Using [^11^C]CH_3_OTf, reactions were conducted at 150 °C for 4 min with and without 1 eq. TBAH as a base. MeCN was used for radiolabeling at room temperature and 60 °C, whereas DMSO was used for reactions at 100 °C and 150 °C.

Eventually, the reactions were quenched with 100 µL H_2_O, and the radiochemical conversion (RCC) was determined by HPLC using setup 8 and setup 9 for **[^11^C]1a** and **[^11^C]2a**, respectively.

For further in vitro evaluation, **[^11^C]2a** was purified by semi-prep. HPLC (HPLC setup 11). Organic solvent was removed by solid-phase extraction: The product fraction was diluted with 40 mL H_2_O, transferred onto a Sep-Pak C18 Plus Short cartridge (Waters Corporation, Germany), washed with 10 mL H_2_O and eluted with 1.5 mL ethanol followed by 5 mL saline. Radiochemical purity was determined by HPLC setup 9.

### 3.9. Metabolic Stability

Metabolic stability was assessed using pooled, mixed gender human liver microsomes (HLMs) (Corning, Corning, NY, USA) according to the supplied protocol. In short, 15 µL of HLM (20 mg/mL), 15 µL of NADPH regenerating system solution A (Corning, Corning, NY, USA), 3 µL of solution B (Corning, Corning, NY, USA) and 257 µL of a 1:10 dilution of 10× PBS concentrate (MORPHISTO, Offenbach am Main, Germany) were pre-incubated at 37 °C for 10 min. Aliquots were drawn 0, 10, 20, 30 and 40 min after addition of 10 µL radiotracer, subsequently quenched with the same amount of MeCN, centrifuged for 4 min at 21,500× *g* and analyzed by HPLC setup 3.

### 3.10. Statistical Analysis

Values are depicted as mean ± standard deviation (SD), and experiments were performed in triplicates and repeated at least three times. Peak areas in the radioactivity channel were corrected for decay during HPLC measurements, and radiochemical conversion was calculated according to Equation (5).

(5) $$\mathrm{RCC}\;\left[\%\right]=\frac{\frac{A_{x}}{e^{\left(-\frac{\ln\left(2\right)}{20.364}{*\mathrm{Rt}}_{x}\right)}}}{\sum_{i=1}^{n}\left(\frac{A_{i}}{e^{\left(-\frac{\ln\left(2\right)}{20.364}{*\mathrm{Rt}}_{i}\right)}}\right)+\frac{A_{x}}{e^{\left(-\frac{\ln\left(2\right)}{20.364}{*\mathrm{Rt}}_{x}\right)}}}*100$$

where:

A: peak area;

Rt: retention time (min);

x: substance of interest;

i: other entities.

Radioactive decay correction of radiochemical conversion (RCC) occurred during HPLC measurements.

Spearman’s rank correlation was calculated in Microsoft Excel (Version 2207; Microsoft Corporation, Redmond, WA, USA). Correlation categorization was adapted from Dancey and Reidy [27]: weak: 0.1–0.39, moderate: 0.4–0.69, strong: 0.7–0.9. A confidence interval of 95% was applied.

## 4. Conclusions

A total of six small molecules, derived from a ligand-based drug design strategy, were successfully synthesized and comprehensively characterized for their physicochemical properties, stability and binding affinity towards human programmed death-ligand 1 (*h*PD-L1). Employing an extensive small-scale radiolabeling investigation, we achieved selective labeling of constitutional isomers, wherein the carbon-11 label was introduced selectively onto either an amine (**[^11^C]2a**) or an amide functionality (**[^11^C]2b**) with remarkable radiochemical conversion (RCC) exceeding 50%. Although the pursuit of small-molecule ligands for *h*PD-L1 with sufficiently high affinities for PET imaging applications remains a challenge, this study significantly expanded our understanding of the influence of structural modifications of compounds based on the (2-methyl-[1,1′-biphenyl]-3-yl)methanol core scaffold and their monoselective carbon-11 N-methylation of amides in the presence of amines.

## Figures and Tables

**Table 1:** Results of ligand docking experiments (mean ± standard deviation). Lower binding affinity scores and affinity values indicate better binding affinity.

Substance	Binding Affinity Score	Affinity (kcal/mol)
**1**	−37.35 ± 1.21	−9.93 ± 0.99
**1a**	−30.70 ± 0.20	−9.57 ± 0.17
**1b**	−37.68 ± 3.52	−10.3 ± 0.06
**2**	−37.96 ± 2.02	−10.2 ± 0.21
**2a**	−30.85 ± 2.19	−9.80 ± 0.20
**2b**	−31.12 ± 3.67	−9.73 ± 0.25
**2c**	−33.44 ± 1.86	−9.63 ± 0.76
**2d**	−35.52 ± 1.51	−10.2 ± 0.06

**Table 2:** Calculated log*P* (clog*P*), clog*D*, tPSA and measured HPLC log*D* values.

Substance	clog*P*	clog*D*	tPSA (Å^2^)	${µ\mathrm{HPLC}\;\log P}_{\mathrm{OW}}^{\mathrm{pH}\;7.4}$
**1**	4.60	2.73	68.23	3.16 ± 0.16
**1a**	6.93	6.01	57.23	4.90 ± 0.27
**1b**	7.18	6.21	57.23	5.02 ± 0.28
**2**	4.41	3.64	71.95	3.88 ± 0.12
**2a**	4.95	4.42	63.16	4.13 ± 0.16
**2b**	4.81	3.83	63.16	3.97 ± 0.13
**2c**	5.35	4.64	54.37	4.28 ± 0.18
**2d**	5.37	4.52	89.46	4.23 ± 0.17

**Table 3:** Measured PD-L1 binding affinities (IC_50_ values) of the lead structures (**1** and **2**), methylated or fluoroethylated derivatives as well as the antibody atezolizumab and the peptide PD-1/PD-L1 Inhibitor 3 as positive controls using the commercially available HTRF assay. * No full dose–response curves were observed.

	IC_50_ (nM)		
Substance	BMS [11]	Cisbio [22]	This Work
**Atezolizumab**		3.84	4.07 ± 0.42
**1**	6–100	177	202 ± 27
**1a**			5760 ± 613
**1b**			1440 ± 144
**2**	18		101 ± 10
**2a**			430 ± 62
**2b**			524 ± 67
**2c**			1310 ± 185
**2d**			9880 ± 1390 *
**Inhibitor 3**		146	113 ± 45

**Table 4:** Efficiency indices used to guide drug development. LE = ligand efficiency, BEI = binding efficiency index, LLE = ligand lipophilicity efficiency, LELP = ligand-efficiency-dependent lipophilicity efficiency. Indices were calculated as described before [22,23]: LE = −1.4(logIC_50_)/N_heavy atoms_, BEI = −logIC_50_/(molecular weight/1000), LLE = −logIC_50_ − ${µ\mathrm{HPLC}\;\log P}_{\mathrm{OW}}^{\mathrm{pH}\;7.4}$, LELP = ${µ\mathrm{HPLC}\;\log P}_{\mathrm{OW}}^{\mathrm{pH}\;7.4}/\mathrm{LE}$.

Substance	LE (kcal/mol/Heavy Atom)	BEI	LLE	LELP
**1**	0.27	14.1	3.5	11.8
**1a**	0.20	10.7	0.3	24.0
**1b**	0.22	11.4	0.9	22.9
**2**	0.32	16.7	3.1	12.3
**2a**	0.28	14.7	2.2	14.8
**2b**	0.27	14.5	2.3	14.4
**2c**	0.25	13.1	1.6	17.1
**2d**	0.19	9.9	0.8	22.2

## Data Availability

Data is contained within the article or Supplementary Materials.

## Supplementary Materials

The following supporting information can be downloaded at: https://www.mdpi.com/article/10.3390/ph16071051/s1. Ligand docking poses, in-solution stability data, HTRF binding assay curves, and Spearman’s rank correlation coefficient calculations for lead structures and final compounds. Small-scale radiosynthesis data for **[^11^C]2a** and **[^11^C]2b**. Copies of ^1^H, ^13^C, ^19^F and 2D NMR spectra, HR-MS spectra as well as HPLC chromatograms of final compounds. Figure S1: Influence of DMSO concentration on HTRF measurements. Figure S2: Docking pose and pharmacophore of lead structure **1**. Figure S3: Docking pose and pharmacophore of **1a**. Figure S4: Docking pose and pharmacophore of **1b**. Figure S5: Docking pose and pharmacophore of **2a**. Figure S6: Docking pose and pharmacophore of **2b**. Figure S7: Docking pose and pharmacophore of **2c**. Figure S8: Docking pose and pharmacophore of **2d**. Table S1: Overview of synthesis attempts using different reactions and reaction conditions. Figure S9: Interday stability of lead structure **1**. Figure S10: Interday stability of lead structure **2**. Figure S11: Interday stability of **1a**. Figure S12: Interday stability of **1b**. Figure S13: Interday stability of **2a**. Figure S14: Interday stability of **2b**. Figure S15: Interday stability of **2c**. Figure S16: Interday stability of **2d**. Figure S17: Representative binding affinity curves of lead structures, final compounds, and reference compounds. Table S2: Spearman’s rank correlation coefficient of physico-chemical properties, ligand docking parameters and measured HTRF IC50 values. Figure S18: Small-scale radiosynthesis and radiochemical conversions of **[^11^C]2a** and **[^11^C]2b**. Figure S19: HPLC purity of **1a**. Figure S20: HPLC purity of **1b**. Figure S21: HPLC purity of **2a**. Figure S22: HPLC purity of **2b**. Figure S23: HPLC purity of **2c**. Figure S24: HPLC purity of **2d**. Figure S25: ^1^H-NMR (600 MHz, CDCl_3_) spectrum of **1a**. Figure S26: ^13^C-NMR (151 MHz, CDCl_3_) spectrum of **1a**. Figure S27: ^1^H-^1^H COSY NMR (600 MHz, CDCl_3_) spectrum of **1a**. Figure S28: ^1^H-^13^C HSQC NMR (600 MHz, 151 MHz, CDCl_3_) spectrum of **1a**. Figure S29: ^1^H-NMR (600 MHz, CDCl_3_) spectrum of **1b**. Figure S30: ^13^C-NMR (151 MHz, CDCl_3_) spectrum of **1b**. Figure S31: ^19^F-NMR (470 MHz, CDCl_3_) spectrum of **1b**. Figure S32: ^1^H-NMR (400 MHz, CDCl_3_) spectrum of **2a**. Figure S33: ^1^H-NMR (600 MHz, CDCl_3_) spectrum of **2b**. Figure S34: ^1^H-NMR (600 MHz, CDCl_3_) spectrum of **2c**. Figure S35: ^1^H-NMR (600 MHz, DMSO-d_6_) spectrum of **2d**. Figure S36: ^13^C-NMR (151 MHz, DMSO-d_6_) spectrum of **2d**. Figure S37: ^19^F-NMR (565 MHz, DMSO-d_6_) spectrum of **2d**. Figure S38: ^1^H-^1^H COSY NMR (600 MHz, DMSO-d_6_) spectrum of **2d**. Figure S39: ^1^H-^13^C HSQC NMR (600 MHz, 151 MHz, DMSO-d_6_) spectrum of **2d**. Figure S40: HR-MS spectrum of **1a**. Figure S41: HR-MS spectrum of **1b**. Figure S42: HR-MS spectrum of **2a**. Figure S43: HR-MS spectrum of **2b**. Figure S44: HR-MS spectrum of **2c**. Figure S45: HR-MS spectrum of **2d**.
