# Supplementary material for: On the Road towards Small-Molecule Programmed Cell Death 1 Ligand 1 Positron Emission Tomography Tracers: A Ligand-Based Drug Design Approach
Source: Pharmaceuticals (Basel). 2023 Jul 24;16(7):1051. doi: 10.3390/ph16071051 (PMC10385977; doi:10.3390/ph16071051)

Supplementary information

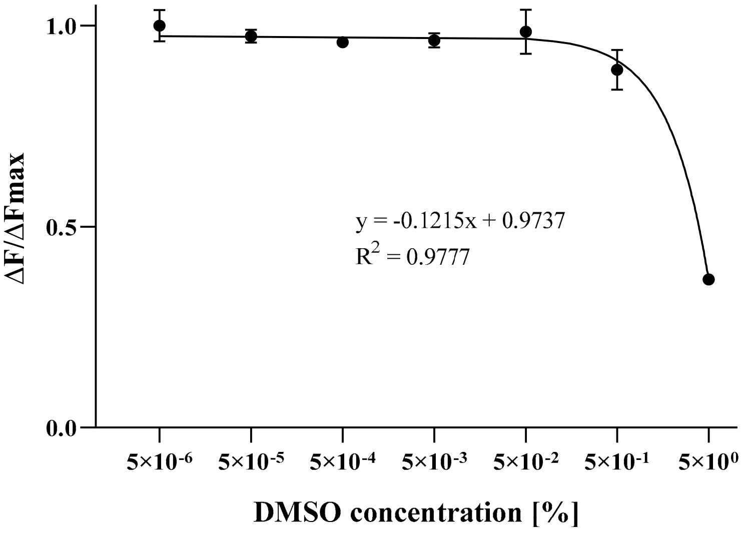

Supplementary Figure S1: Influence of DMSO concentration on HTRF measurements. DMSO significantly quenched the fluorescence intensity at 665 nm but not 620 nm above 0.5% concentrations.

Ligand docking experiments

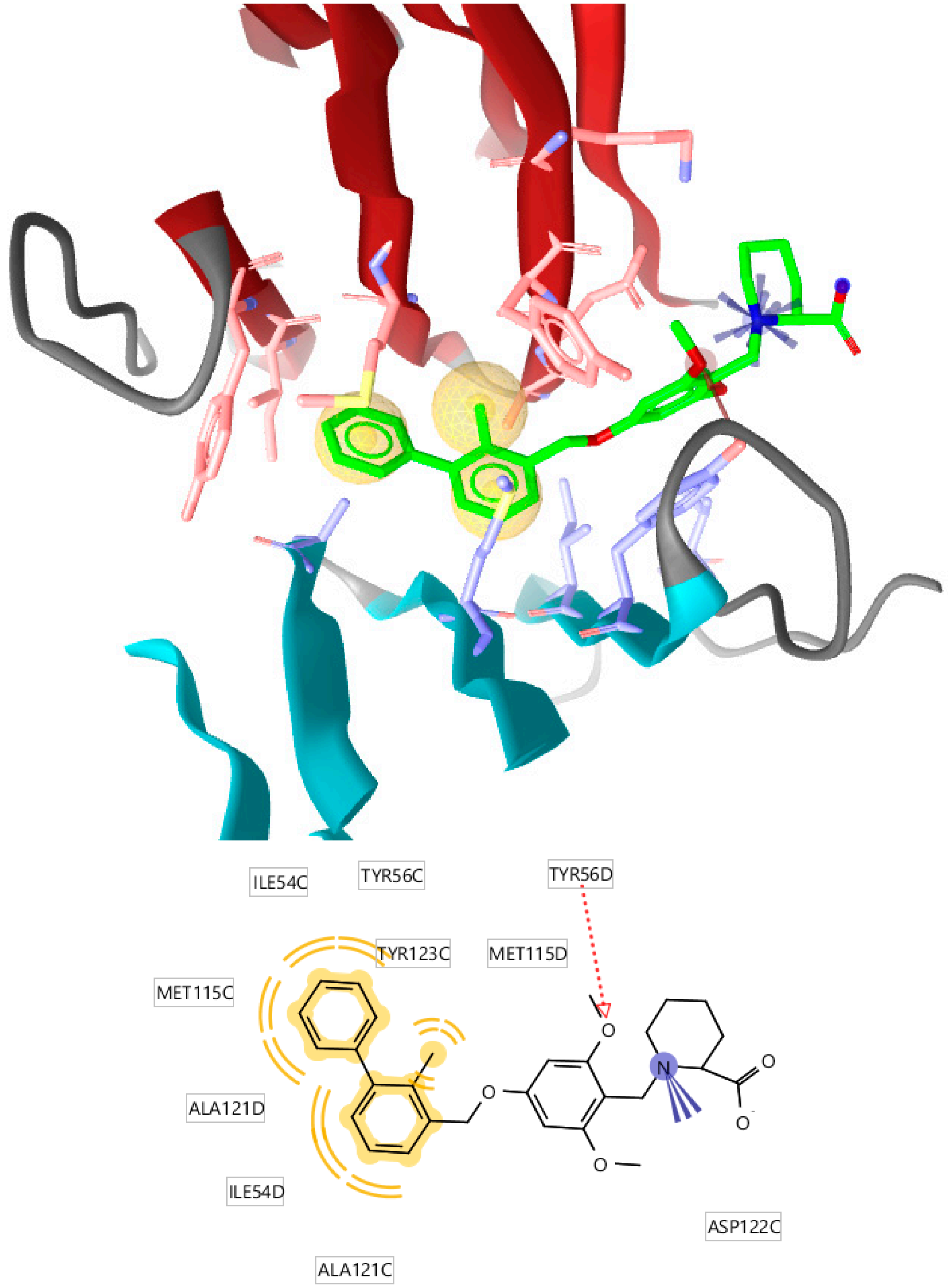

Supplementary Figure S2: Docking pose and pharmacophore of lead structure **1** (green) between PD-L1 monomer C (red) and monomer D (blue).

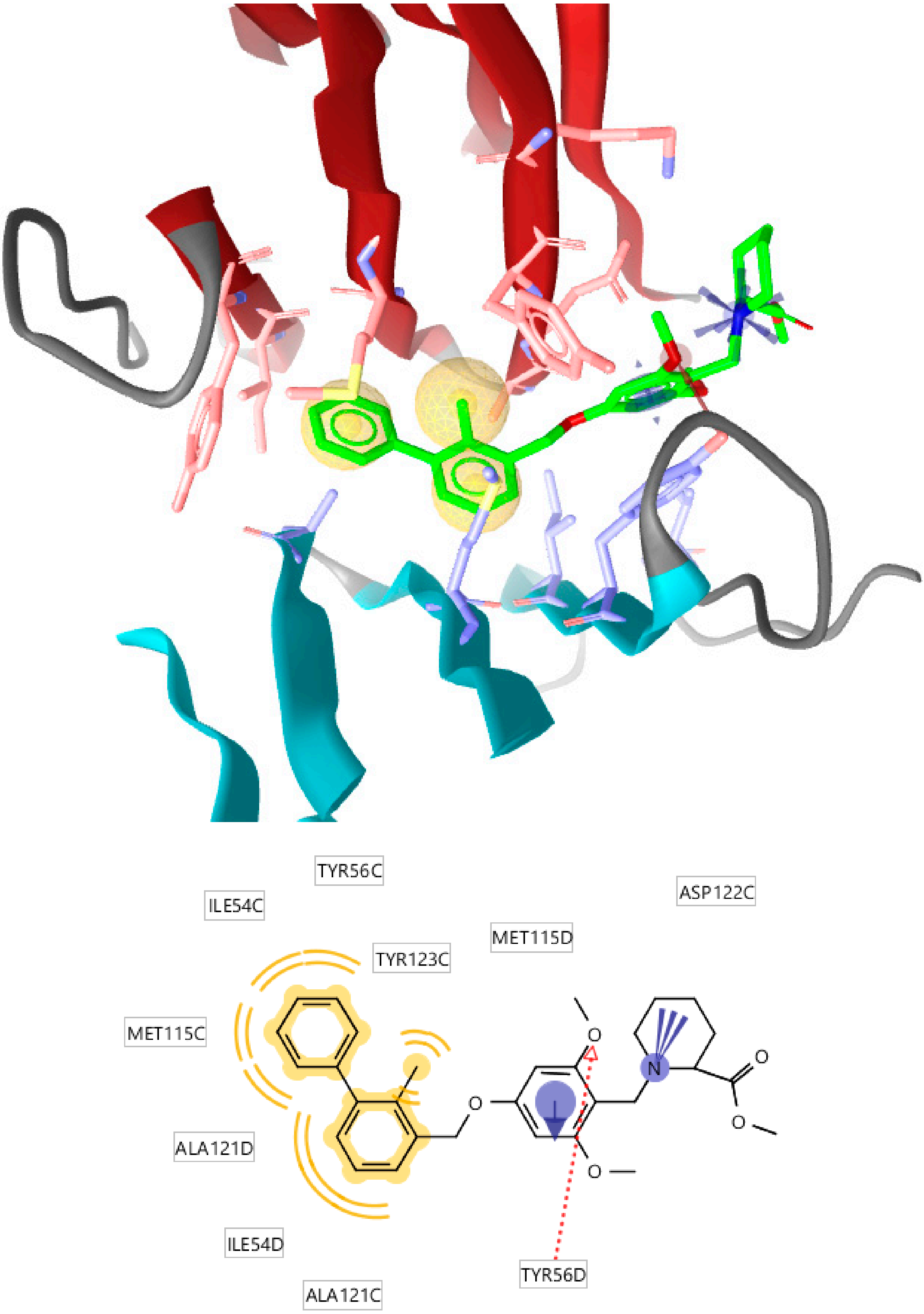

Supplementary Figure S3: Docking pose and pharmacophore of **1a** (green) between PD-L1 monomer C (red) and monomer D (blue).

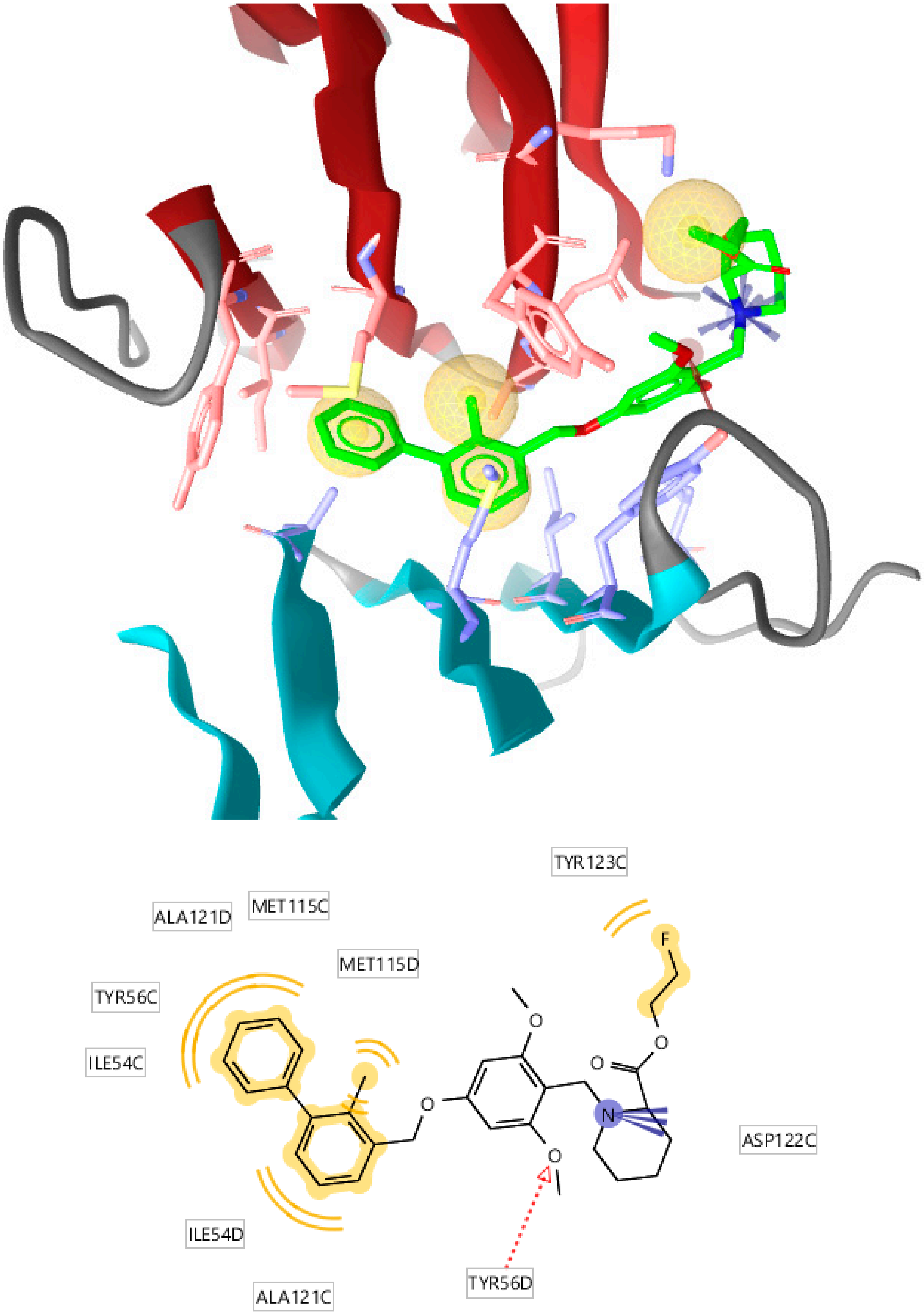

Supplementary Figure S4: Docking pose and pharmacophore of **1b** (green) between PD-L1 monomer C (red) and monomer D (blue).

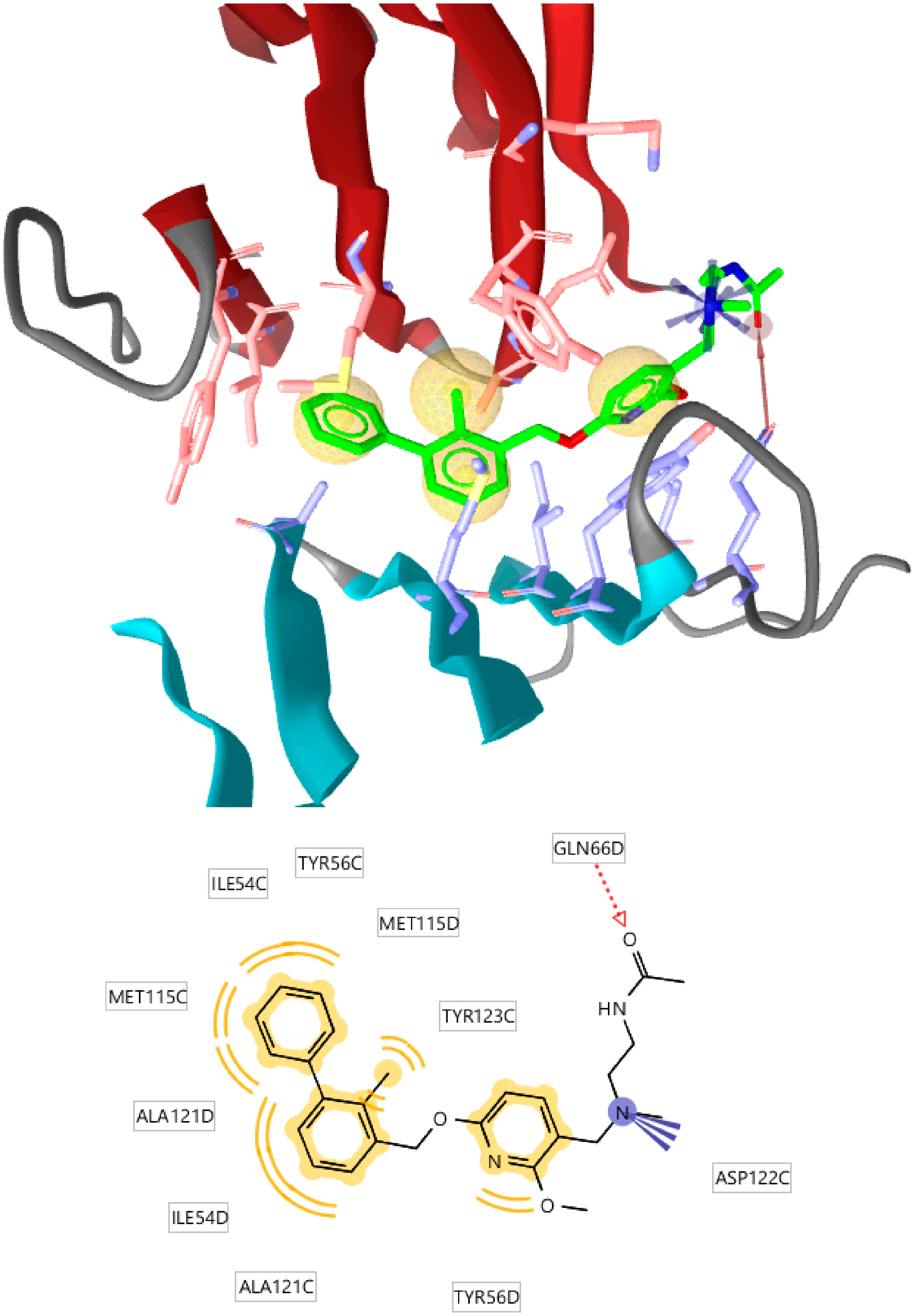

Supplementary Figure S5: Docking pose and pharmacophore of **2a** (green) between PD-L1 monomer C (red) and monomer D (blue).

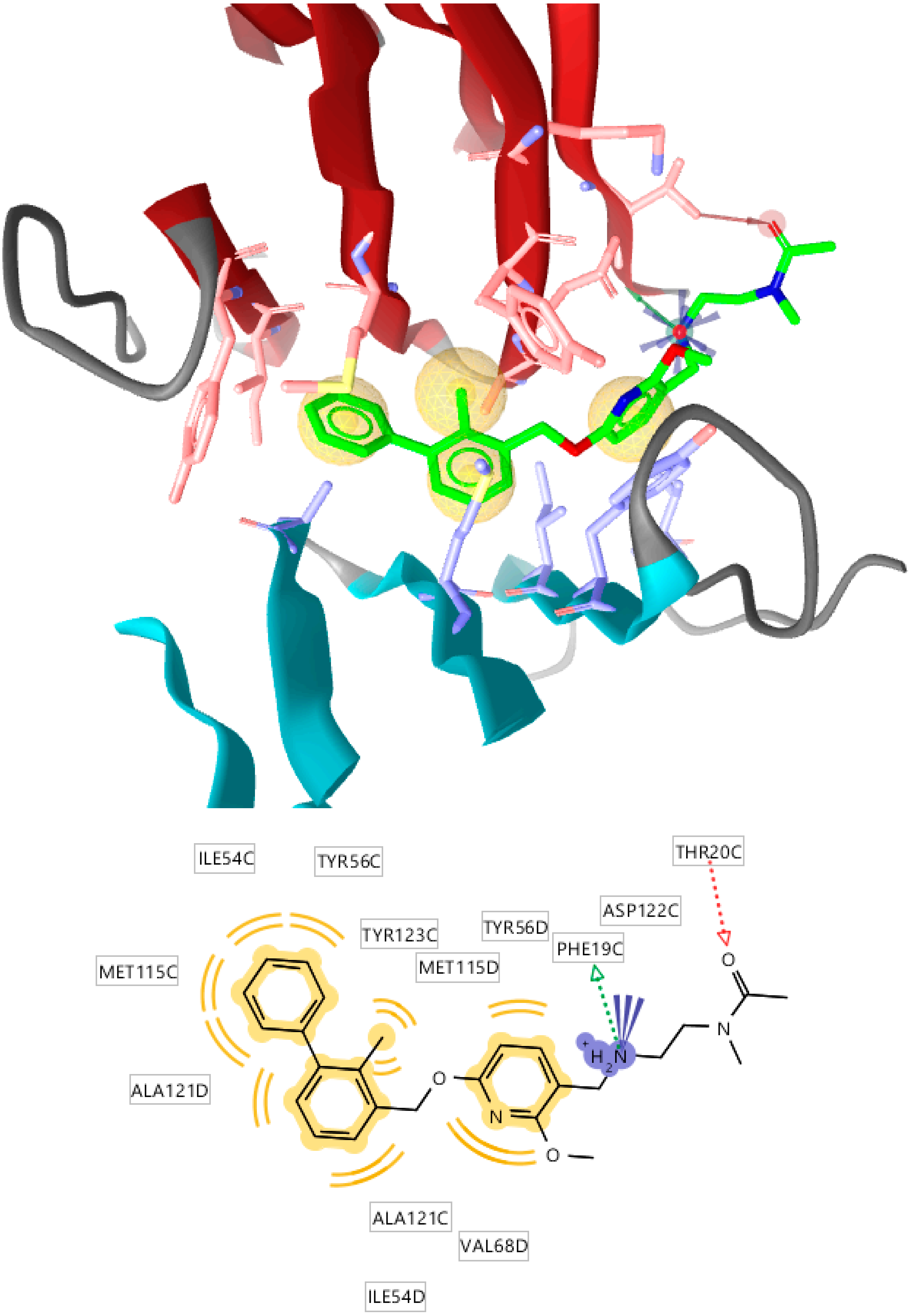

Supplementary Figure S6: Docking pose and pharmacophore of **2b** (green) between PD-L1 monomer C (red) and monomer D (blue).

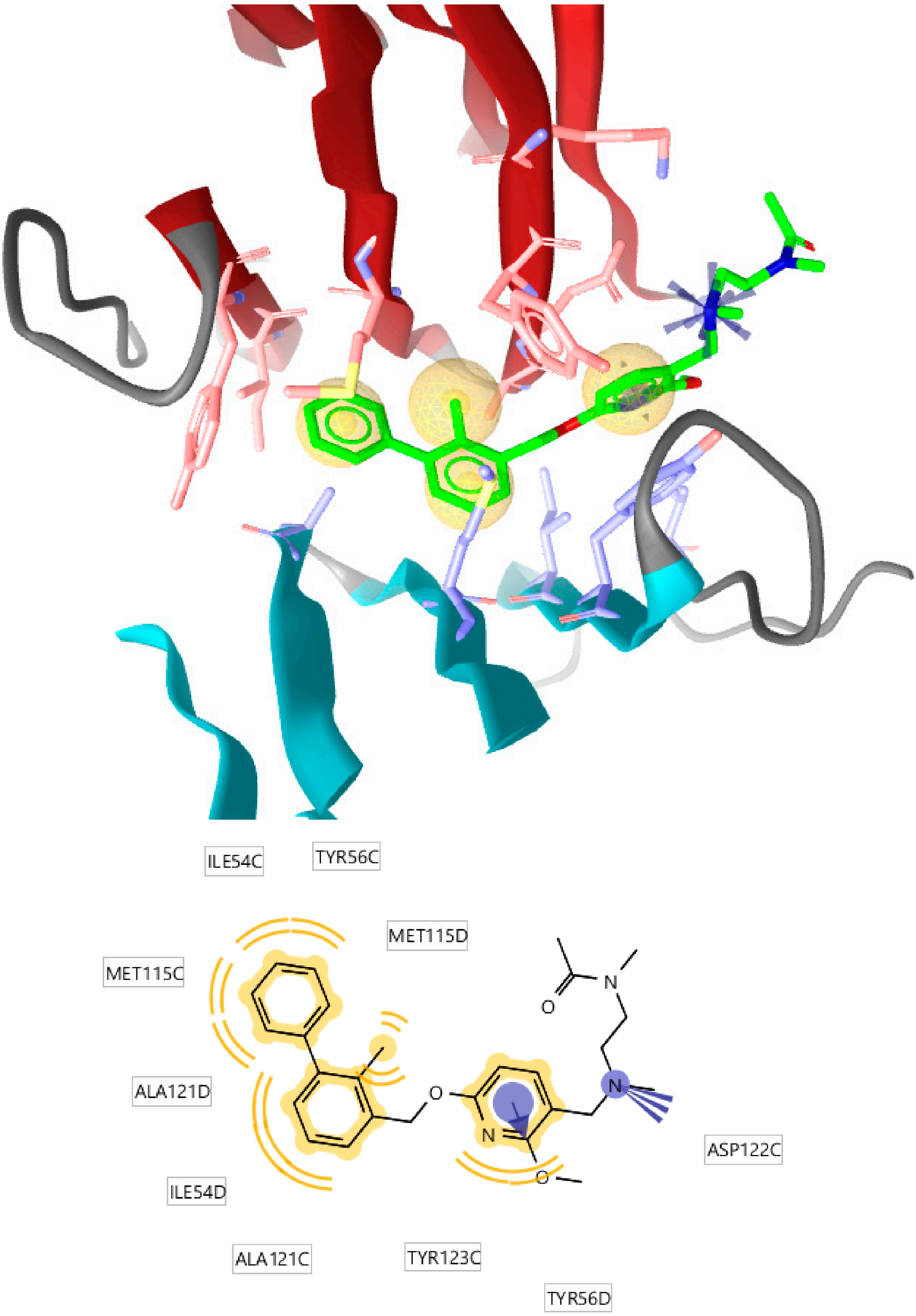

Supplementary Figure S7: Docking pose and pharmacophore of **2c** (green) between PD-L1 monomer C (red) and monomer D (blue).

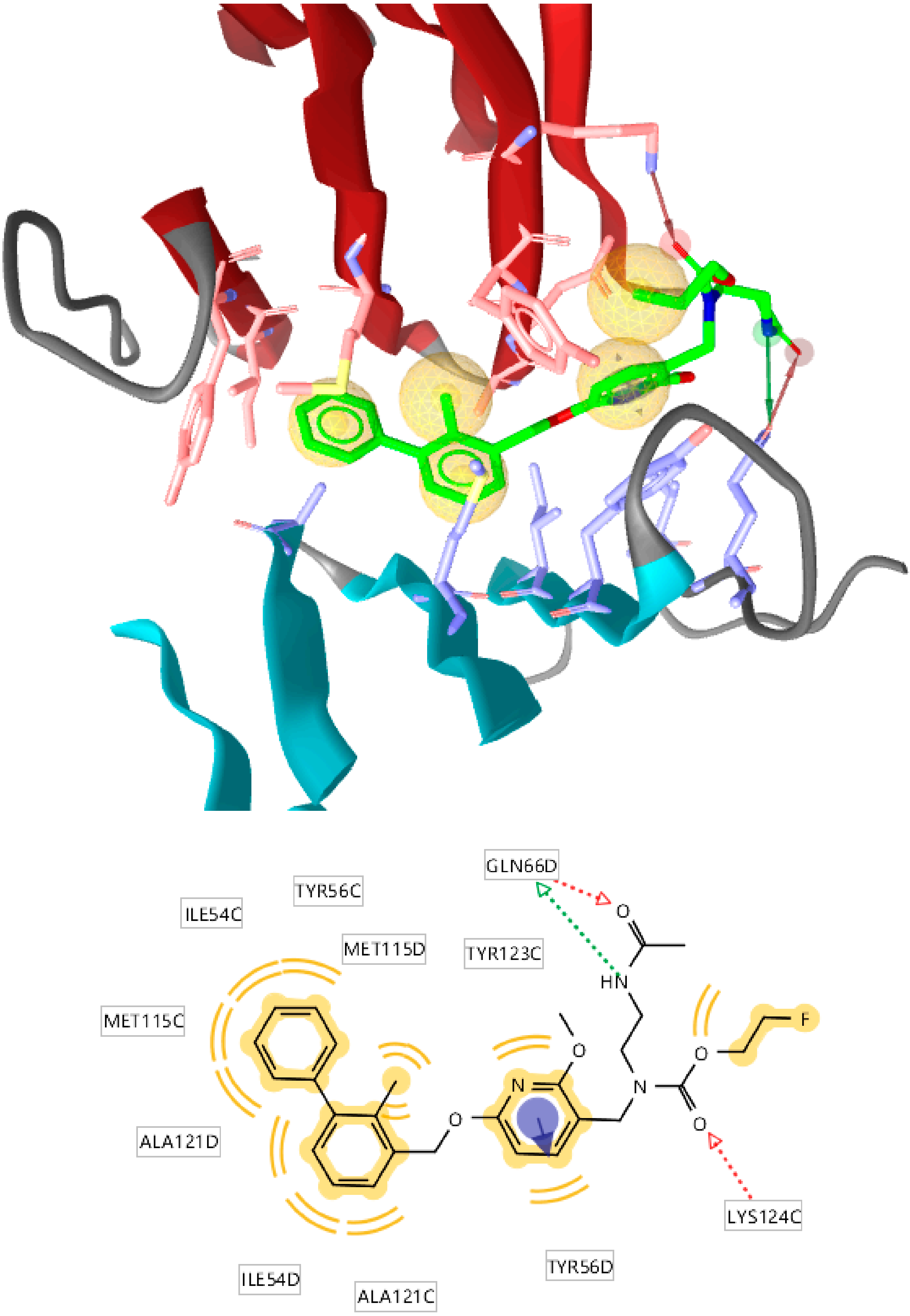

Supplementary Figure S8: Docking pose and pharmacophore of **2d** (green) between PD-L1 monomer C (red) and monomer D (blue).

Supplementary Table S1: Overview of synthesis attempts using different reactions and reaction conditions, including the chemical structures of the lead compounds **1** and **2** as well as the six synthesized products: monomethylated products **1a**, **2a** and **2b**, dimethylated product **2c**, fluoroethylated product **1b** as well as product **2d** with a fluoroethyl carbamate unit. DCM = dichloromethane, DMAP = 4-dimethylaminopyridine, DCC = N,N′-dicyclohexylcarbodiimide, CDI = 1,1'-carbonyldiimidazole, MeI = methyl iodide, ODCB = 1,2-dichlorobenzene, DTBP = di-*tert*-butyl peroxide, TBAH = tetrabutylammonium hydroxide, FETos = 2-fluoroethyl *p*-toluenesulfonate, DIPEA = *N,N*-diisopropylethylamine.

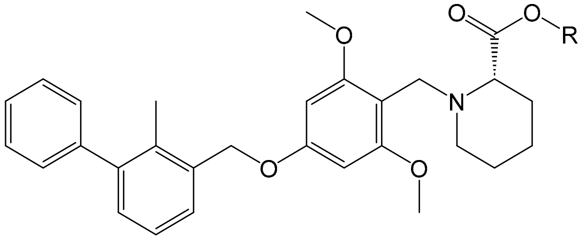

1: R = -H;
1a: R = $-CH_3$;
1b: R = $-C_2H_4F$;

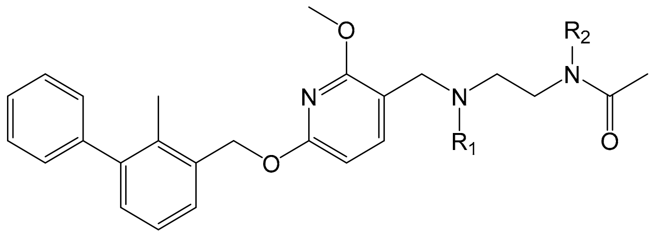

2: R_1 = -H, R_2 = -H;
2a: R_1 = $-CH_3$, R_2 = -H;
2b: R_1 = -H, R_2 = $-CH_3$;
2c: R_1 = $-CH_3$, R_2 = $-CH_3$;
2d: R_1 = $-CO_2C_2H_4F$, R_2 = -H;
Boc-2: R_1 = -Boc, R_2 = -H;
Me-Boc-2: R_1 = -Boc, R_2 = $-CH_3$;
FE-2: R_1 = $-C_2H_4F$, R_2 = -H;

Entry	Substrate	Product	Solvent	Reagent(s) (equivalent)	Temperature	Time	Yield [%]	Reaction
**1**	**1**	**1a**	MeOH	H_2SO_4 (cat.)	reflux	up to 24 h	0	Fischer esterification
**2**	**1**	**1a**	MeOH	$SOCl_2$ (1.1 eq.)	rt	up to 48 h	0	Conversion into acyl chloride
**3**	**1**	**1a**	DCM	MeOH (3.5 eq.), DMAP (cat.), DCC (1.1)	0°C → rt	up to 24 h	19	Steglich esterification
**4**	**1**	**1a**	THF	1) CDI (2.7 eq.), 2) MeOH (1.0 eq.)	50°C	5 h	29	Conversion into acyl imidazole
**5**	**1**	**1a**	DMSO	Cs_2CO_3 (1.5 eq.), MeI (3.0 eq.)	60°C	24 h	5	Nucleophilic substitution

**6**	**1**	**1a**	ODCB	CuI (cat.), DTBP (2.0 eq.)	110°C	up to 24 h	0	Metal-catalysed O-methylation
**7**	**1**	**1b**	DMSO	Cs_2CO_3 (3.2 eq.), FETos (4.0 eq.)	100°C	10 min	61	Nucleophilic substitution
**8**	**2**	**2a**	MeCN	MeI (3.0 eq.)	50°C	30 min	41	Nucleophilic substitution
**9**	**2**	**2a**	ODCB	CuI (cat.), DTBP (2.0 eq.)	110°C	up to 24 h	0	Metal-catalysed N-methylation
**10**	**2**	**Boc-2**	THF	Boc_2O (1.2 eq.)	rt	24 h	85	Boc protection
**11**	**Boc-I2**	**Me-Boc-2**	MeCN	TBAH (5.0 eq.), MeI (6.0 eq.)	100°C	10 min	9	Nucleophilic substitution
**12**	**Me-Boc-2**	**2b**	MeCN	conc. HCl (excess)	rt	24 h	68	Boc deprotection
**13**	**2**	**2c**	DMSO	TBAH (1.2 eq.), MeI (3.0 eq.)	100°C	20 min	8	Nucleophilic substitution
**14**	**2**	**FE-2**	DMF	DIPEA (1.0 eq.), FETos (1.2 eq.)	60°C	up to 24 h	0	Nucleophilic substitution
**15**	**2**	**FE-2**	DMF	DIPEA (1.0 eq.), FETos (1.2 eq.)	100°C	up to 24 h	0	Nucleophilic substitution
**16**	**2**	**FE-2**	DMSO	KO^tBu (3.0 eq.), FETos (1.2 eq.)	100°C	20 min	0	Nucleophilic substitution
**17**	**2**	**2d**	DMSO	Cs_2CO_3 (3.2 eq.), FETos (4.0 eq.)	100°C	10 min	75	Carbamylation and nucleophilic substitution

In-solution stability

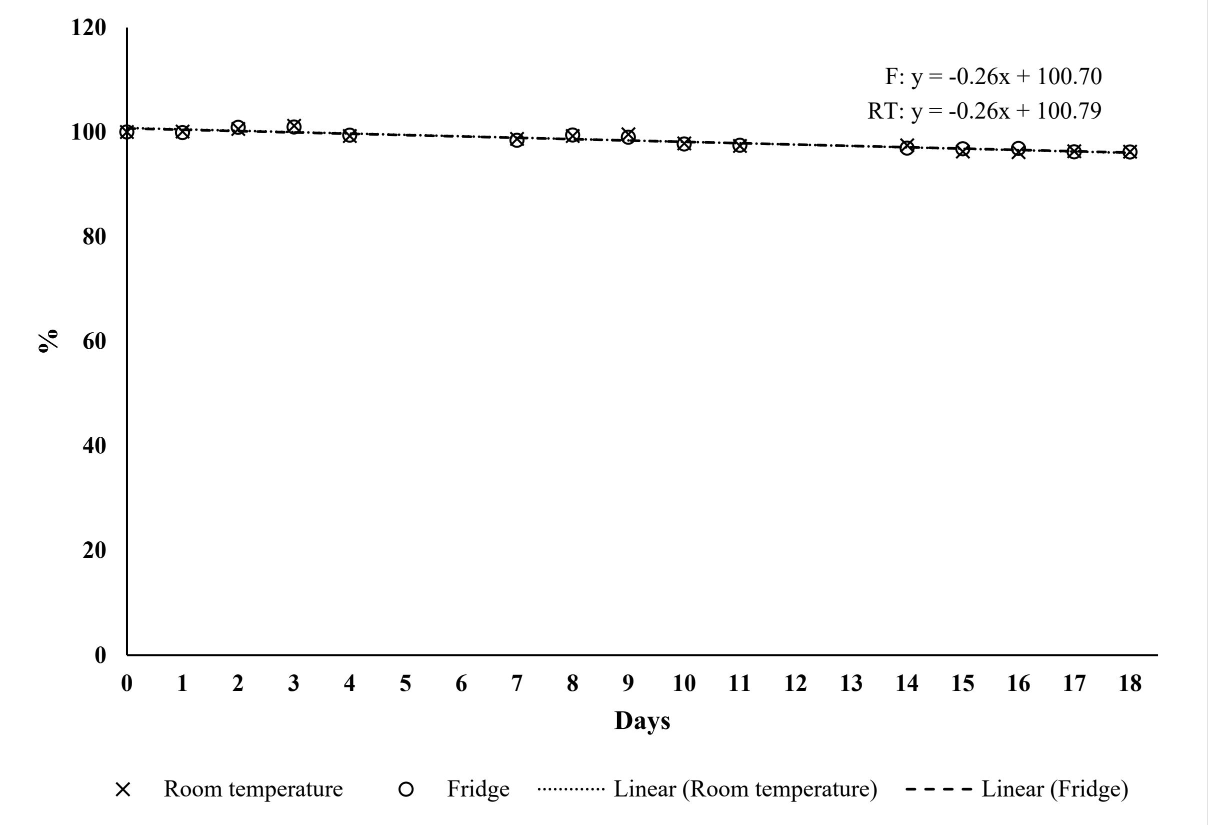

Supplementary Figure S9: Interday stability of lead structure **1** in 1:1 DMSO and 10 mM HEPES-NaOH pH 7.5 with 150 mM NaCl.

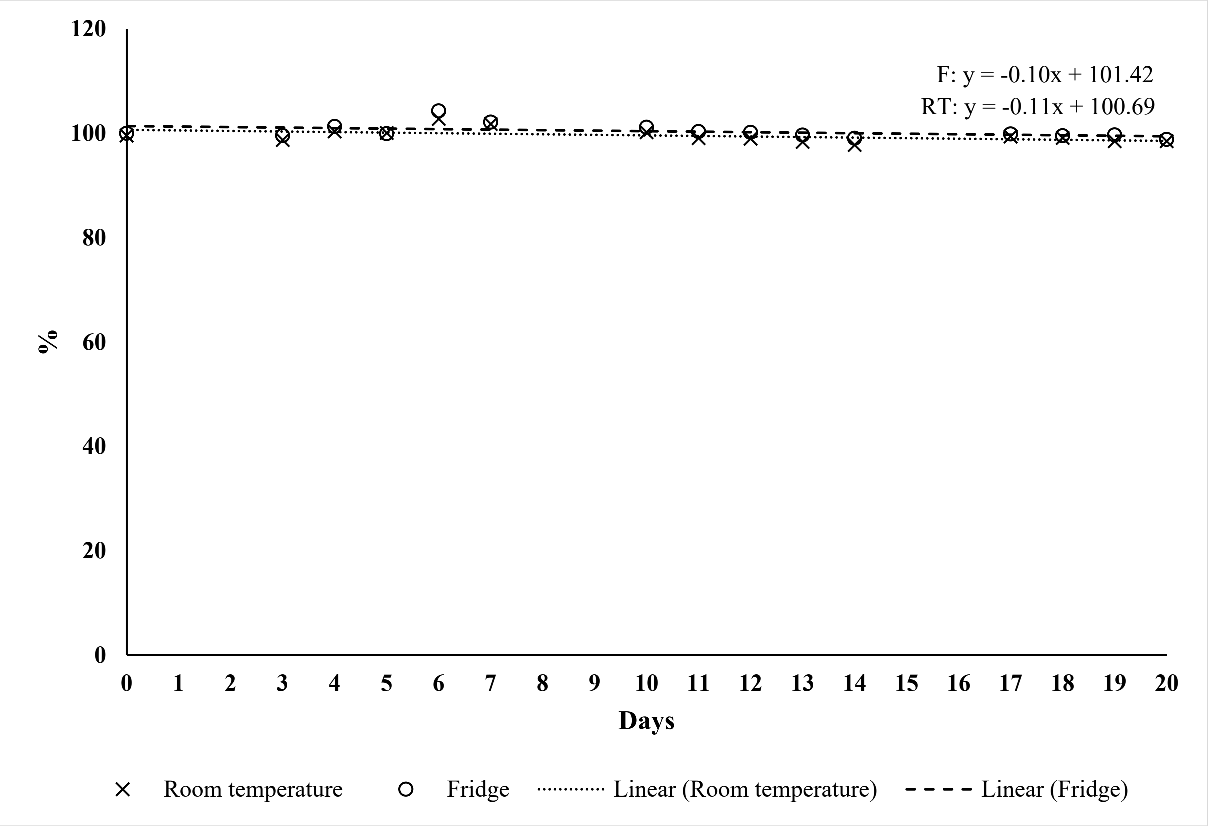

Supplementary Figure S10: Interday stability of lead structure **2** in 1:1 DMSO and 10 mM HEPES-NaOH pH 7.5 with 150 mM NaCl.

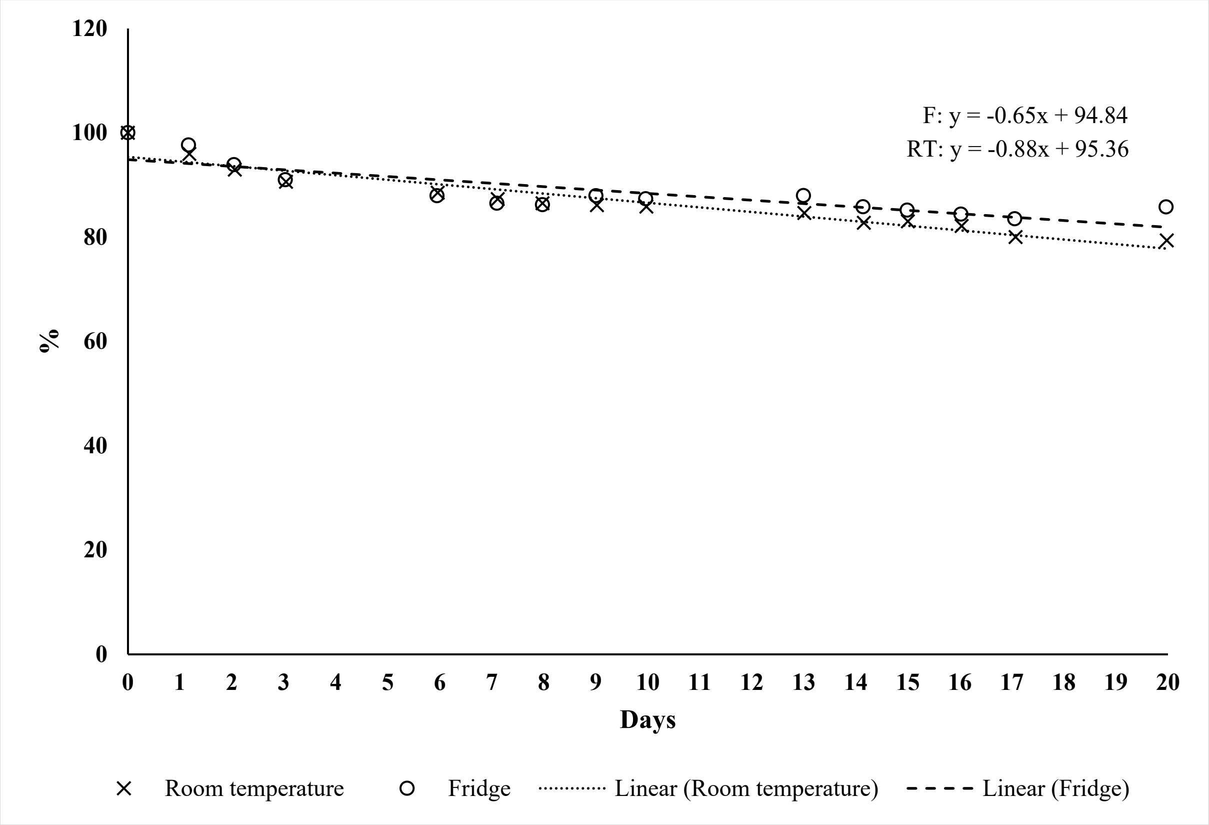

Supplementary Figure S11: Interday stability of **1a** in 1:1 DMSO and 10 mM HEPES-NaOH pH 7.5 with 150 mM NaCl.

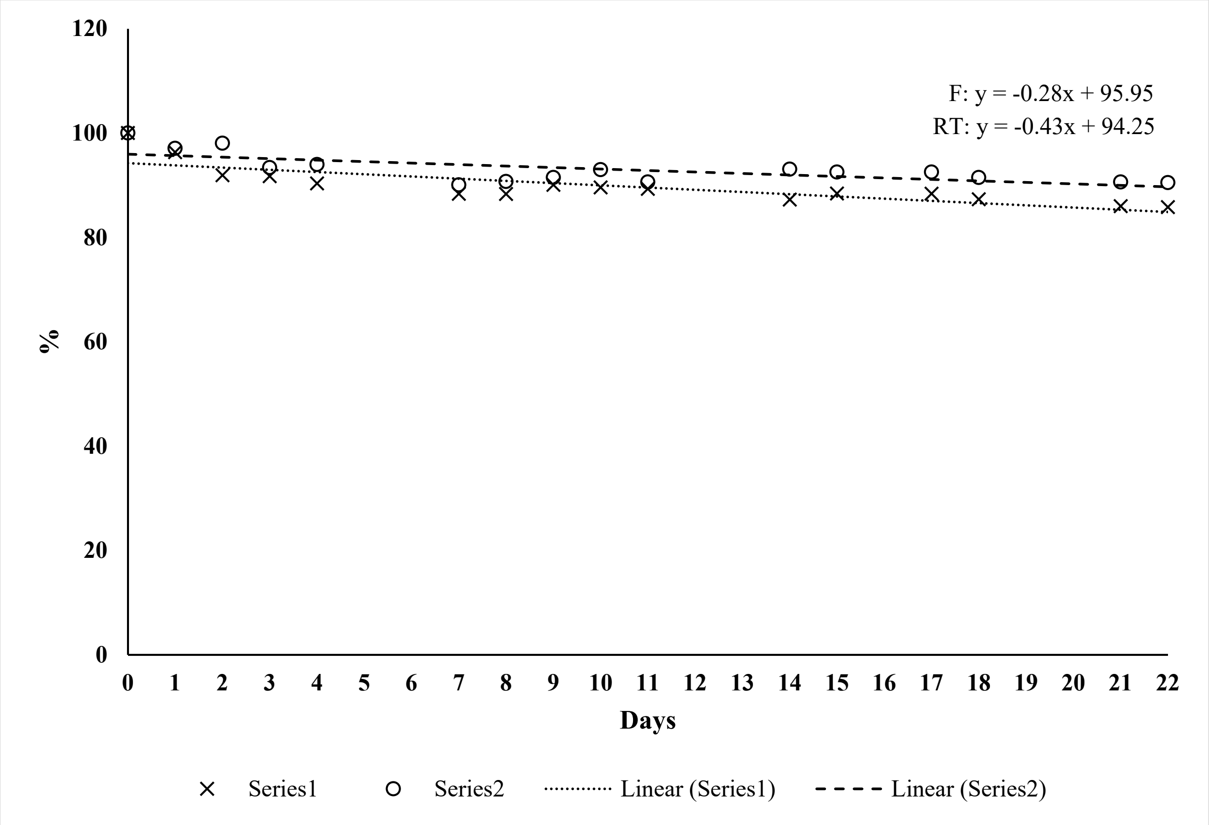

Supplementary Figure S12: Interday stability of **1b** in 1:1 DMSO and 10 mM HEPES-NaOH pH 7.5 with 150 mM NaCl.

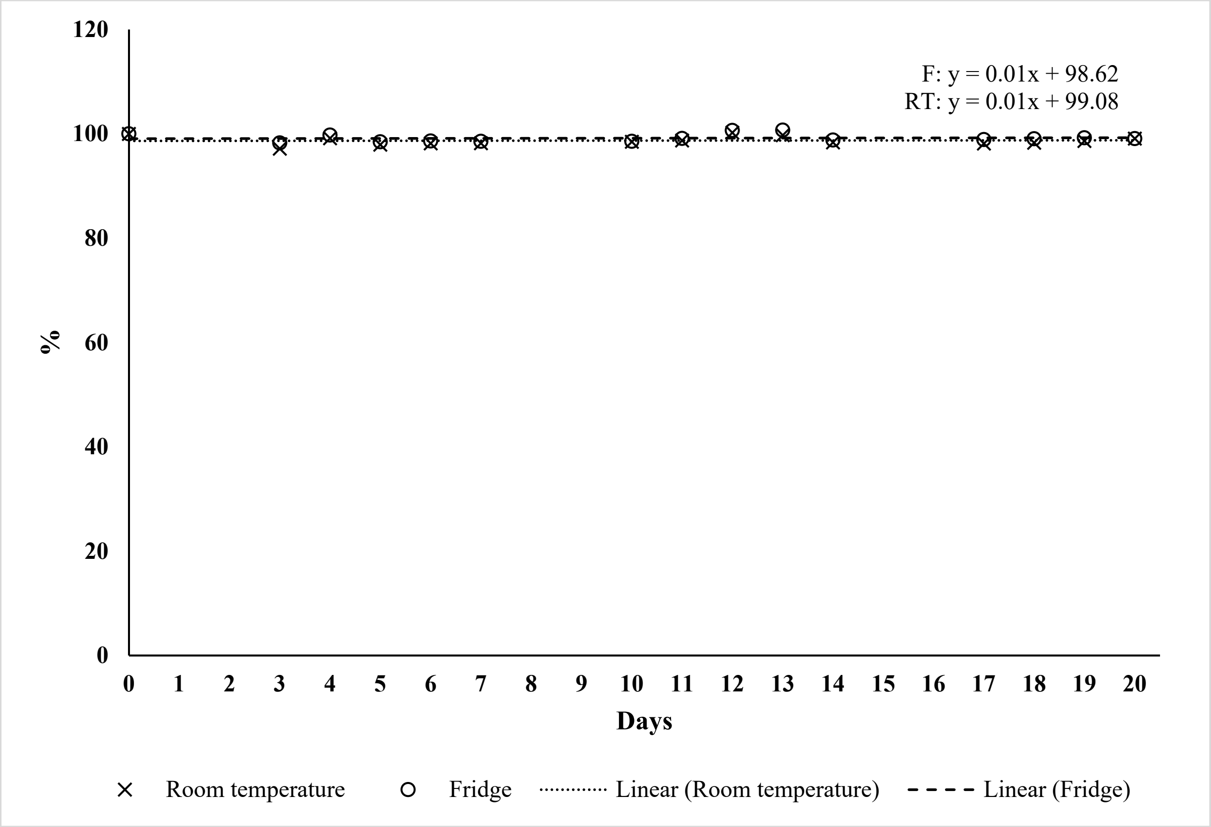

Supplementary Figure S13: Interday stability of **2a** in 1:1 DMSO and 10 mM HEPES-NaOH pH 7.5 with 150 mM NaCl.

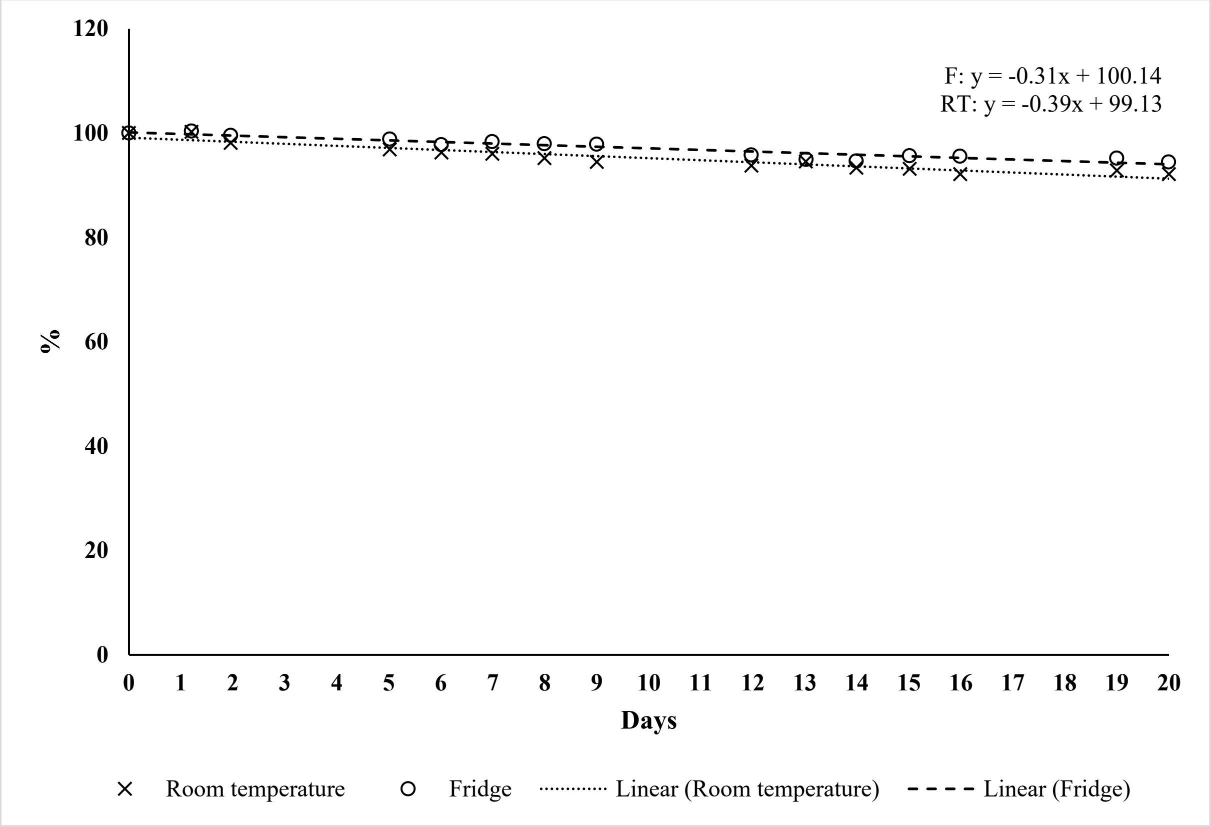

Supplementary Figure S14: Interday stability of **2b** in 1:1 DMSO and 10 mM HEPES-NaOH pH 7.5 with 150 mM NaCl.

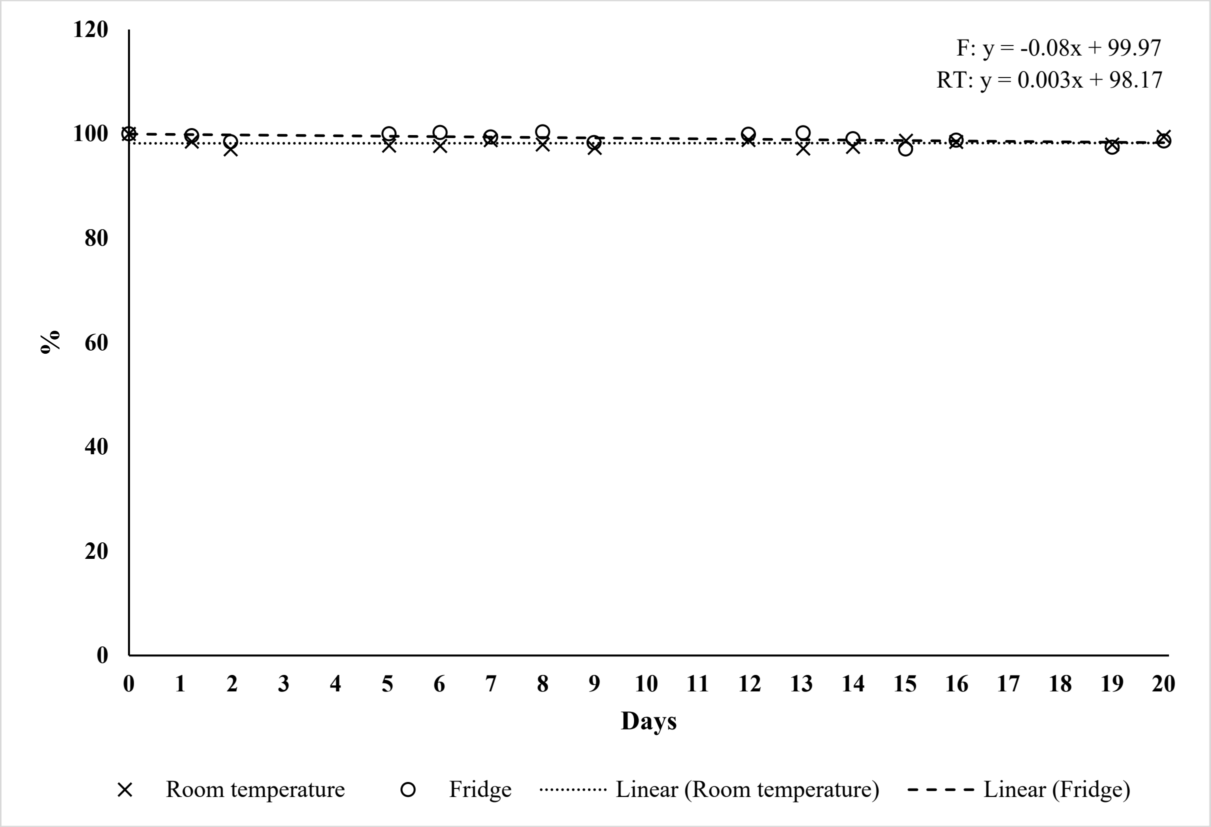

Supplementary Figure S15: Interday stability of **2c** in 1:1 DMSO and 10 mM HEPES-NaOH pH 7.5 with 150 mM NaCl.

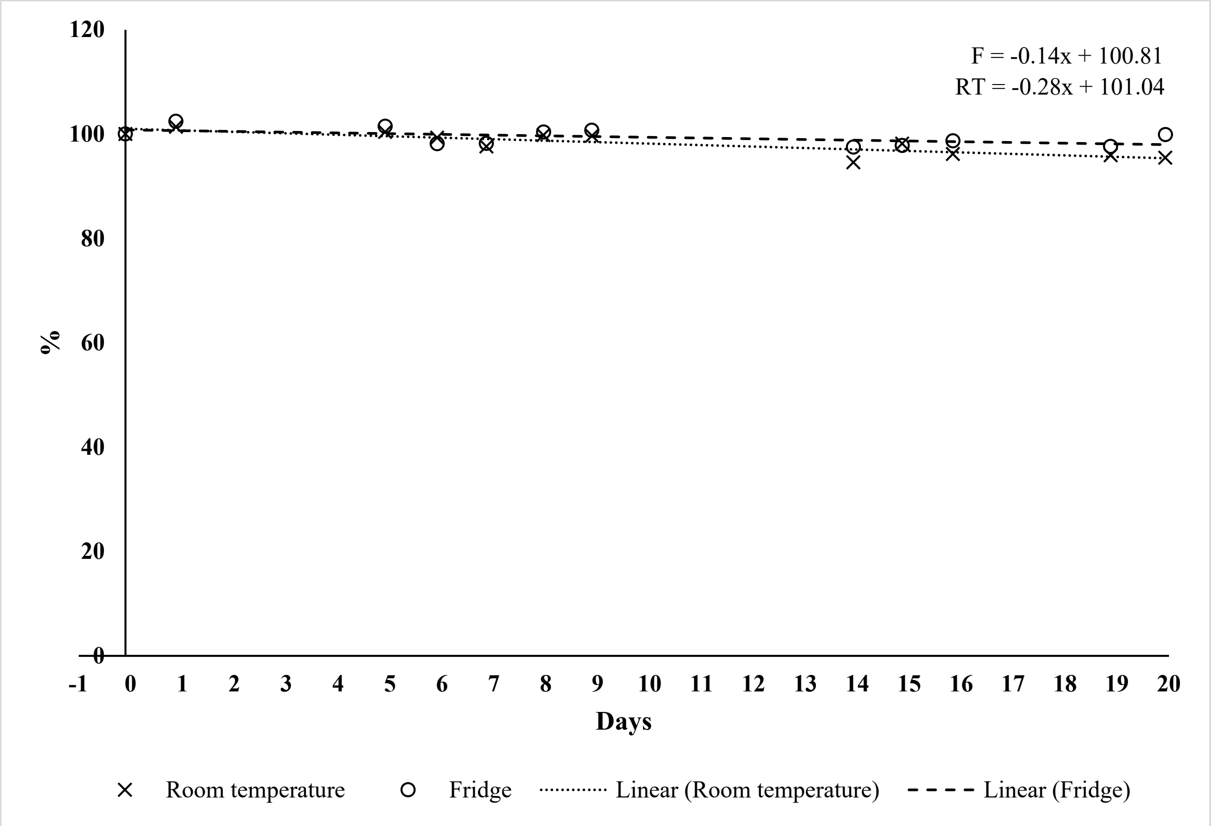

Supplementary Figure S16: Interday stability of **2d** in 1:1 DMSO and 10 mM HEPES-NaOH pH 7.5 with 150 mM NaCl.

HTRF assay binding curves

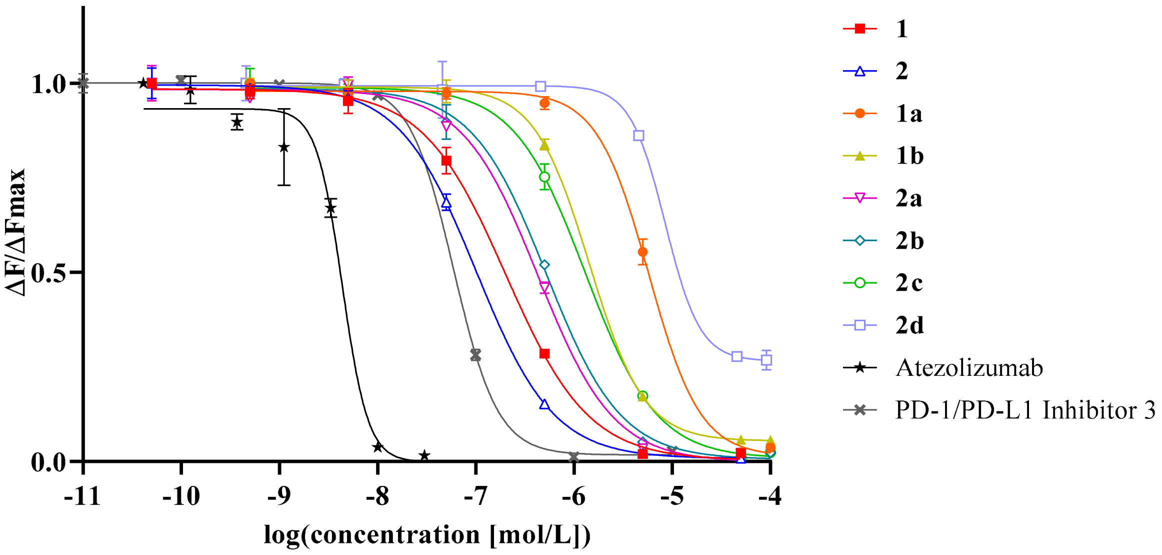

Supplementary Figure S17: Representative, normalized binding affinity curves of lead structures **1**, **2**, reference antibody atezolizumab, reference peptide PD-1/PD-L1 Inhibitor 3 as well as the six synthesized compounds using the HTRF assay. No full dose response curves were observed for **2d**.

Spearman's rank correlation coefficient

Supplementary Table S2: Spearman's rank correlation coefficient of physico-chemical properties, ligand docking parameters and measured HTRF IC_{50} values. *$p < 0.05$

Spearman's ρ	clog*P*	tPSA [Å²]	log*D*	µHPLC $\log P_{OW}^{pH\ 7.4}$	Binding Affinity Score	Affinity [kcal/mol]	IC_{50}
**clog*P***	1						
**tPSA [Å²]**	-0.51	1					
**log*D***	0.95*	-0.65	1				
**µHPLC $\log P_{OW}^{pH\ 7.4}$**	0.95*	-0.65	1	1			
**Binding Affinity Score**	-0.74*	0.36	-0.60	-0.60	1		
**Affinity [kcal/mol]**	-0.71*	0.01	-0.50	-0.50	0.79*	1	
**IC_{50}**	0.29	0.16	0.31	0.31	-0.52	-0.64	1

Small-scale radiosynthesis

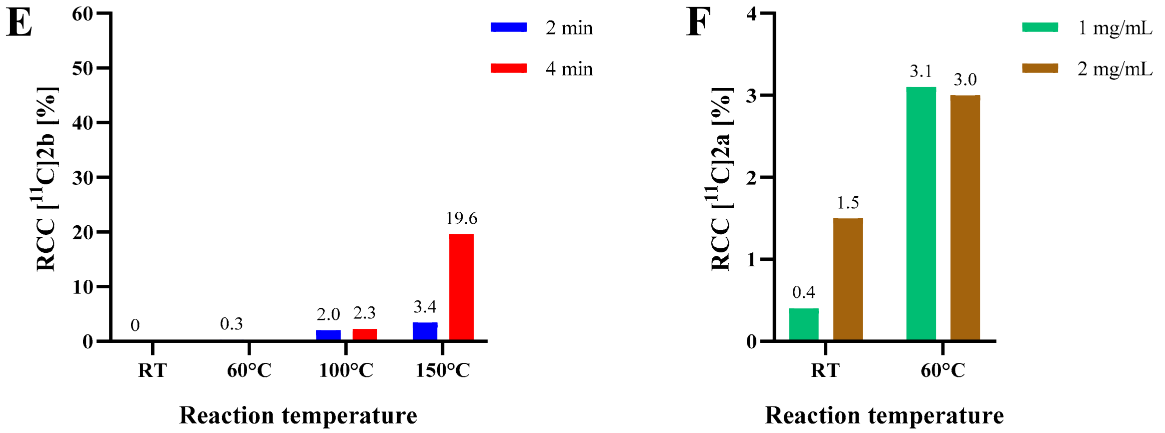

Supplementary Figure S18: Radiochemical conversion (RCC) of (E) **[^{11}C]2b** at various reaction conditions using [^{11}C]CH_3I and 2 mg/mL precursor without base. (F) Influence of precursor concentration for **[^{11}C]2a** at various temperatures using [^{11}C]CH_3I as methylating agent.

Substance purity

Supplementary Figure S199: Methyl (*S*)-1-(2,6-dimethoxy-4-((2-methyl-[1,1'-biphenyl]-3-yl)methoxy)benzyl)piperidine-2-carboxylate (**1a**):

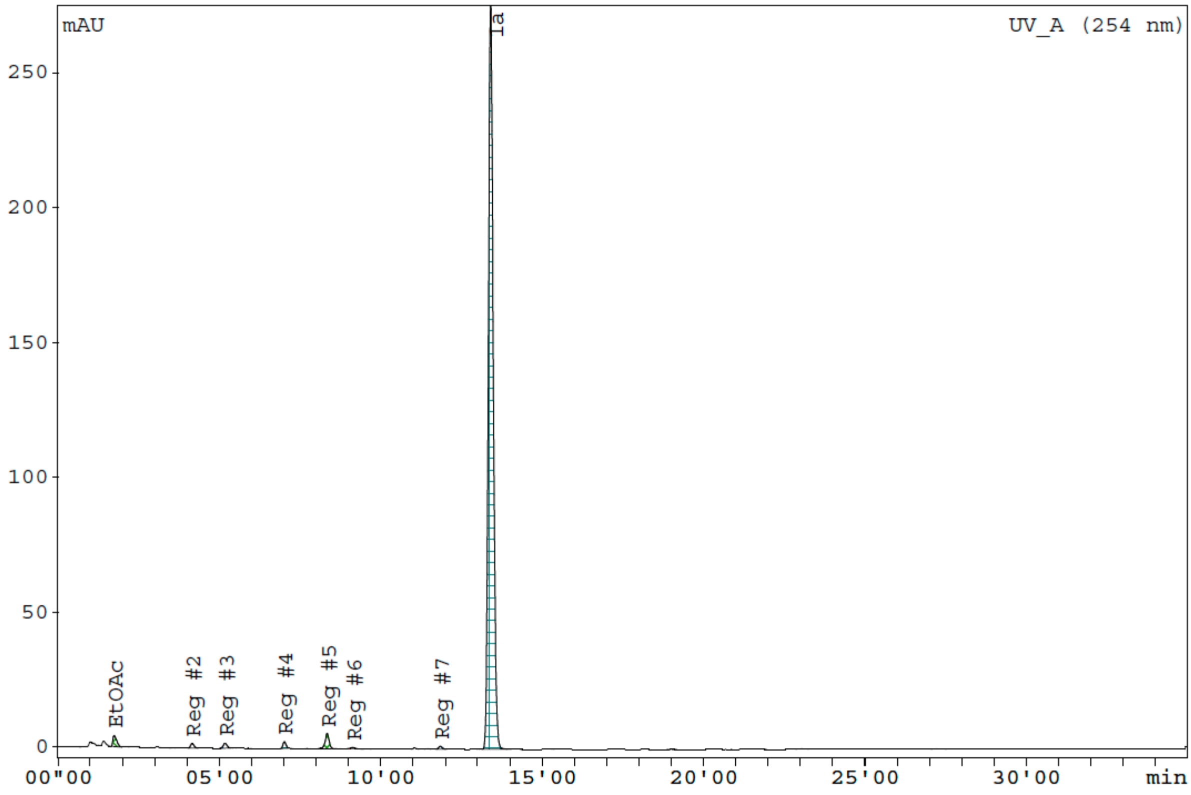

Integration UV_A (254 nm)

Substance	R/T<br>s	Type	Area<br>mAU*s	%Area<br>%
EtOAc	01'45	BB	33,874	1,11
Reg #2	04'10	BB	11,923	0,39
Reg #3	05'11	BB	14,509	0,48
Reg #4	07'01	BB	16,781	0,55
Reg #5	08'21	BB	49,552	1,63
Reg #6	09'08	BB	4,420	0,15
Reg #7	11'51	BB	9,271	0,30
1a	13'24	BB	2903,024	95,39
Sum in ROI			3043,355	100,00

Supplementary Figure S20: 2-Fluoroethyl (*S*)-1-(2,6-dimethoxy-4-((2-methyl-[1,1'-biphenyl]-3-yl)methoxy)benzyl)piperidine-2-carboxylate (**1b**):

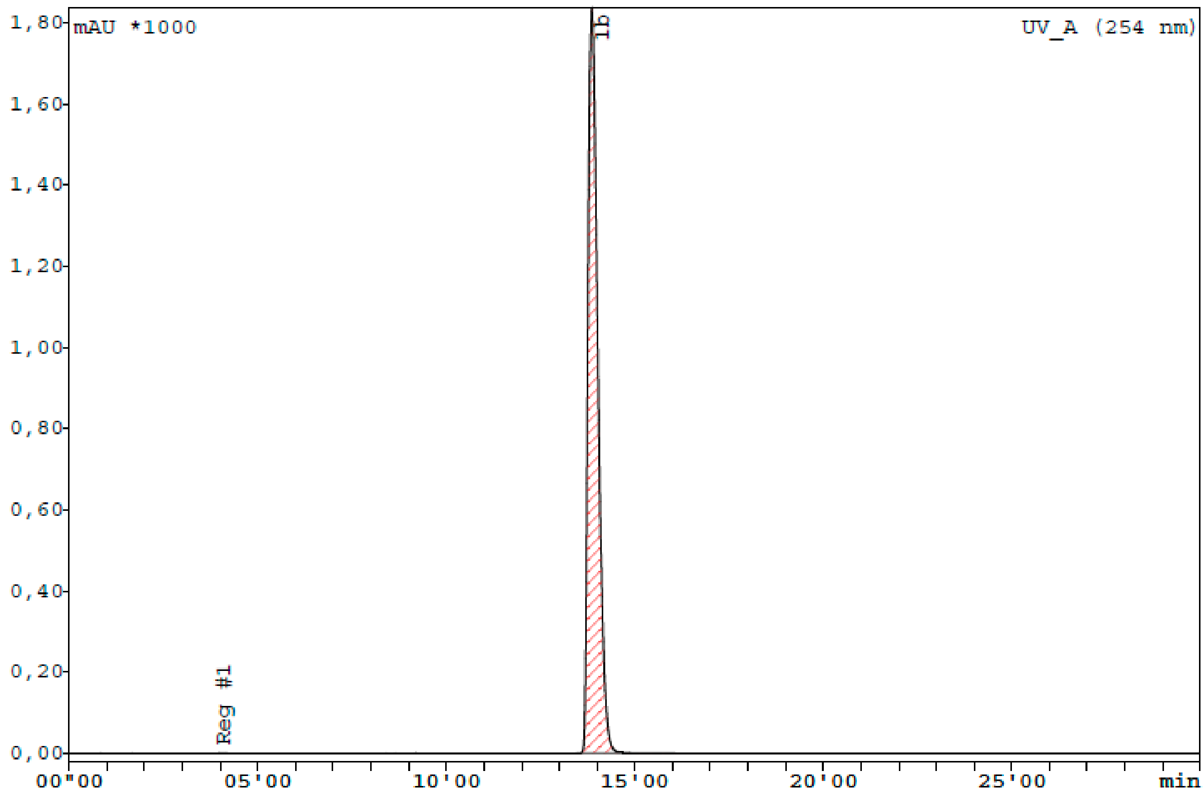

Integration UV_A (254 nm)

Substance	R/T s	Type	Area mAU*s	%Area %
Reg #1	04'04	BB	6,55	0,02
1b	13'52	BB	33301,01	99,98
Sum in ROI			33307,56	100,00

Supplementary Figure S21: *N*-(2-(((2-methoxy-6-((2-methyl-[1,1'-biphenyl]-3-yl)methoxy)pyridin-3-yl)methyl)(methyl)amino)ethyl)acetamide (**2a**):

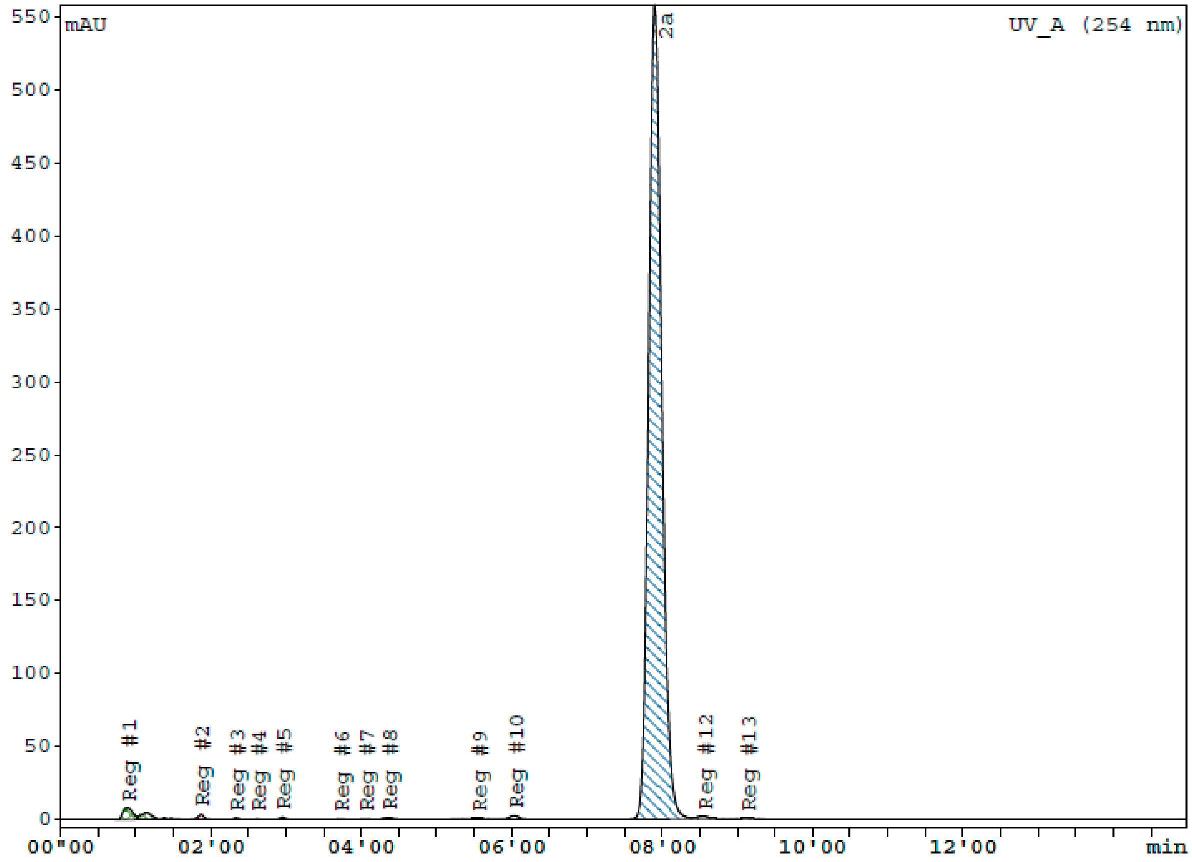

Integration UV_A (254 nm)

Substance	R/T s	Type	Area mAU*s	%Area %
Reg #1	53"00	BB	124,886	1,79
Reg #2	01'52	BB	16,005	0,23
Reg #3	02'20	BB	3,259	0,05
Reg #4	02'37	BB	0,879	0,01
Reg #5	02'57	BB	6,563	0,09
Reg #6	03'43	BD	1,717	0,02
Reg #7	04'03	DD	1,991	0,03
Reg #8	04'21	DB	10,599	0,15
Reg #9	05'33	BD	14,728	0,21
Reg #10	06'02	DB	21,166	0,30
2a	07'54	BD	6755,576	96,68
Reg #12	08'33	DB	24,844	0,36
Reg #13	09'08	BB	5,392	0,08
Sum in ROI			6987,605	100,00

Supplementary Figure S22: *N*-(2-(((2-methoxy-6-((2-methyl-[1,1'-biphenyl]-3-yl)methoxy)pyridin-3-yl)methyl)amino)ethyl)-*N*-methylacetamide (**2b**):

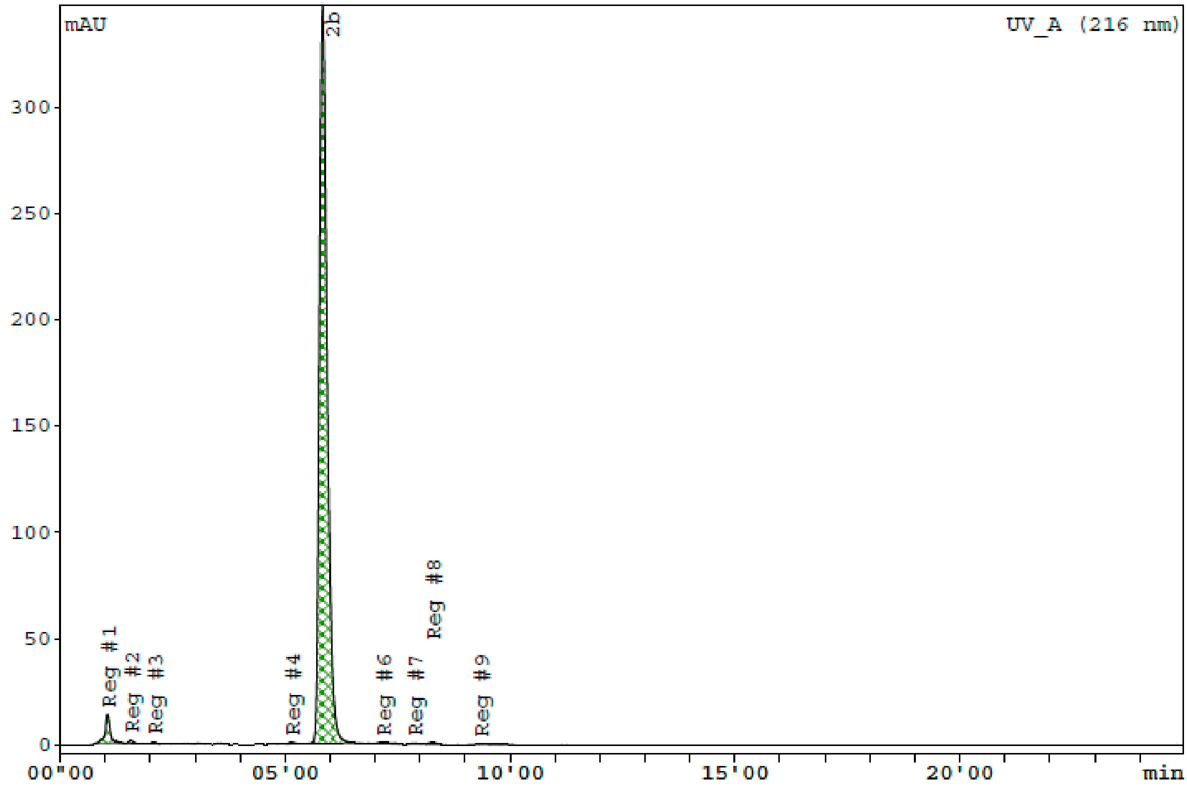

Integration UV_A (216 nm)

Substance	R/T<br>s	Type	Area<br>mAU*s	%Area<br>%
Reg #1	01'03	DB	113,503	2,59
Reg #2	01'34	BB	9,495	0,22
Reg #3	02'04	BB	4,213	0,10
Reg #4	05'09	BB	6,576	0,15
2b	05'50	BB	4205,578	96,02
Reg #6	07'09	BB	13,513	0,31
Reg #7	07'52	BB	5,537	0,13
Reg #8	08'16	BB	9,982	0,23
Reg #9	09'21	BB	11,350	0,26
Sum in ROI			4379,747	100,00

Supplementary Figure S23: *N*-(2-(((2-methoxy-6-((2-methyl-[1,1'-biphenyl]-3-yl)methoxy)pyridin-3-yl)methyl)(methyl)amino)ethyl)-*N*-methylacetamide (**2c**):

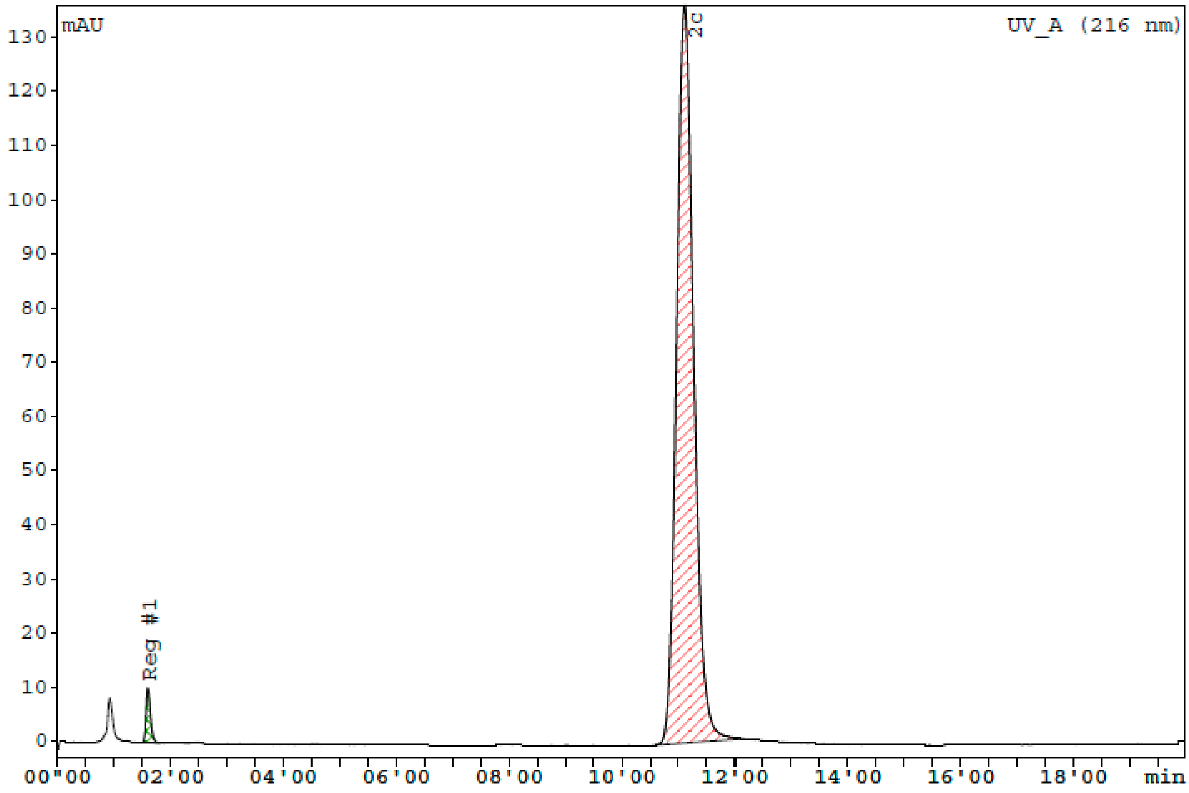

Integration UV_A (216 nm)

Substance	R/T s	Type	Area mAU*s	%Area %
Reg #1	01'37	BB	55,517	1,89
2c	11'07	BB	2882,809	98,11
Sum in ROI			2938,326	100,00

Supplementary Figure S24: 2-fluoroethyl (2-acetamidoethyl)((2-methoxy-6-((2-methyl-[1,1'-biphenyl]-3-yl)methoxy)pyridin-3-yl)methyl)carbamate (**2d**):

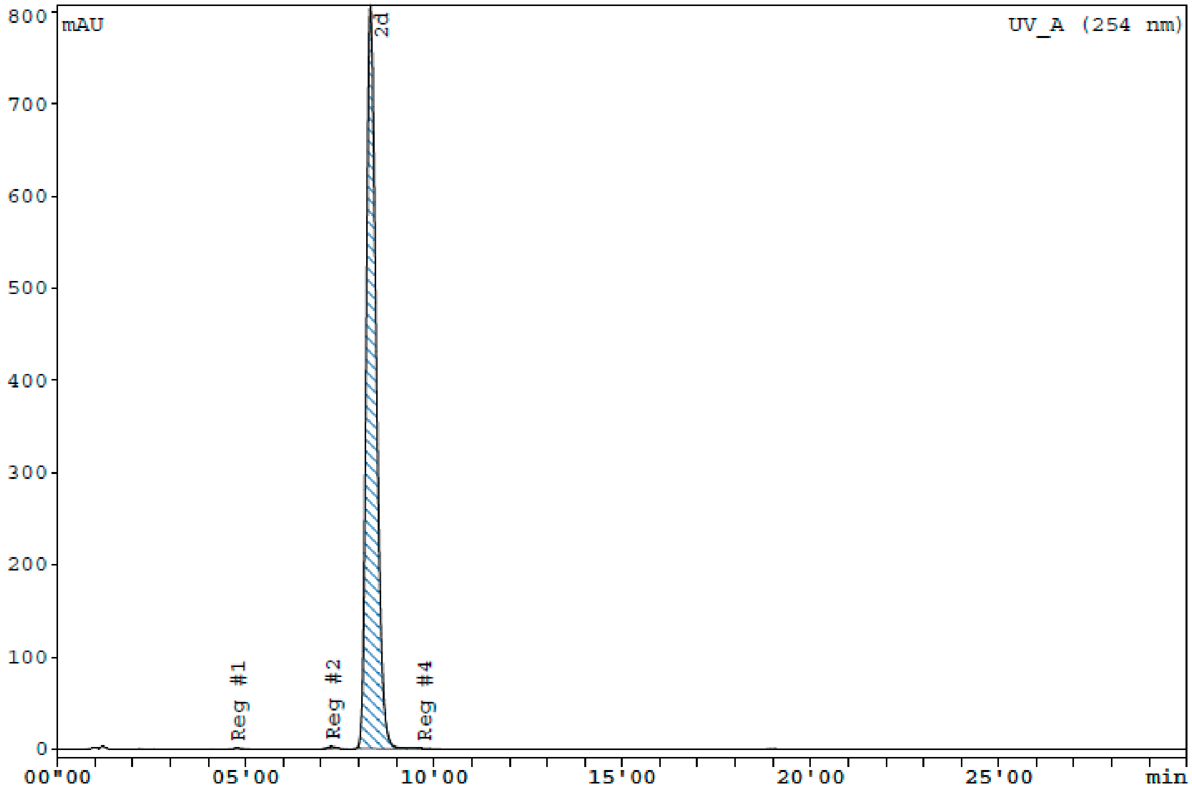

Integration UV_A (254 nm)

Substance	R/T s	Type	Area mAU*s	%Area %
Reg #1	04'46	BB	11,58	0,08
Reg #2	07'17	BB	31,76	0,21
2d	08'19	BD	14897,28	99,55
Reg #4	09'42	DB	23,59	0,16
Sum in ROI			14964,21	100,00

Nuclear magnetic resonance spectroscopy

Supplementary Figure S25: Methyl (*S*)-1-(2,6-dimethoxy-4-((2-methyl-[1,1'-biphenyl]-3-yl)methoxy)benzyl)piperidine-2-carboxylate (**1a**):

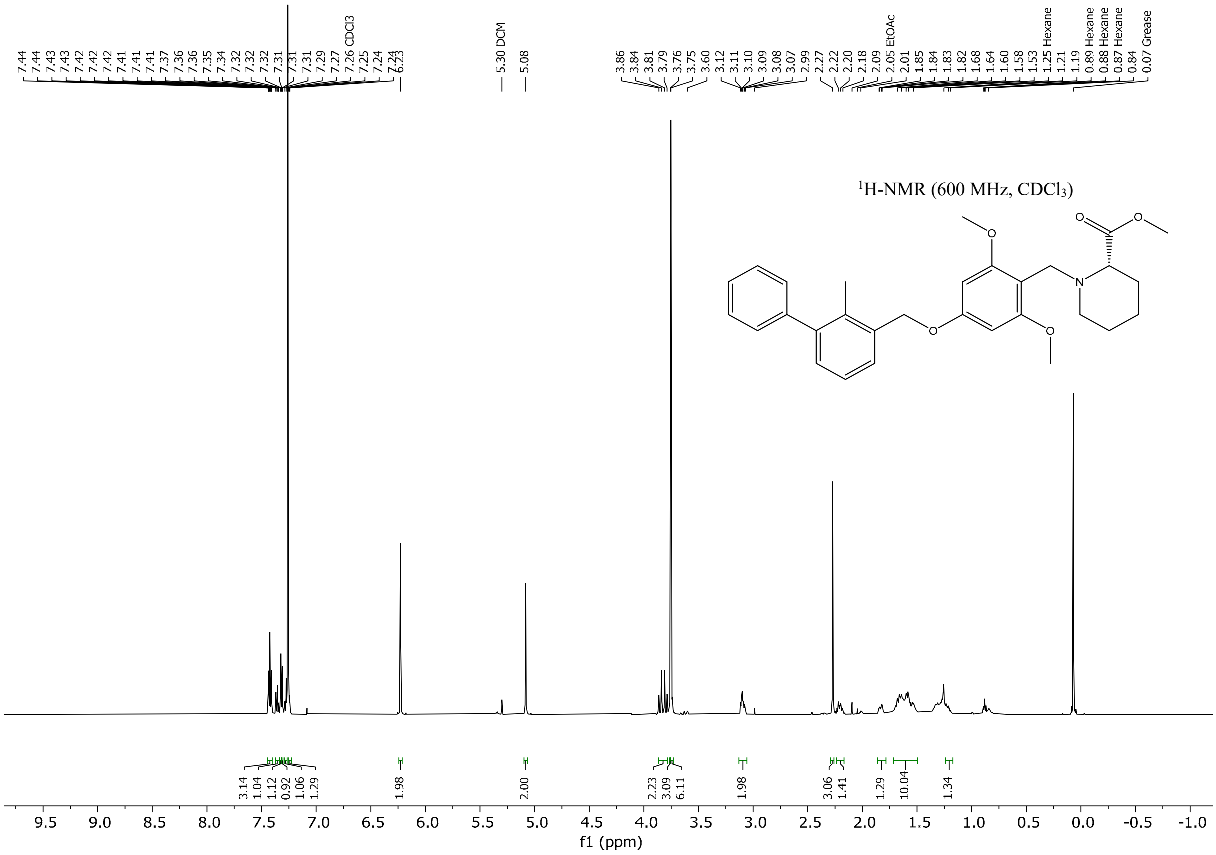

^{1}H-NMR (600 MHz, $CDCl_3$): δ 7.44 – 7.41 (m, 3H), 7.37 – 7.24 (m, 5H), 6.23 (s, 2H), 5.08 (s, 2H), 3.86 – 3.79 (m, 2H), 3.76 (s, 3H), 3.75 (s, 6H), 3.12 – 3.07 (m, 2H), 2.27 (s, 3H), 2.22 – 2.18 (m, 1H), 1.85 – 1.82 (m, 1H), 1.68 – 1.53 (m, 4H), 1.25 – 1.19 (m, 2H).

Supplementary Figure S26: Methyl (*S*)-1-(2,6-dimethoxy-4-((2-methyl-[1,1'-biphenyl]-3-yl)methoxy)benzyl)piperidine-2-carboxylate (**1a**):

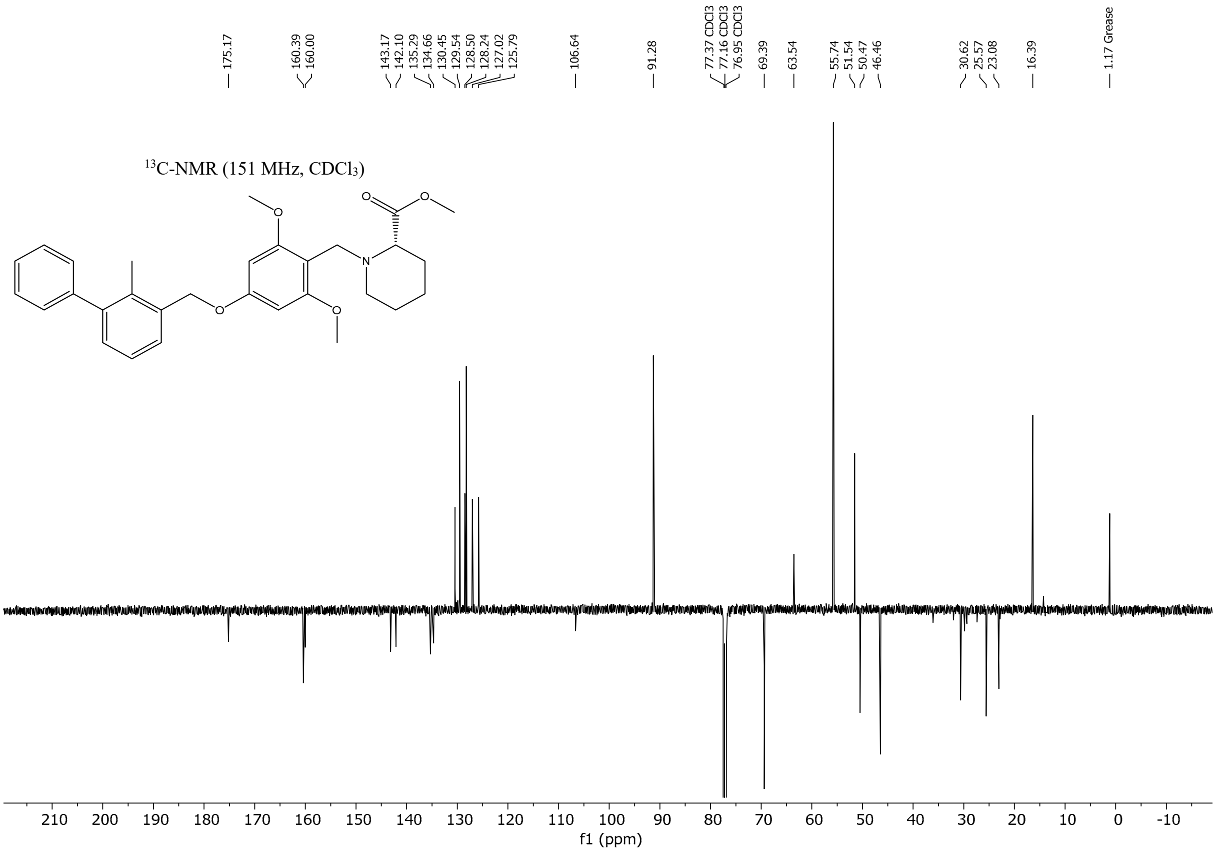

^{13}C-NMR (151 MHz, $CDCl_3$): δ 175.17, 160.39, 160.00, 143.17, 142.10, 135.29, 134.66, 130.45, 129.54, 128.50, 128.24, 127.02, 125.79, 106.64, 91.28, 69.39, 63.54, 55.74, 51.54, 50.47, 46.46, 30.62, 25.57, 23.08, 16.39.

Supplementary Figure S27: Methyl (*S*)-1-(2,6-dimethoxy-4-((2-methyl-[1,1'-biphenyl]-3-yl)methoxy)benzyl)piperidine-2-carboxylate (**1a**):

^{1}H-^{1}H COSY NMR (600 MHz, $CDCl_3$)

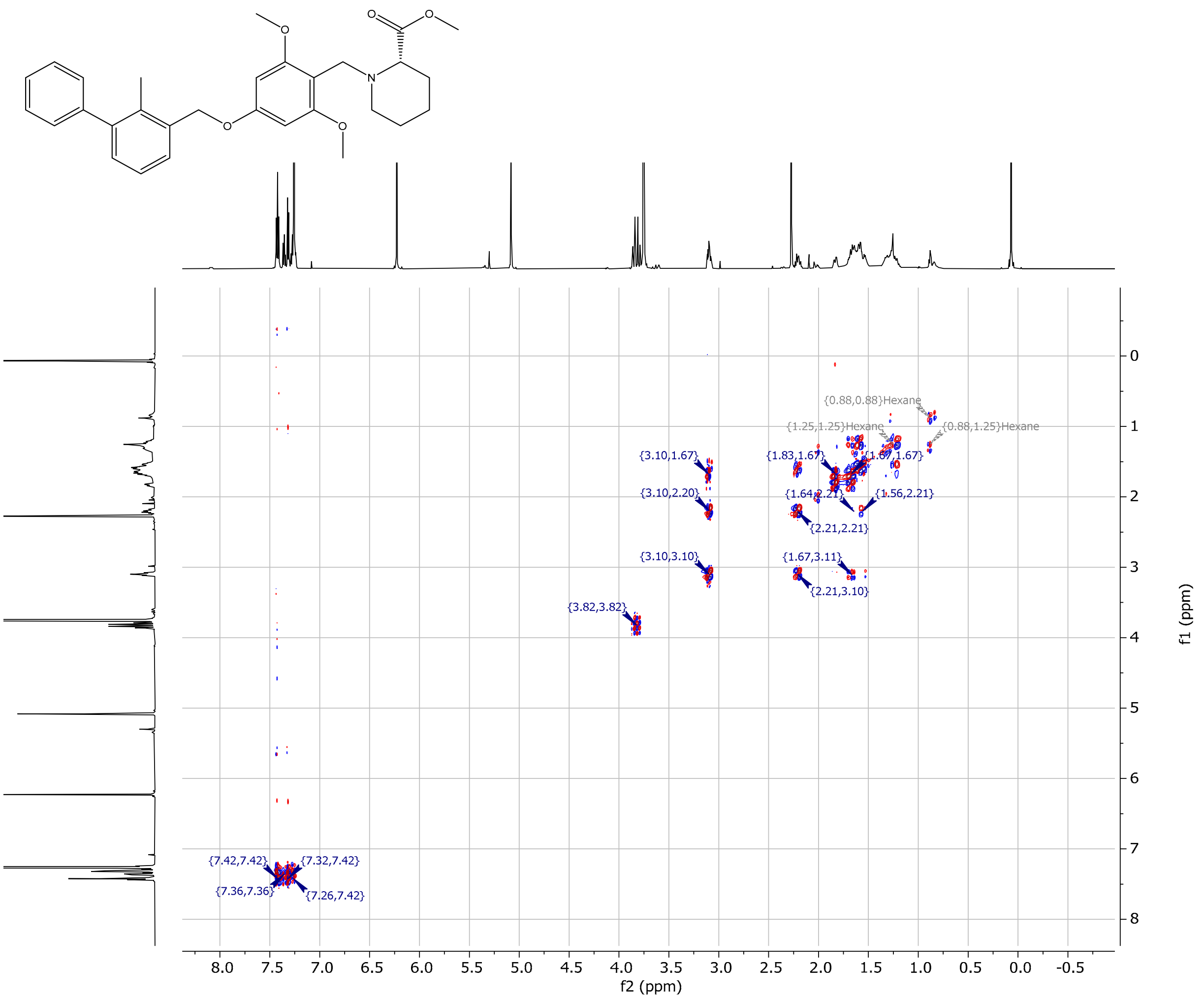

^{1}H-NMR (600 MHz, $CDCl_3$): δ 7.42, 7.42, 7.42, 7.36, 7.36, 7.32, 7.26, 7.26, 3.82, 3.11, 3.10, 3.10, 2.21, 2.21, 2.21, 2.20, 1.83, 1.83, 1.67, 1.67, 1.67, 1.64, 1.59, 1.59, 1.56, 1.22, 1.21, 1.21.

^{1}H-NMR (600 MHz, $CDCl_3$): δ 7.42, 7.42, 7.42, 7.36, 7.32, 7.32, 7.26, 7.26, 3.82, 3.10, 3.10, 3.10, 2.21, 2.21, 2.21, 2.21, 1.83, 1.83, 1.67, 1.67, 1.67, 1.67, 1.64, 1.59, 1.59, 1.56, 1.21, 1.21.

Supplementary Figure S28: Methyl (*S*)-1-(2,6-dimethoxy-4-((2-methyl-[1,1'-biphenyl]-3-yl)methoxy)benzyl)piperidine-2-carboxylate (**1a**):

^{1}H-^{13}C HSQC NMR (600 MHz, 151 MHz, $CDCl_3$)

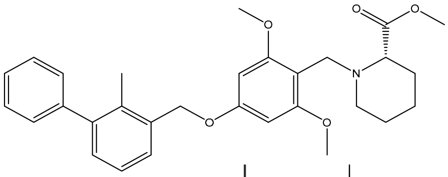

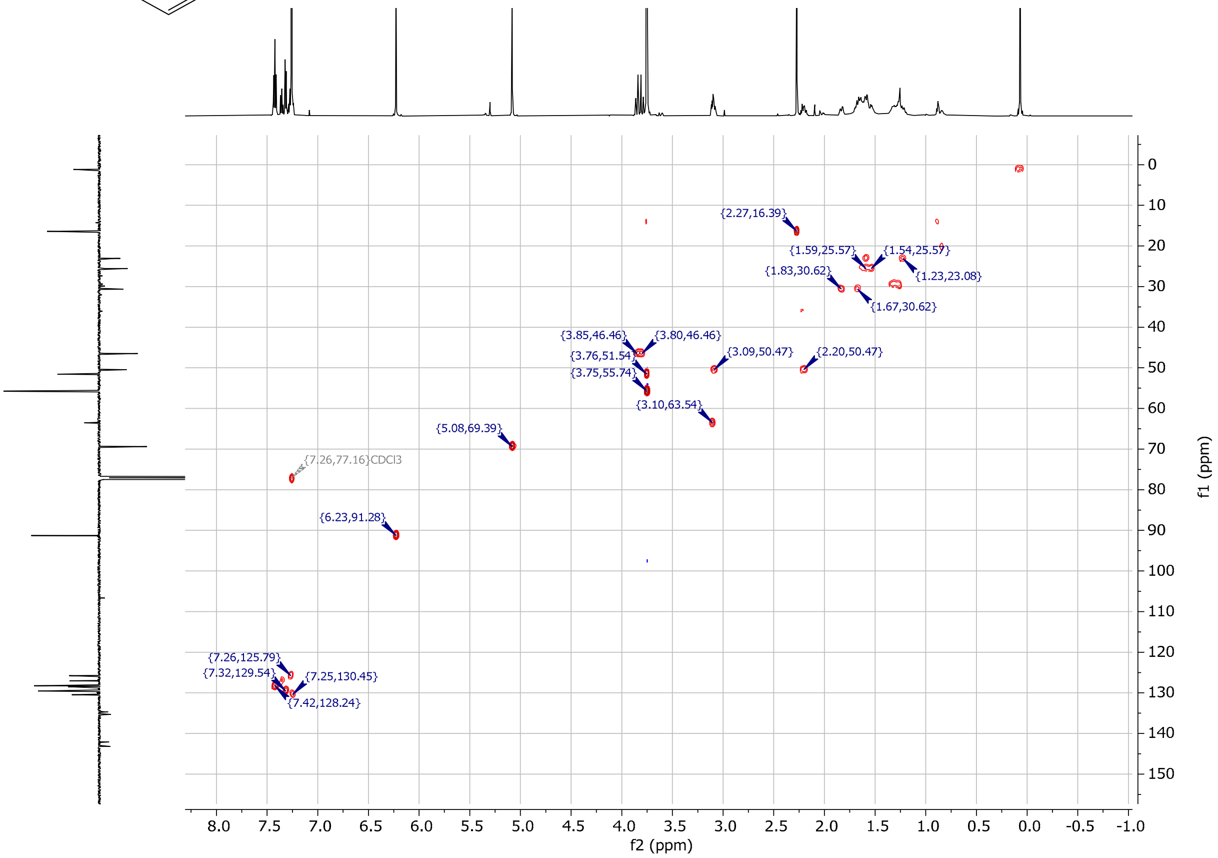

^{13}C-NMR (151 MHz, $CDCl_3$): δ 130.45, 129.54, 128.24, 127.02, 125.79, 91.28, 69.39, 63.54, 55.74, 51.54, 50.47, 50.47, 46.46, 46.46, 30.62, 30.62, 25.57, 25.57, 23.08, 23.08, 16.39

^{1}H-NMR (600 MHz, $CDCl_3$): δ 7.42, 7.36, 7.32, 7.26, 7.25, 6.23, 5.08, 3.85, 3.80, 3.76, 3.75, 3.10, 3.09, 2.27, 2.20, 1.83, 1.67, 1.59, 1.59, 1.54, 1.23.

Supplementary Figure S29: 2-Fluoroethyl (*S*)-1-(2,6-dimethoxy-4-((2-methyl-[1,1'-biphenyl]-3-yl)methoxy)benzyl)piperidine-2-carboxylate (**1b**):

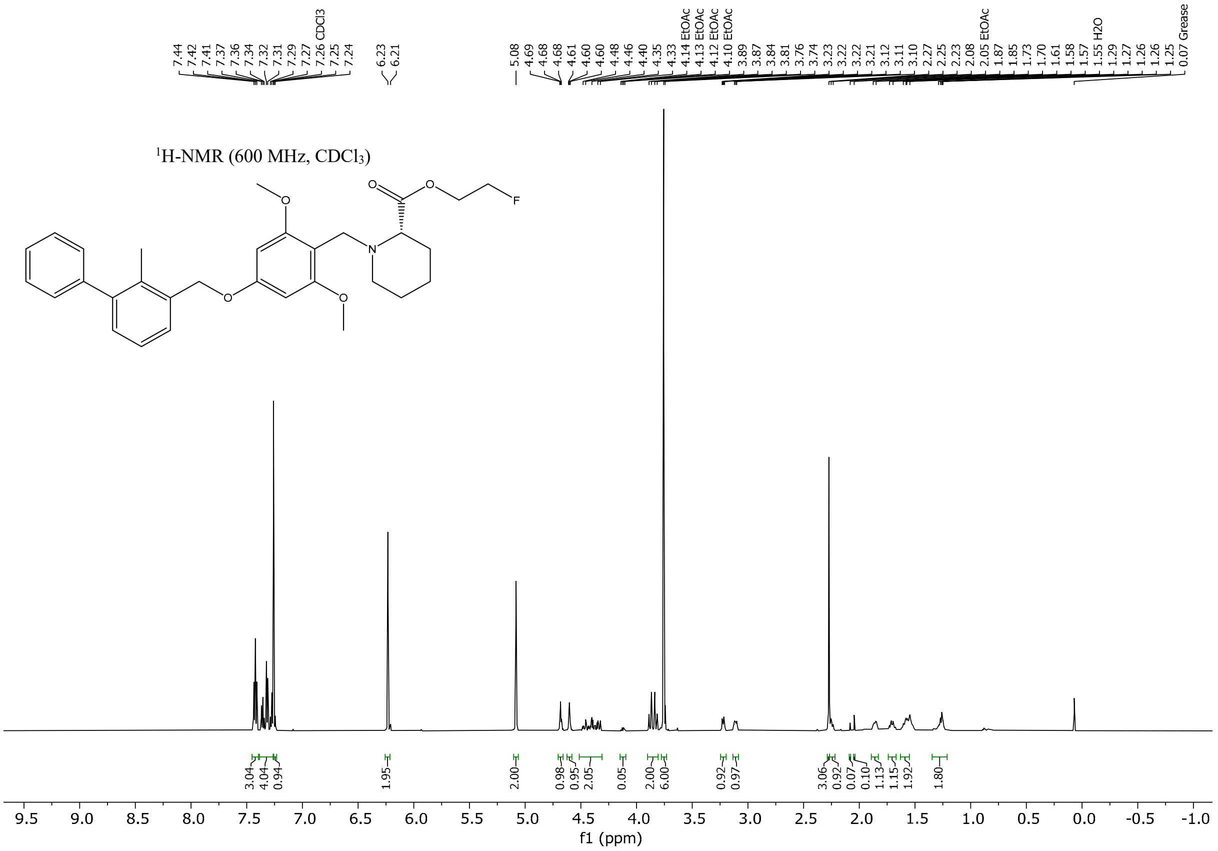

^{1}H-NMR (600 MHz, $CDCl_3$): δ 7.44 – 7.41 (m, 3H), 7.37 – 7.24 (m, 5H), 6.23 (s, 2H), 5.08 (s, 2H), 4.69 – 4.60 (m, *J* = 47.4 Hz, 2H), 4.48 – 4.33 (m, 2H), 3.89 – 3.81 (m, 2H), 3.76 (s, 6H), 3.22 (dd, *J* = 4.4 Hz, *J* = 3.9 Hz, 1H), 3.12 – 3.10 (m, 1H), 2.27 (s, 3H), 2.25 – 2.23 (m, 1H), 1.87 – 1.85 (m, 1H), 1.73 – 1.70 (m, 1H), 1.61 – 1.57 (m, 2H), 1.29 – 1.25 (m, 2H).

Supplementary Figure S30: 2-Fluoroethyl (*S*)-1-(2,6-dimethoxy-4-((2-methyl-[1,1'-biphenyl]-3-yl)methoxy)benzyl)piperidine-2-carboxylate (**1b**):

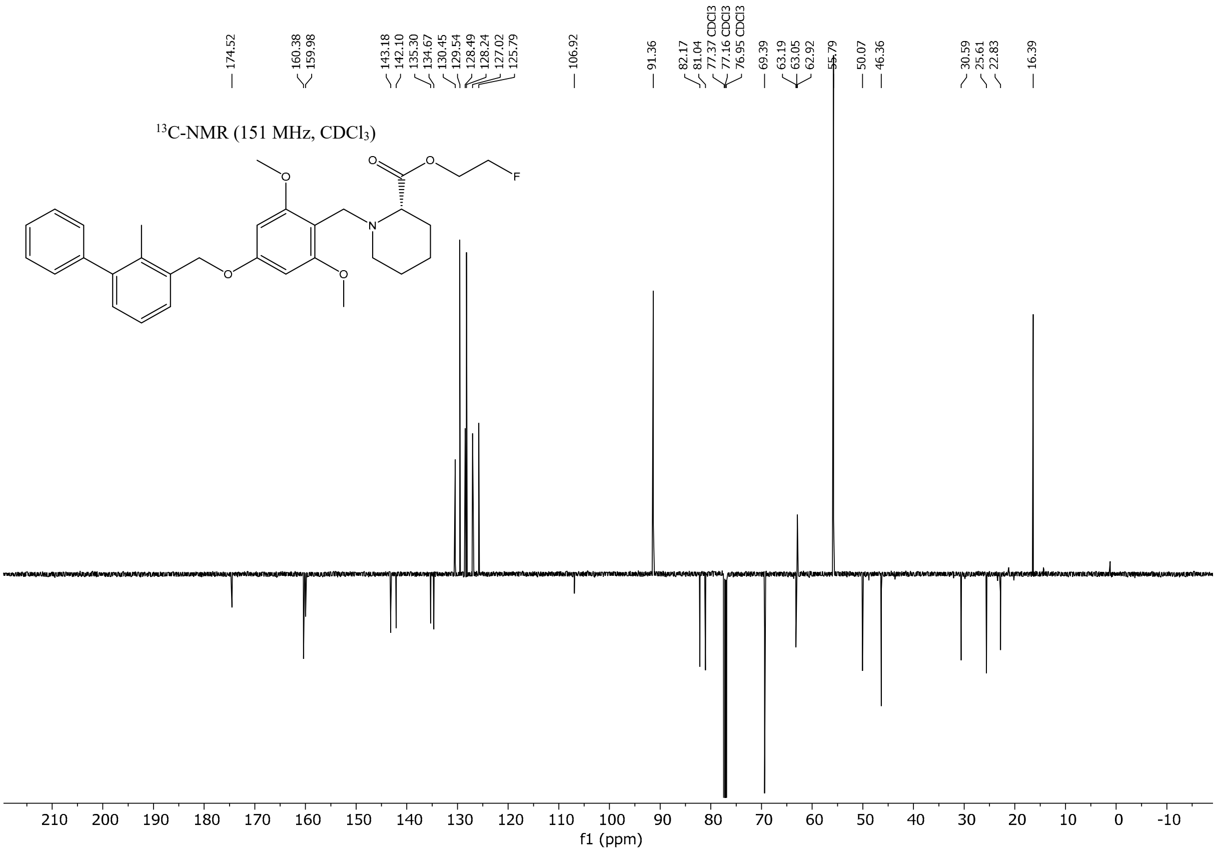

^{13}C-NMR (151 MHz, $CDCl_3$): δ 174.52, 160.38, 159.89, 143.18, 142.10, 135.30, 134.67, 130.45, 129.54, 128.49, 128.24, 127.02, 125.79, 106.92, 91.36, 81.61 (d, *J* = 170 Hz), 69.39, 63.12 (d, *J* = 20 Hz), 62.92, 55.81, 55.79, 50.07, 46.36, 30.59, 25.61, 22.83, 16.39.

Supplementary Figure S31: 2-Fluoroethyl (*S*)-1-(2,6-dimethoxy-4-((2-methyl-[1,1'-biphenyl]-3-yl)methoxy)benzyl)piperidine-2-carboxylate (**1b**):

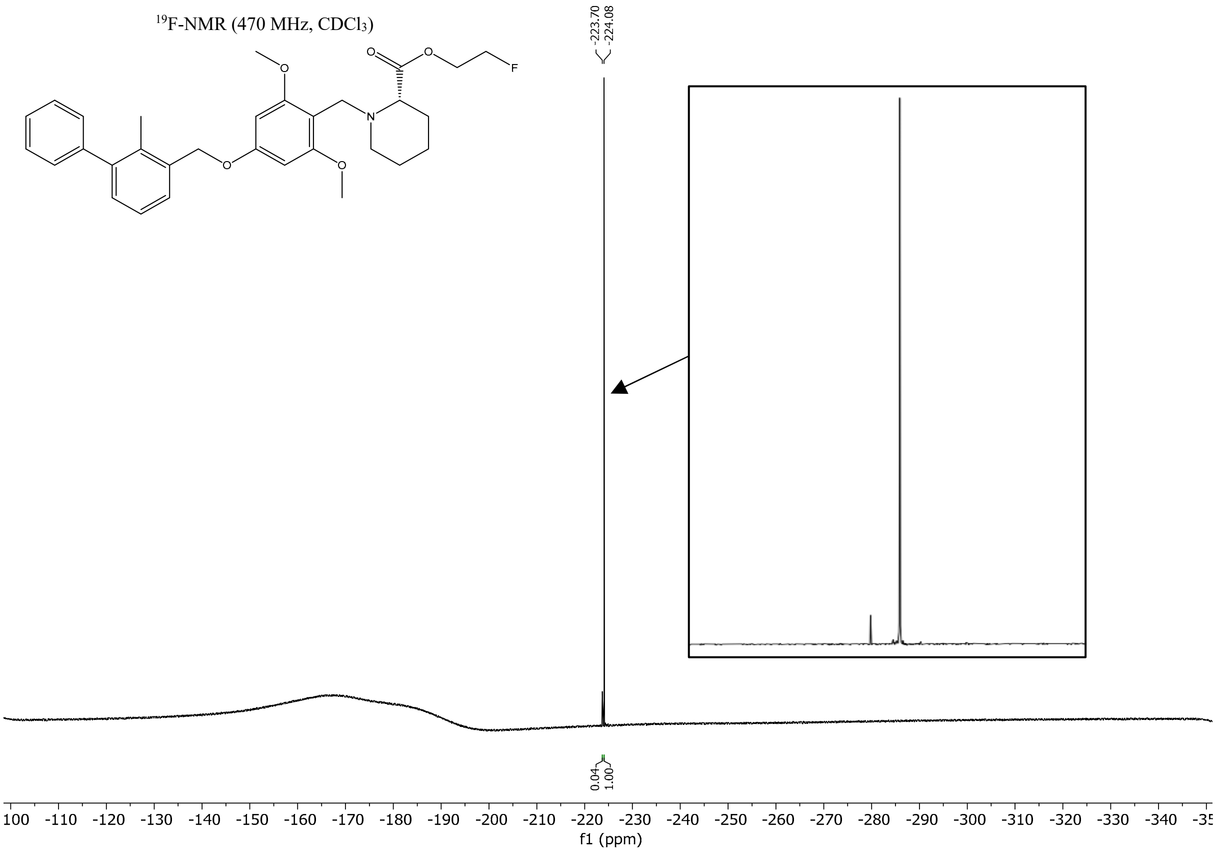

^{19}F-{^{1}H}NMR (470 MHz, $CDCl_3$): δ -224.08 (s, 1F).

Supplementary Figure S32: *N*-(2-(((2-methoxy-6-((2-methyl-[1,1'-biphenyl]-3-yl)methoxy)pyridin-3-yl)methyl)(methyl)amino)ethyl)acetamide (**2a**):

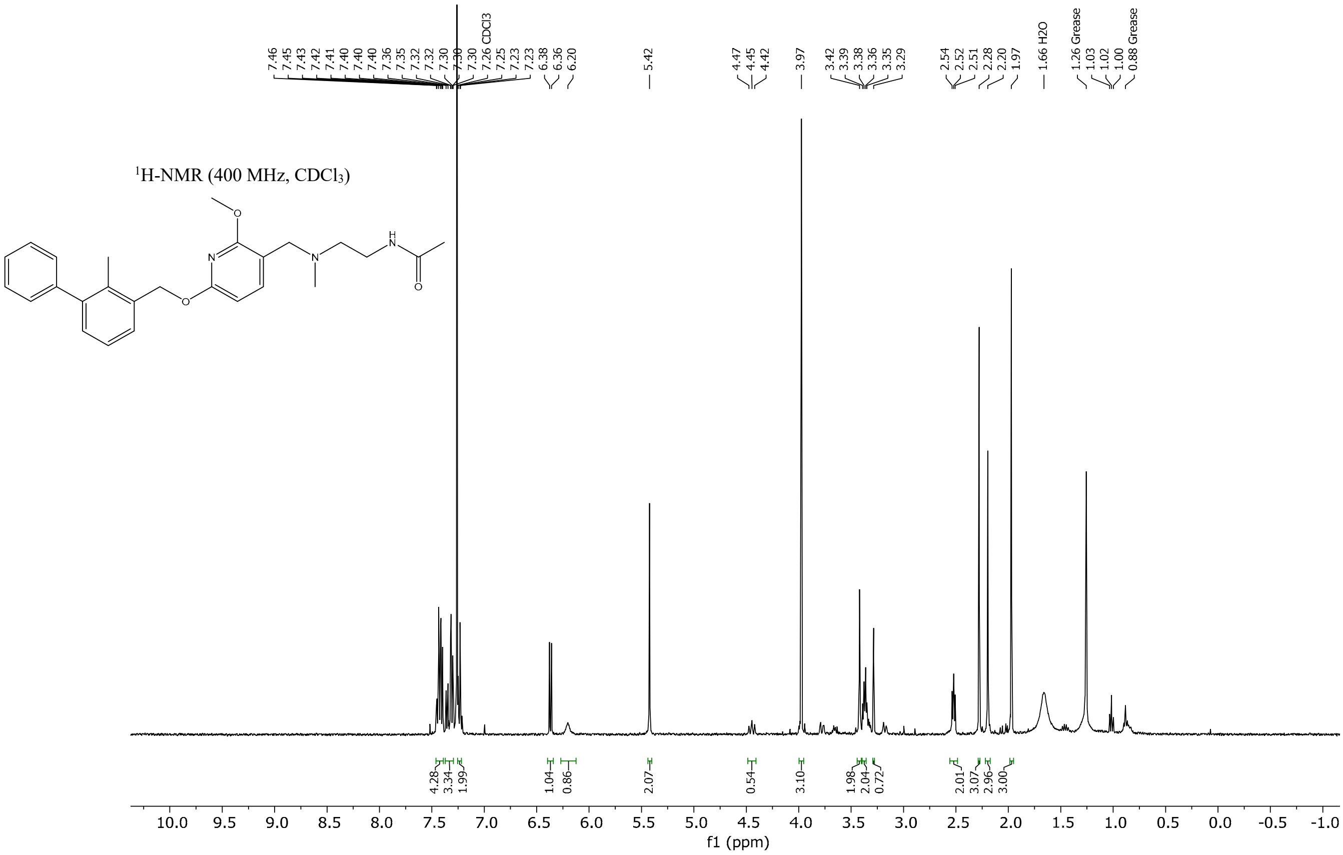

^{1}H-NMR (400 MHz, $CDCl_3$): δ 7.46 – 7.40 (m, 4H), 7.36 – 7.30 (m, 3H), 7.25 – 7.23 (m, 2H), 6.37 (d, *J* = 7.9 Hz, 1H), 6.20 (br s, 1H), 5.42 (s, 2H), 3.97 (s, 3H), 3.42 (s, 2H), 3.37 (q, *J* = 5.6 Hz, 2H), 2.52 (t, *J* = 5.6 Hz, 2H), 2.28 (s, 3H), 2.20 (s, 3H), 1.97 (s, 3H).

Supplementary Figure S33: *N*-(2-(((2-methoxy-6-((2-methyl-[1,1'-biphenyl]-3-yl)methoxy)pyridin-3-yl)methyl)amino)ethyl)-*N*-methylacetamide (**2b)**:

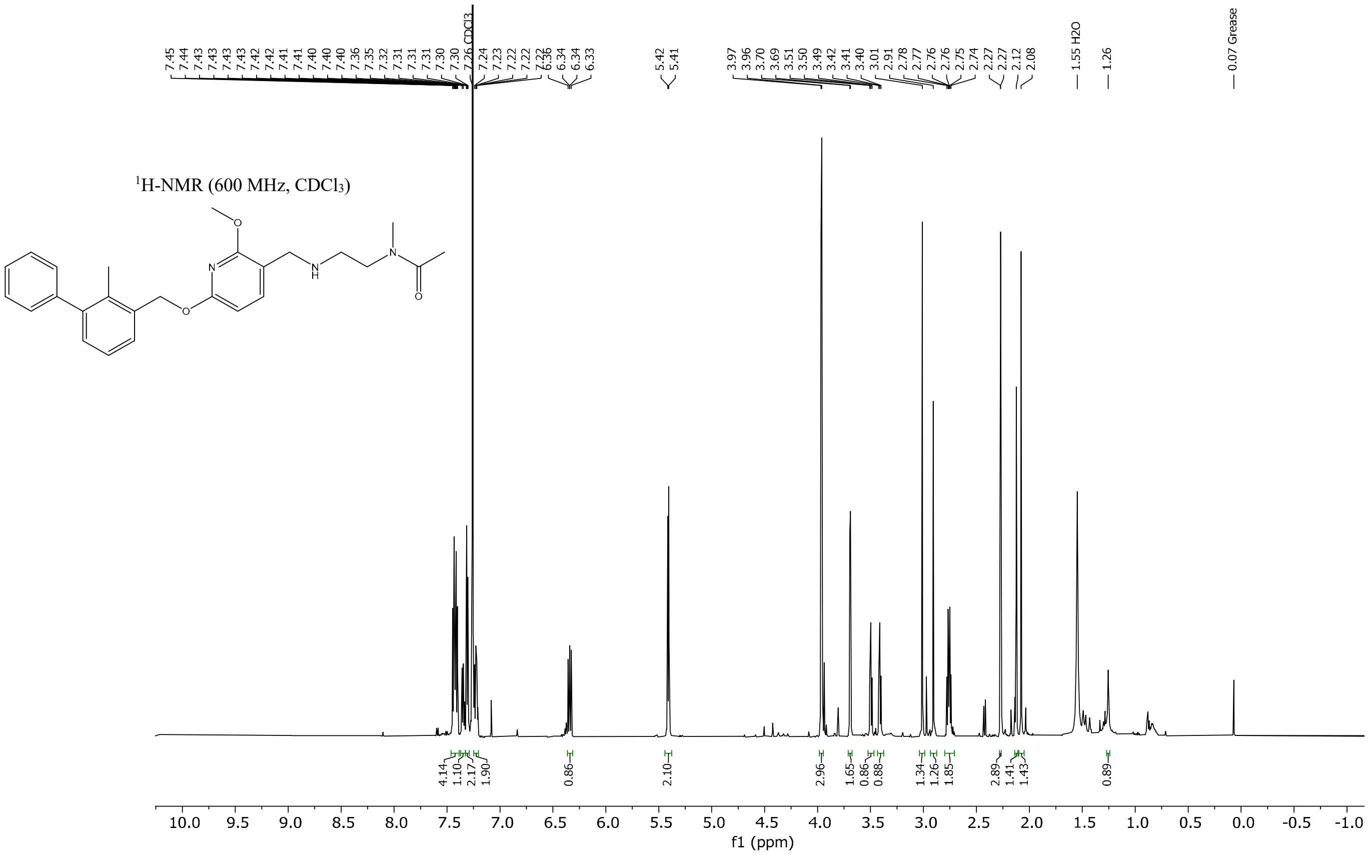

^{1}H-NMR (600 MHz, $CDCl_3$): δ 7.45 – 7.40 (m, 4H), 7.36 – 7.33 (m, 1H), 7.32 – 7.30 (m, 2H), 7.24 – 7.21 (m, 2H), 6.35 and 6.33 (d*, *J* = 7.9 Hz, 1H), 5.42 and 5.41 (s*, 2H), 3.97 and 3.96 (s*, 3H), 3.70 and 3.69 (s*, 2H), 3.50 and 3.41 (t*, *J* = 6.7 Hz, 2H), 3.01 and 2.91 (s, 3H), 2.77 and 2.75 (t*, *J* = 6.7 Hz, 2H), 2.27 (s*, 3H), 2.12 and 2.08 (s*, 3H).

Note: Certain peaks are split due to the presence of amide rotamers and are denoted with an asterisk.

Supplementary Figure S34: *N*-(2-(((2-methoxy-6-((2-methyl-[1,1'-biphenyl]-3-yl)methoxy)pyridin-3-yl)methyl)(methyl)amino)ethyl)-*N*-methylacetamide (**2c**):

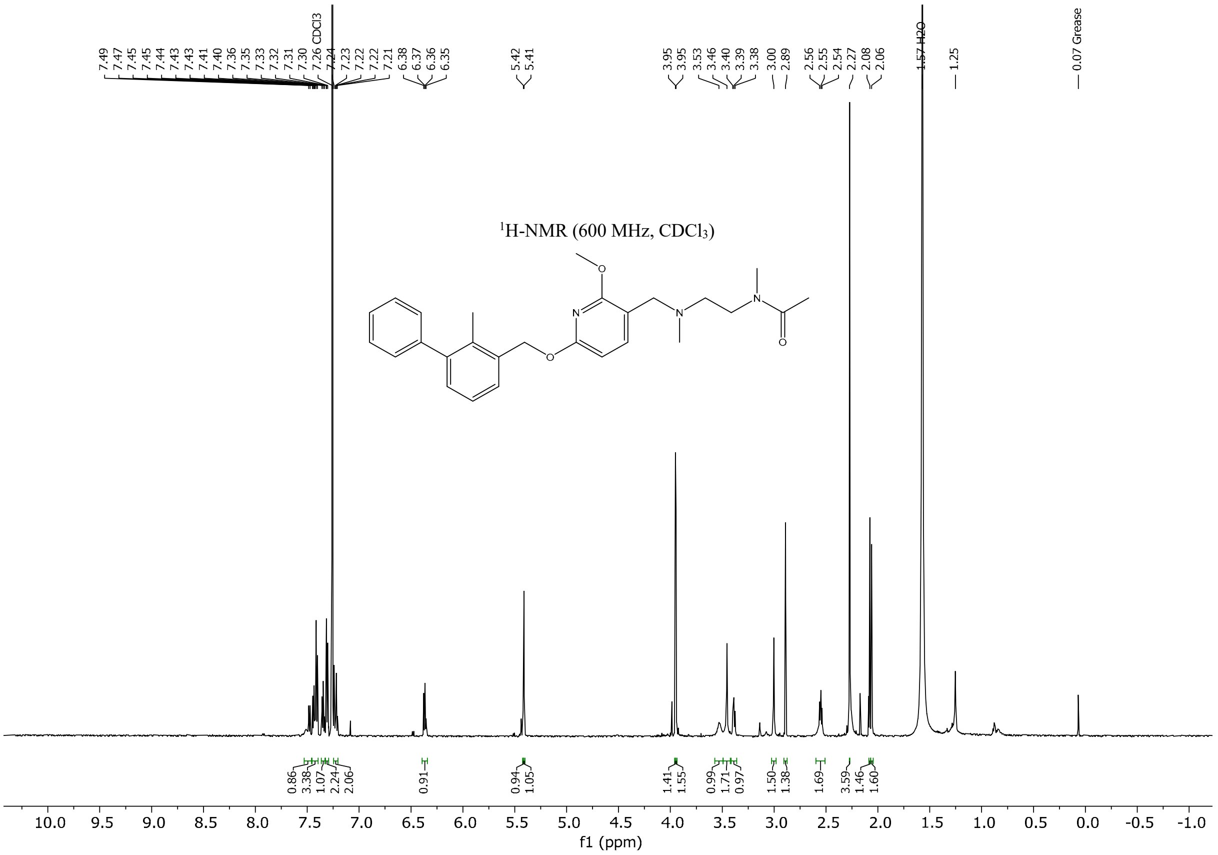

^{1}H-NMR (600 MHz, $CDCl_3$): δ 7.49 – 7.40 (m, 4H), 7.36 – 7.30 (m, 3H), 7.24 – 7.21 (m, 2H), 6.37 and 6.36 (d*, *J* = 7.9 Hz, 1H), 5.42 and 5.41 (s*, 2H), 3.95 and 3.95 (s*, 3H), 3.53 and 3.39 (m and t*, *J* = 7.2 Hz, 2H), 3.46 (s, 2H), 3.00 and 2.89 (s, 3H), 2.55 (t, *J* = 7.2 Hz, 2H), 2.27 (s, 3H), 2.08 and 2.06 (s*, 3H).

Note: Certain peaks are split due to the presence of amide rotamers and are denoted with an asterisk.

Supplementary Figure S35: 2-fluoroethyl (2-acetamidoethyl)((2-methoxy-6-((2-methyl-[1,1'-biphenyl]-3-yl)methoxy)pyridin-3-yl)methyl)carbamate (**2d**):

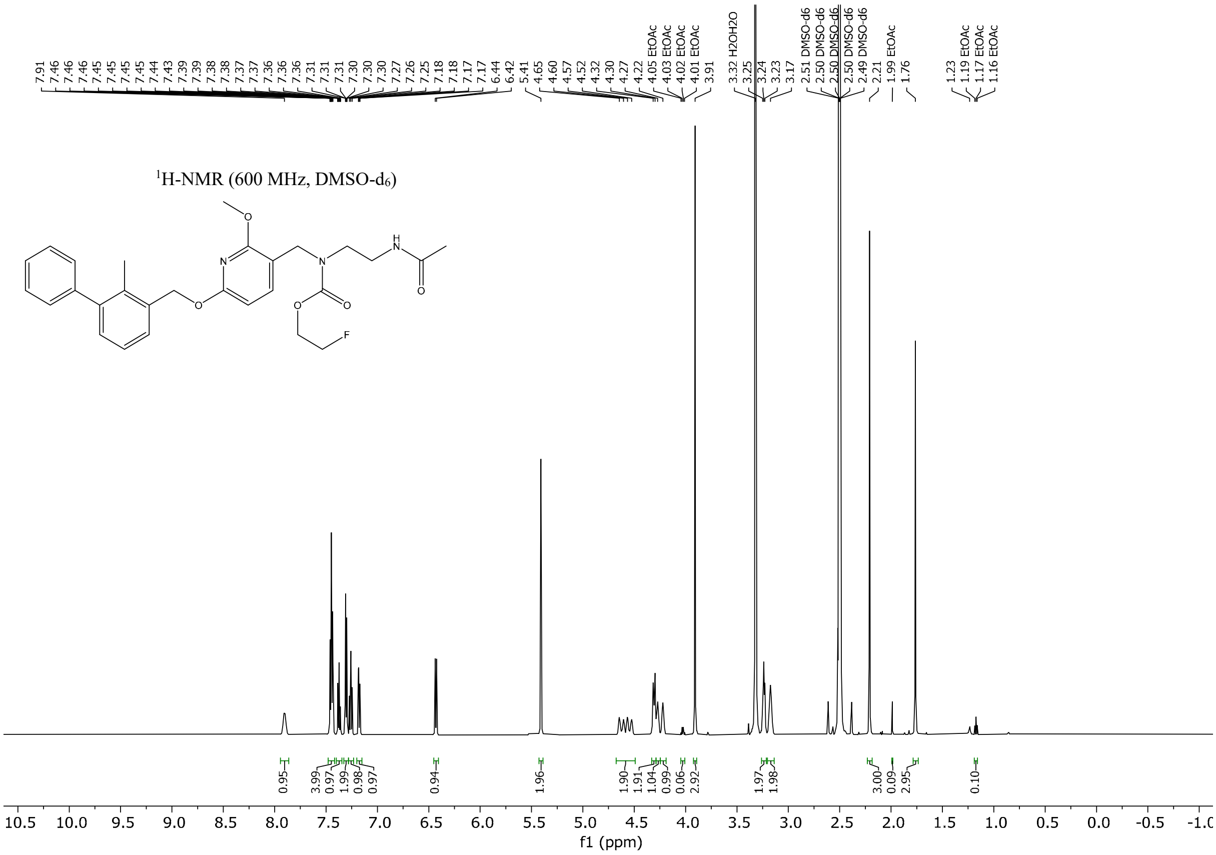

^{1}H-NMR (600 MHz, DMSO-d_6): δ 7.91 (br s, 1H), 7.45 (m, 4H), 7.37 (tt, *J* = 7.5 Hz, *J* = 1.3 Hz, 1H), 7.30 (m, 2H), 7.26 (t, *J* = 7.6 Hz, 1H), 7.18 (dd, *J* = 7.6 Hz, *J* = 1.4 Hz, 1H), 6.43 (d, *J* = 8.0 Hz, 1H), 5.41 (s, 2H), 4.65 – 4.52 (m, *J* = 48 Hz, 2H), 4.31 (d, *J* = 11 Hz, 2H), 4.27 – 4.22 (m, *J* = 30 Hz, 2H), 3.91 (s, 3H), 3.24 (t, *J* = 6.6 Hz, 2H), 3.17 (m, 2H), 2.21 (s, 3H), 1.76 (s, 3H).

Supplementary Figure S36: 2-fluoroethyl (2-acetamidoethyl)((2-methoxy-6-((2-methyl-[1,1'-biphenyl]-3-yl)methoxy)pyridin-3-yl)methyl)carbamate (**2d**):

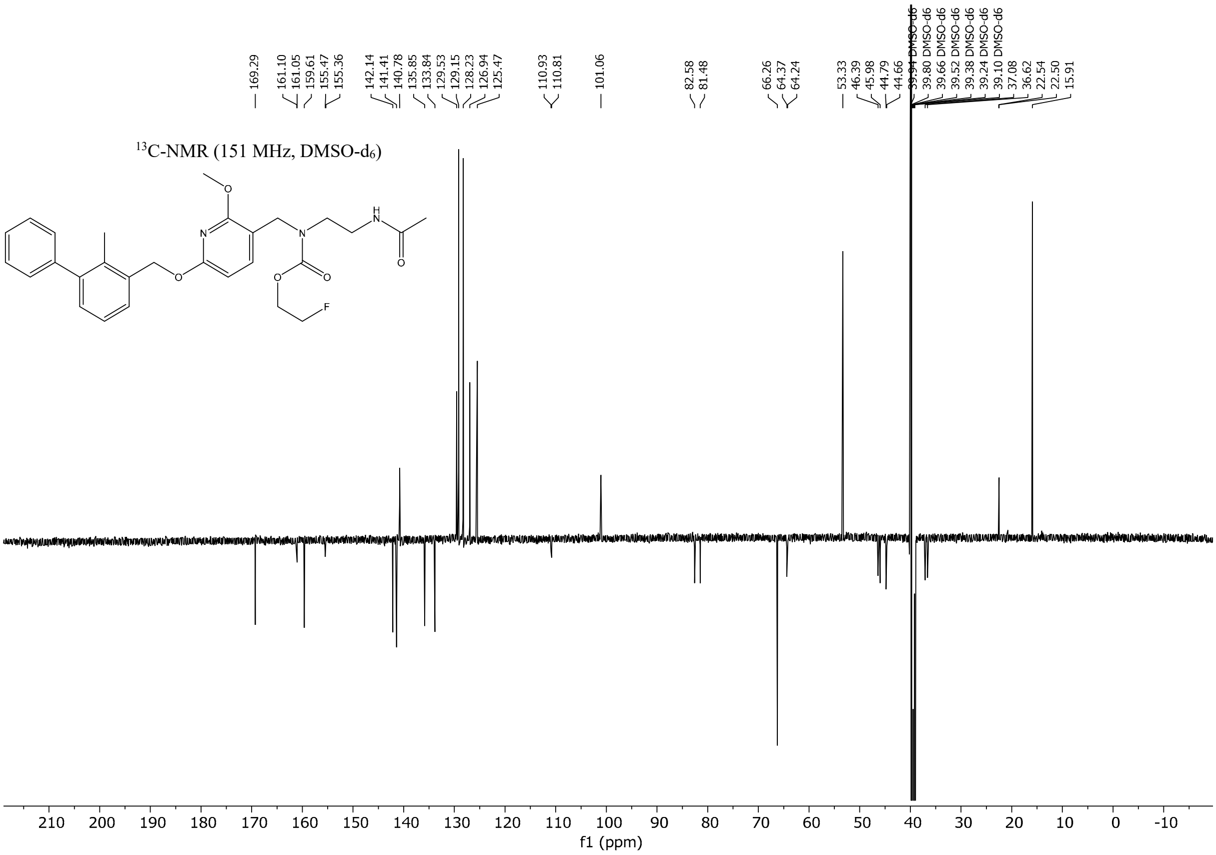

^{13}C-NMR (151 MHz, DMSO-d$_6$): δ 169.29, 161.07 (J = 7 Hz), 159.61, 155.42 (J = 16 Hz), 142.14, 141.41, 140.78, 135.85, 133.84, 129.53, 129.15, 128.23, 126.94, 125.47, 110.87 (J = 18 Hz), 101.06, 82.03 (J = 166 Hz), 66.26, 64.30 (J = 19 Hz), 53.33, 46.19 (J = 61 Hz), 44.72 (J = 20 Hz), 36.85 (J = 69 Hz), 22.52 (J = 7 Hz), 15.91.

Supplementary Figure S37: 2-fluoroethyl (2-acetamidoethyl)((2-methoxy-6-((2-methyl-[1,1'-biphenyl]-3-yl)methoxy)pyridin-3-yl)methyl)carbamate (**2d**):

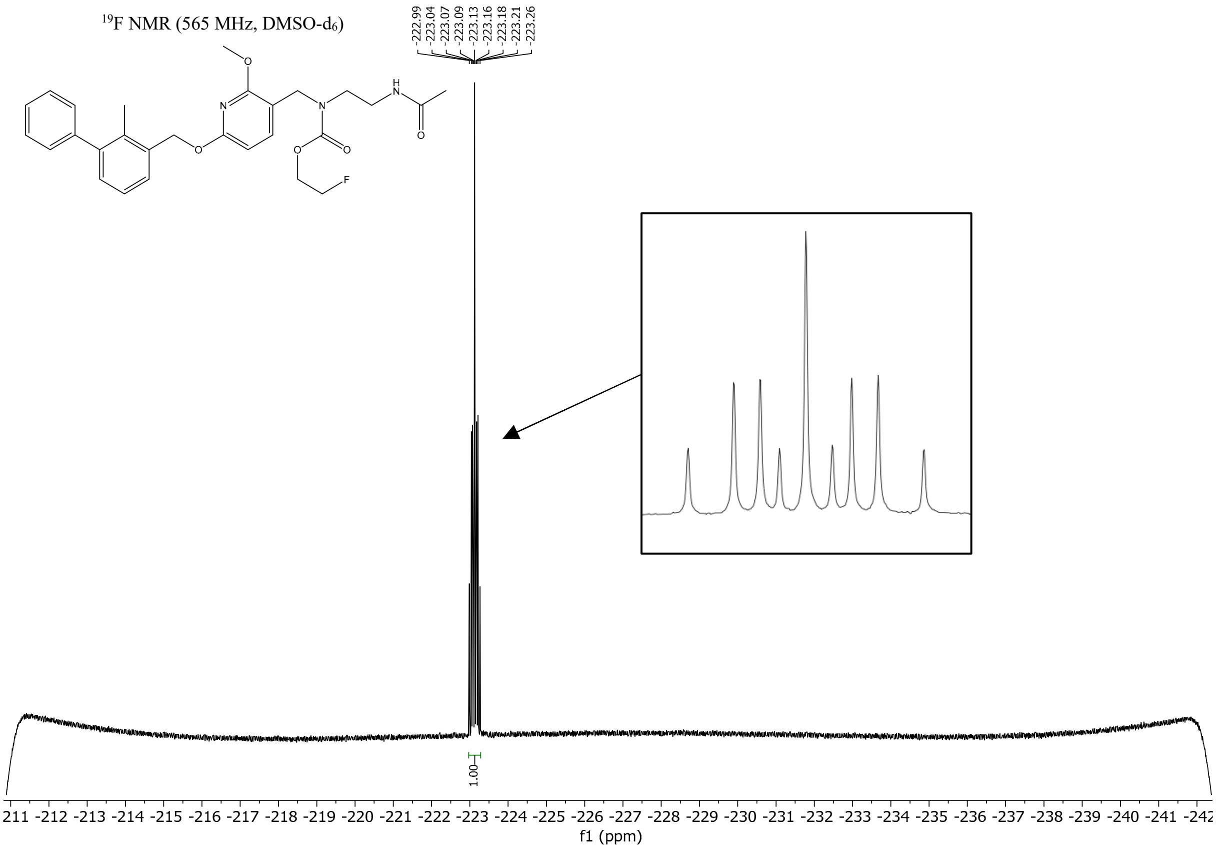

^{19}F-NMR (565 MHz, DMSO-d_6): δ -223.13 (tt, J = 48 Hz, J = 30 Hz, 1F).

Supplementary Figure S38: 2-fluoroethyl (2-acetamidoethyl)((2-methoxy-6-((2-methyl-[1,1'-biphenyl]-3-yl)methoxy)pyridin-3-yl)methyl)carbamate (**2d**):

^1H-^1H COSY NMR (600 MHz, DMSO-d_6)

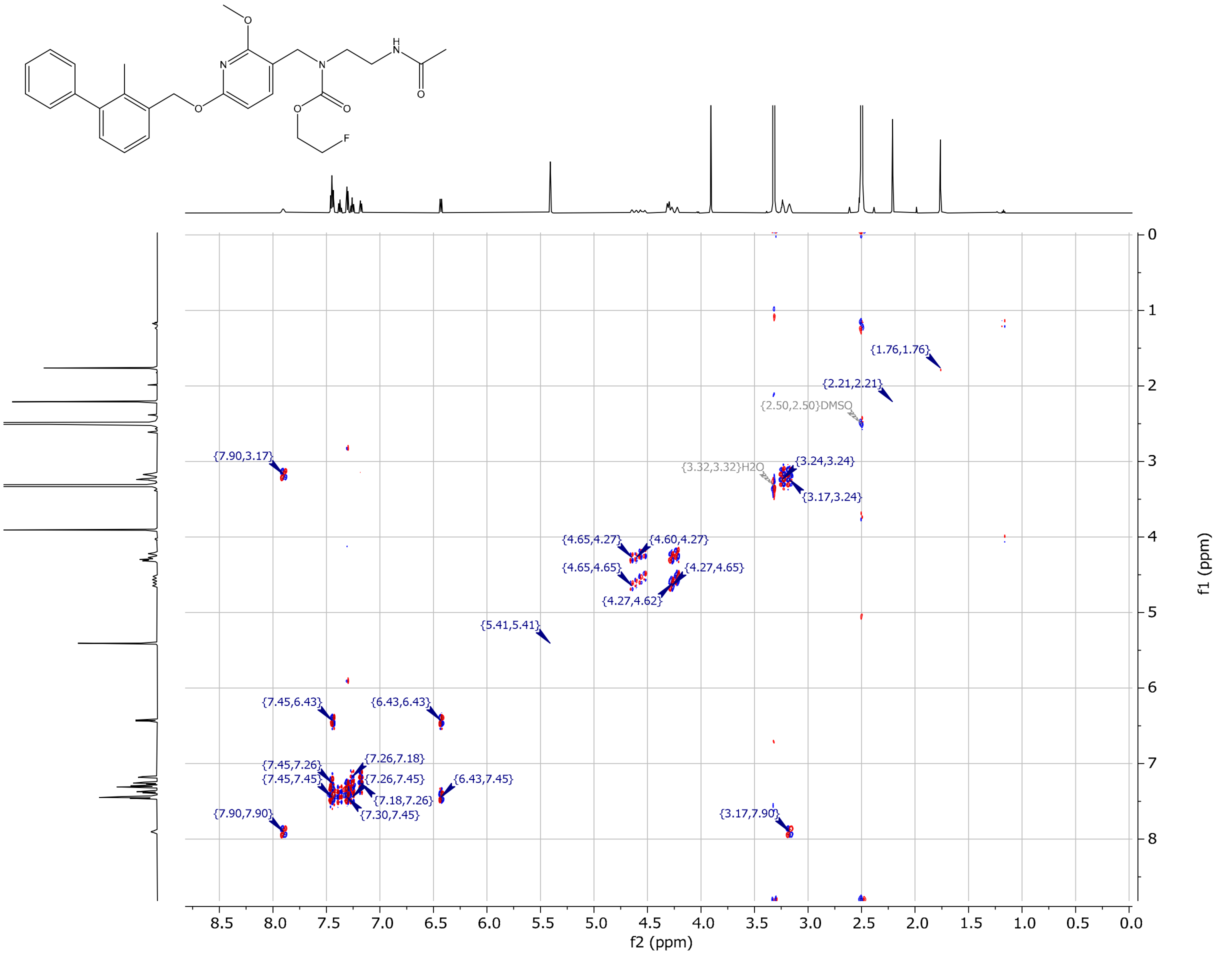

^1H-NMR (600 MHz, DMSO-d_6): δ 7.90, 7.90, 7.45, 7.45, 7.45, 7.45, 7.45, 7.37, 7.37, 7.30, 7.30, 7.26, 7.26, 7.26, 7.18, 7.18, 6.43, 6.43, 5.41, 4.65, 4.65, 4.62, 4.60, 4.57, 4.57, 4.52, 4.52, 4.27, 4.27, 4.27, 4.22, 4.22, 4.22, 3.24, 3.24, 3.17, 3.17, 3.17, 2.21, 1.76

^1H-NMR (600 MHz, DMSO-d_6): δ 7.90, 7.90, 7.45, 7.45, 7.45, 7.45, 7.45, 7.37, 7.37, 7.30, 7.30, 7.26, 7.26, 7.26, 7.18, 7.18, 6.43, 6.43, 5.41, 4.65, 4.65, 4.60, 4.60, 4.57, 4.57, 4.52, 4.52, 4.27, 4.27, 4.27, 4.22, 4.22, 4.22, 3.24, 3.24, 3.17, 3.17, 3.17, 2.21, 1.76.

Supplementary Figure S39: 2-fluoroethyl (2-acetamidoethyl)((2-methoxy-6-((2-methyl-[1,1'-biphenyl]-3-yl)methoxy)pyridin-3-yl)methyl)carbamate (**2d**):

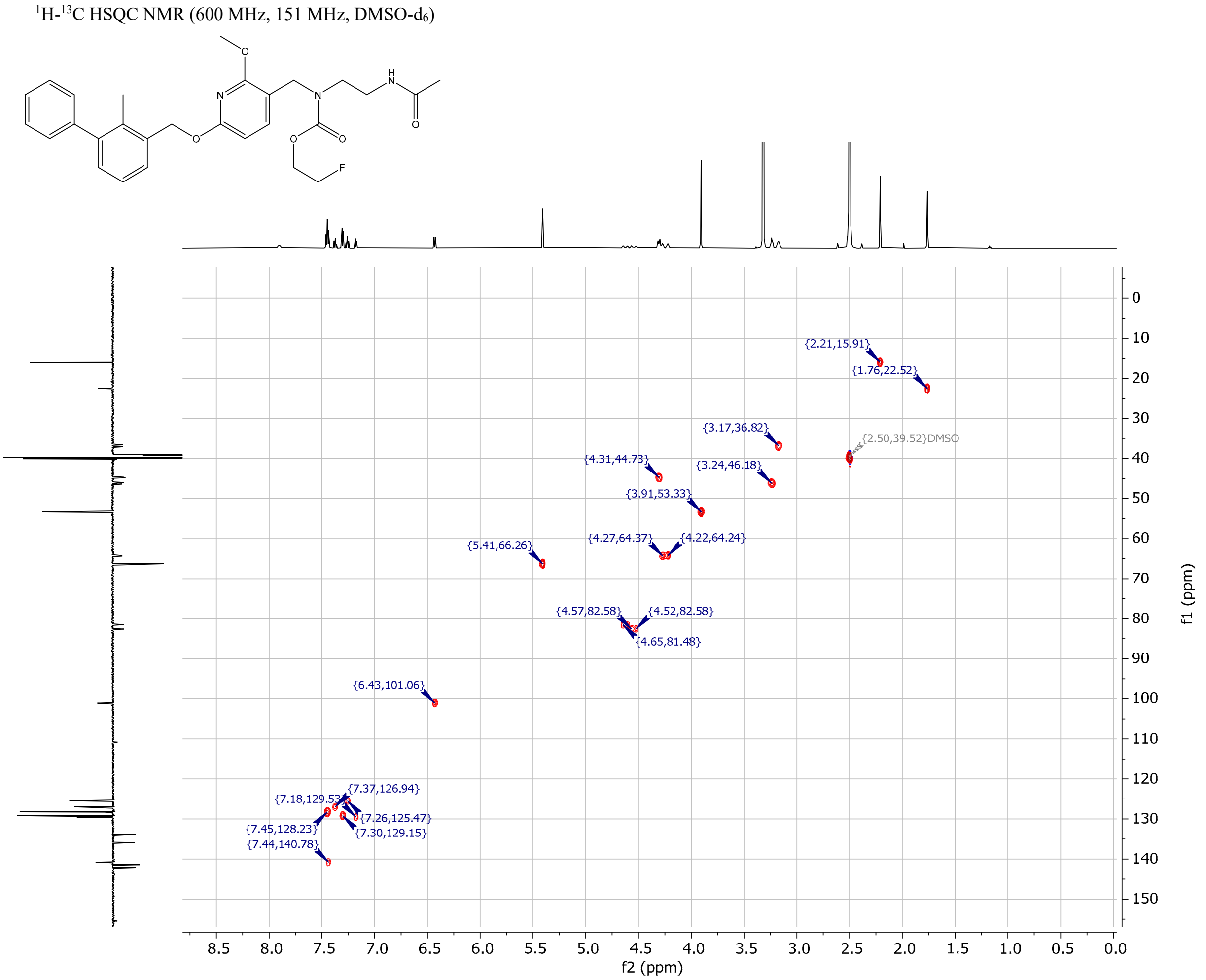

^{13}C-NMR (151 MHz, DMSO-d$_6$): δ 140.78, 129.53, 129.15, 128.23, 126.94, 125.47, 101.06, 82.58, 82.58, 81.48, 81.48, 66.26, 64.37, 64.24, 53.33, 46.18, 44.73, 36.82, 22.52, 15.91.

^{1}H-NMR (600 MHz, DMSO-d$_6$): δ 7.45, 7.44, 7.37, 7.30, 7.26, 7.18, 6.43, 5.41, 4.65, 4.60, 4.57, 4.52, 4.31, 4.27, 4.22, 3.91, 3.24, 3.17, 2.21, 1.76.

Mass spectrometry

Supplementary Figure S40: Methyl (*S*)-1-(2,6-dimethoxy-4-((2-methyl-[1,1'-biphenyl]-3-yl)methoxy)benzyl)piperidine-2-carboxylate (**1a**):

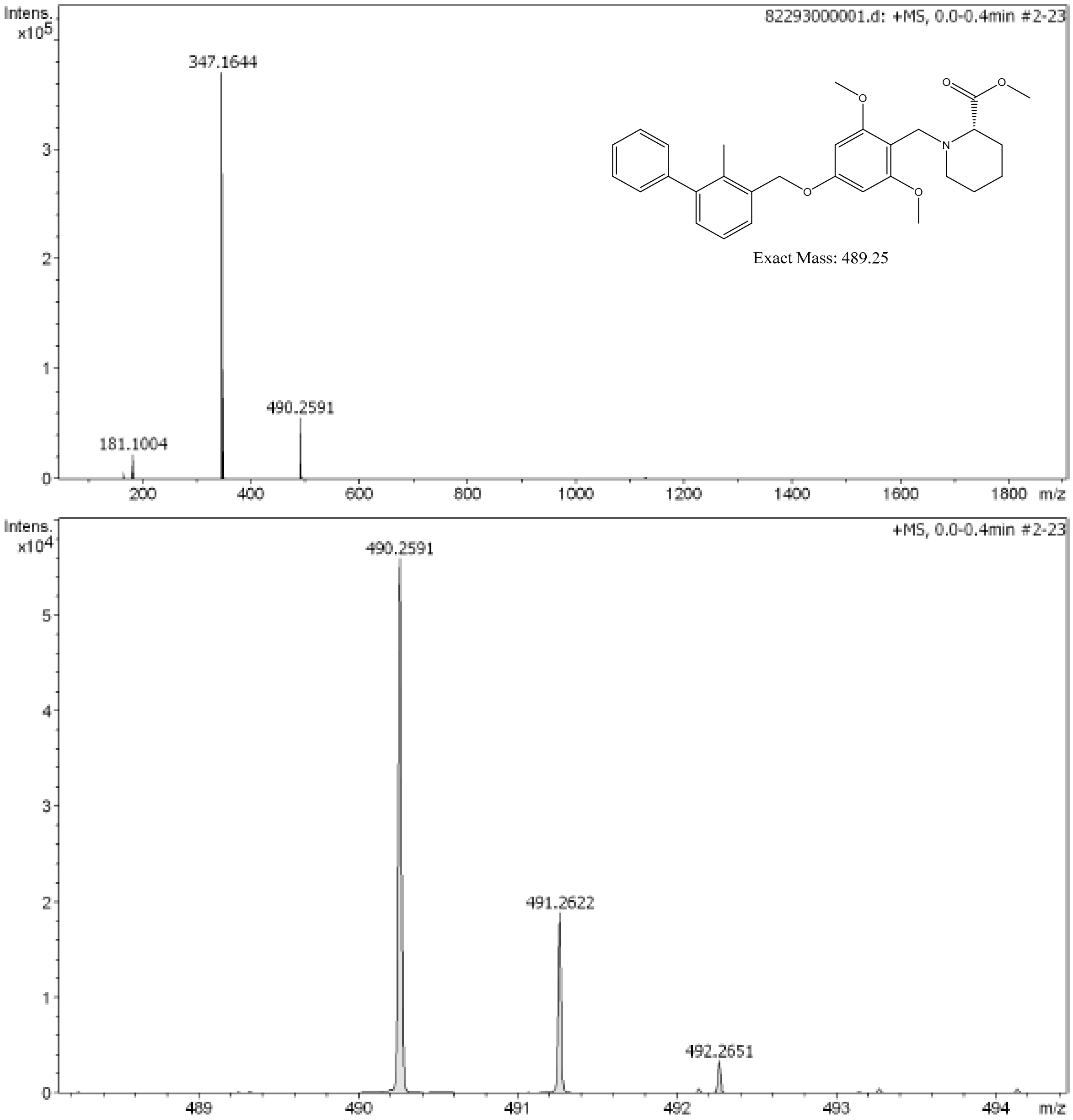

Supplementary Figure S41: 2-Fluoroethyl (*S*)-1-(2,6-dimethoxy-4-((2-methyl-[1,1'-biphenyl]-3-yl)methoxy)benzyl)piperidine-2-carboxylate (**1b**):

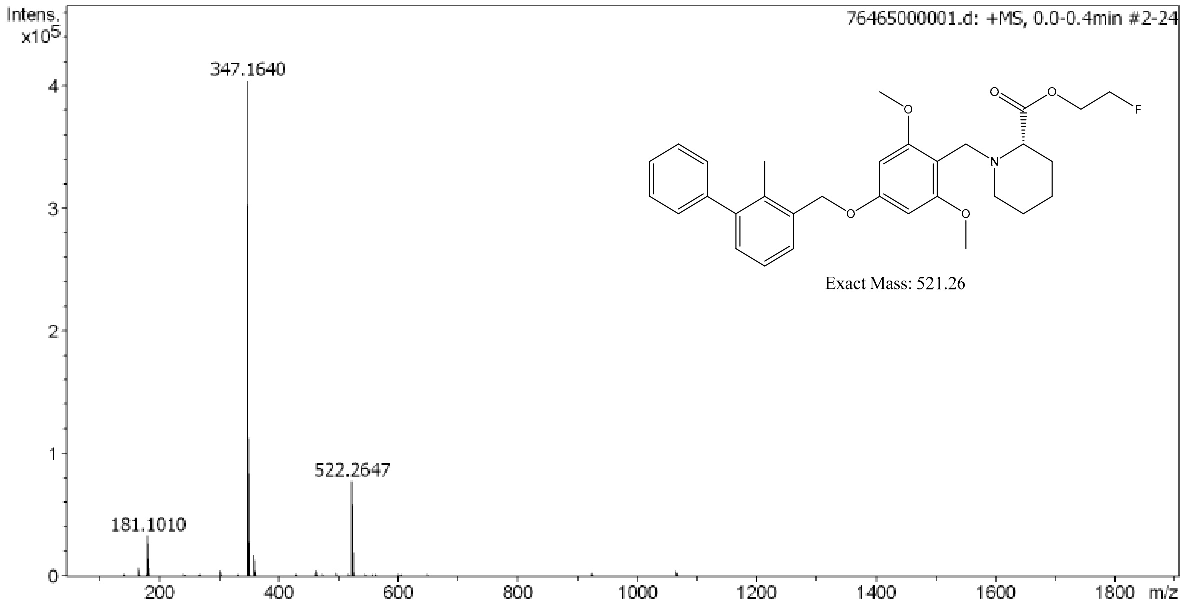

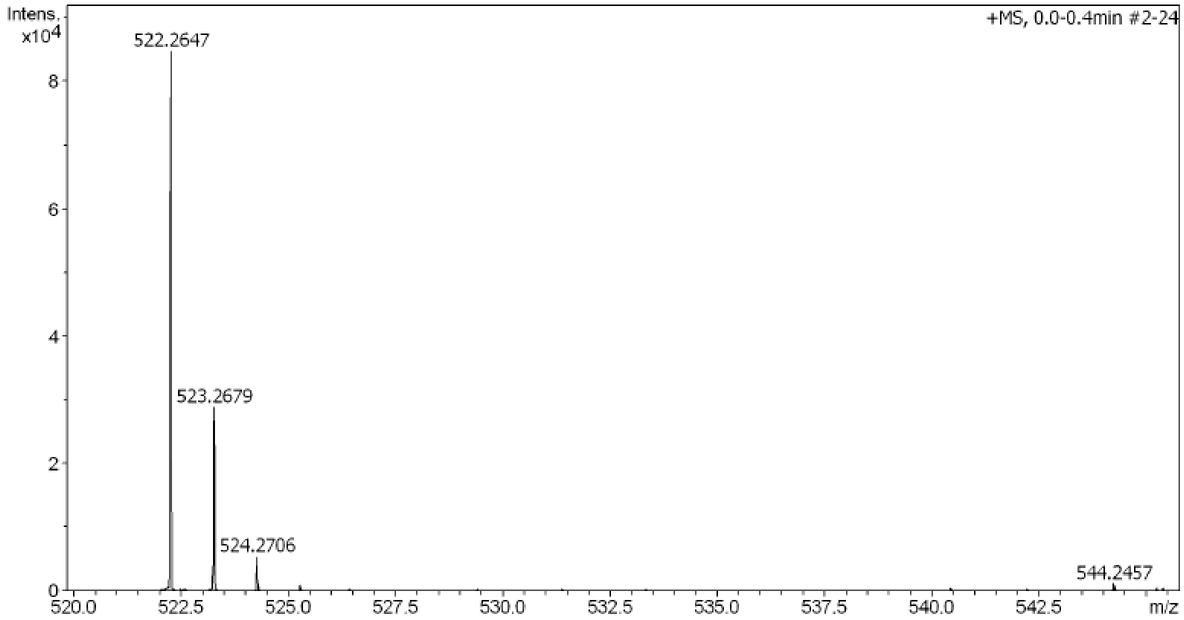

Supplementary Figure S42: *N*-(2-(((2-methoxy-6-((2-methyl-[1,1'-biphenyl]-3-yl)methoxy)pyridin-3-yl)methyl)(methyl)amino)ethyl)acetamide (**2a**):

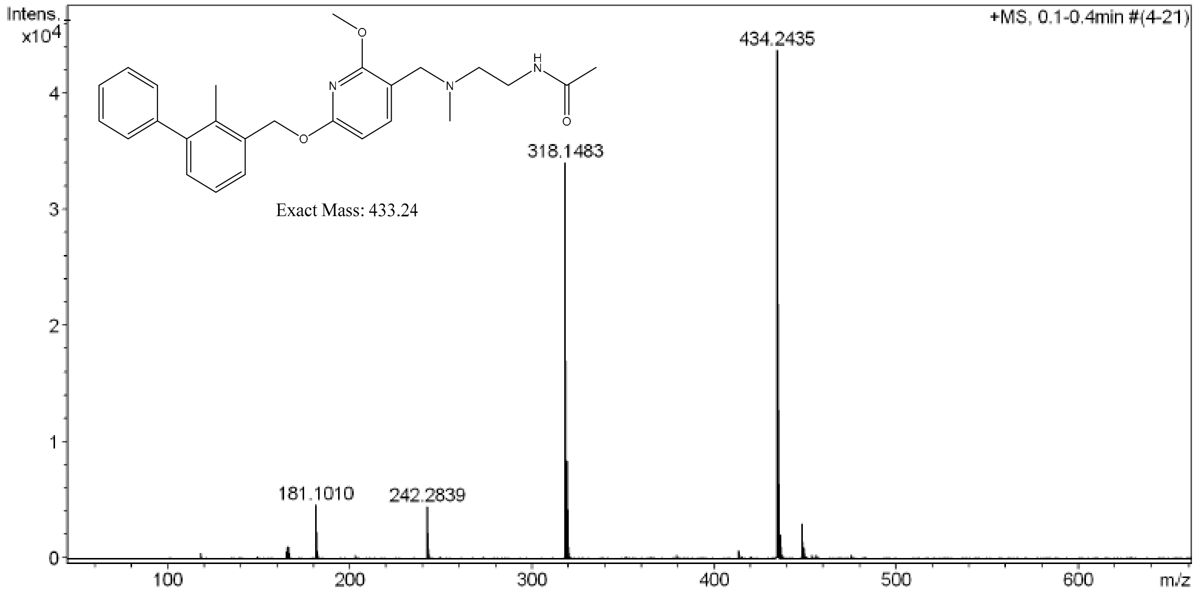

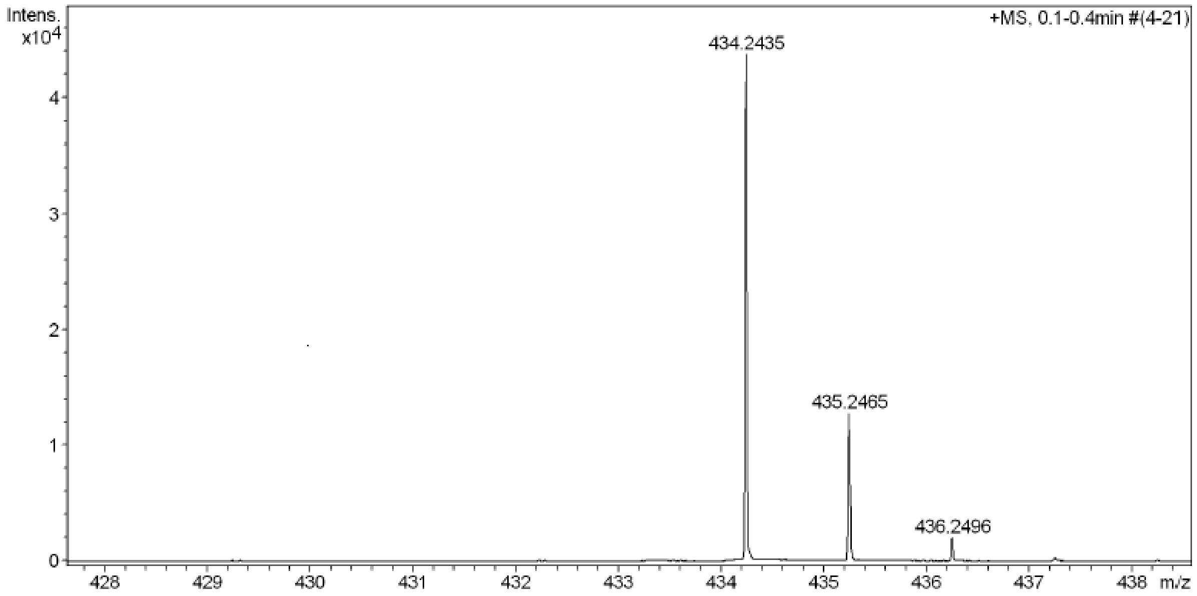

Supplementary Figure S43: *N*-(2-(((2-methoxy-6-((2-methyl-[1,1'-biphenyl]-3-yl)methoxy)pyridin-3-yl)methyl)amino)ethyl)-*N*-methylacetamide (**2b)**:

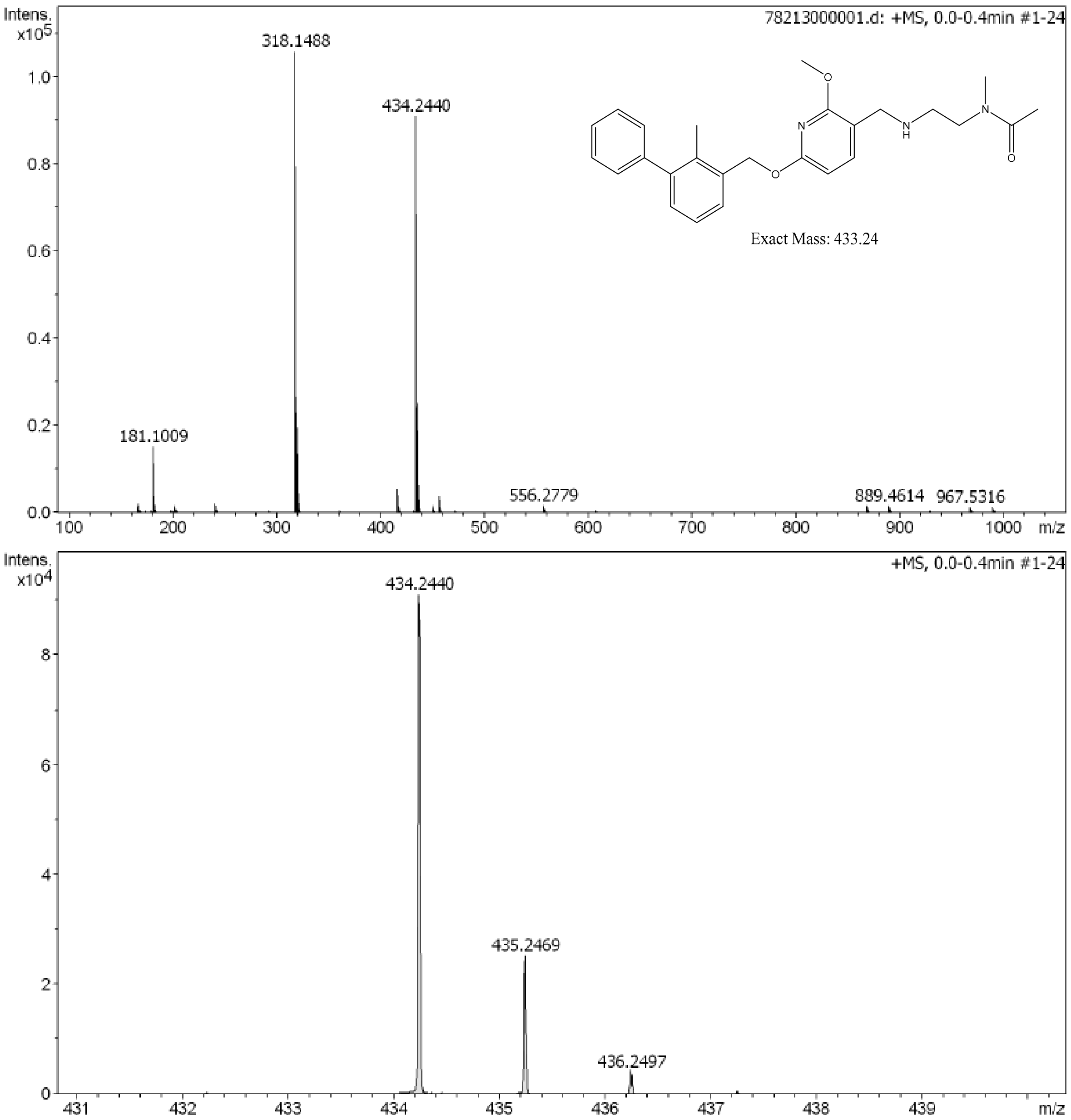

Supplementary Figure S44: *N*-(2-(((2-methoxy-6-((2-methyl-[1,1'-biphenyl]-3-yl)methoxy)pyridin-3-yl)methyl)(methyl)amino)ethyl)-*N*-methylacetamide (**2c**):

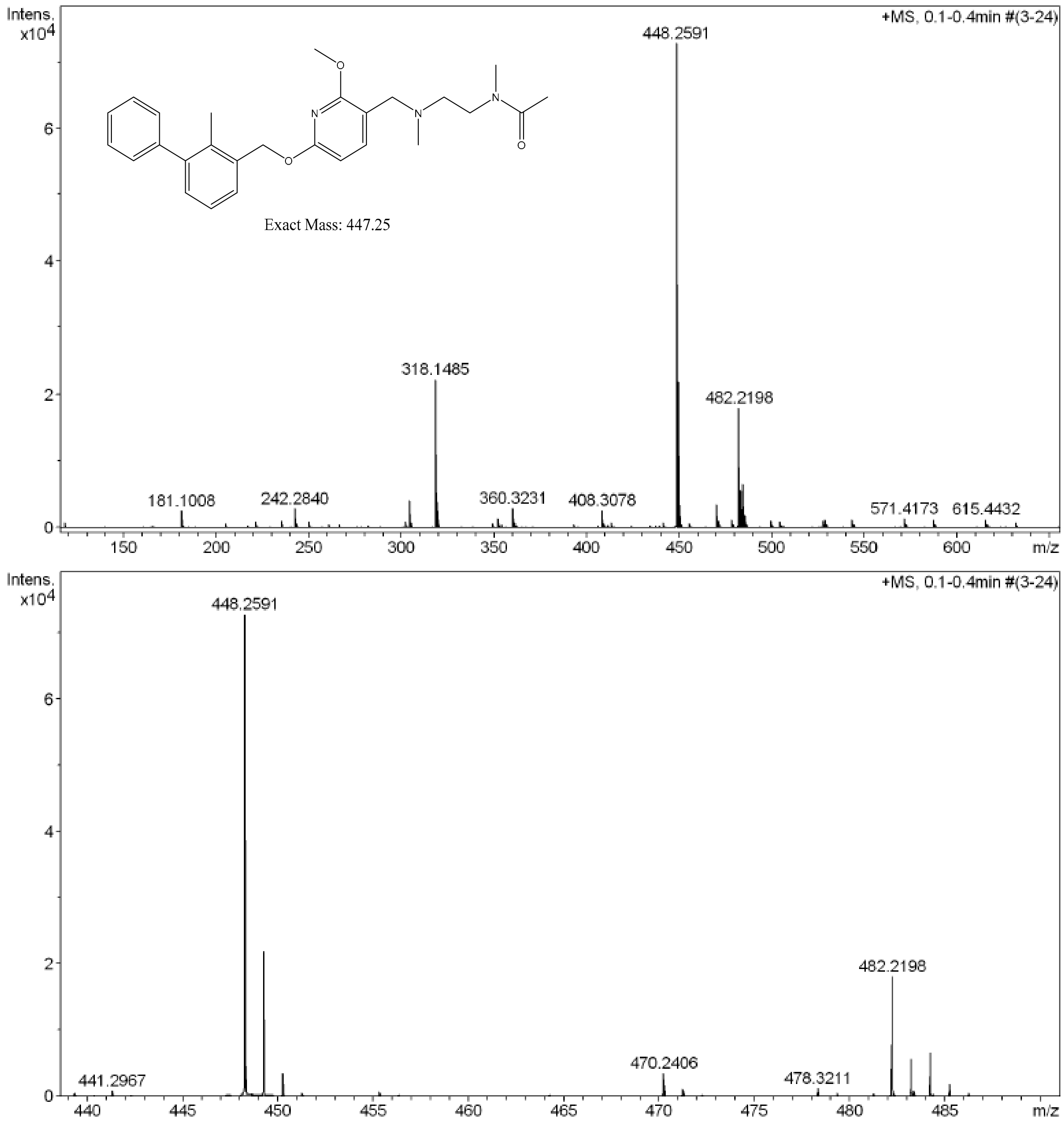

Supplementary Figure S45: 2-fluoroethyl (2-acetamidoethyl)((2-methoxy-6-((2-methyl-[1,1'-biphenyl]-3-yl)methoxy)pyridin-3-yl)methyl)carbamate (**2d**):

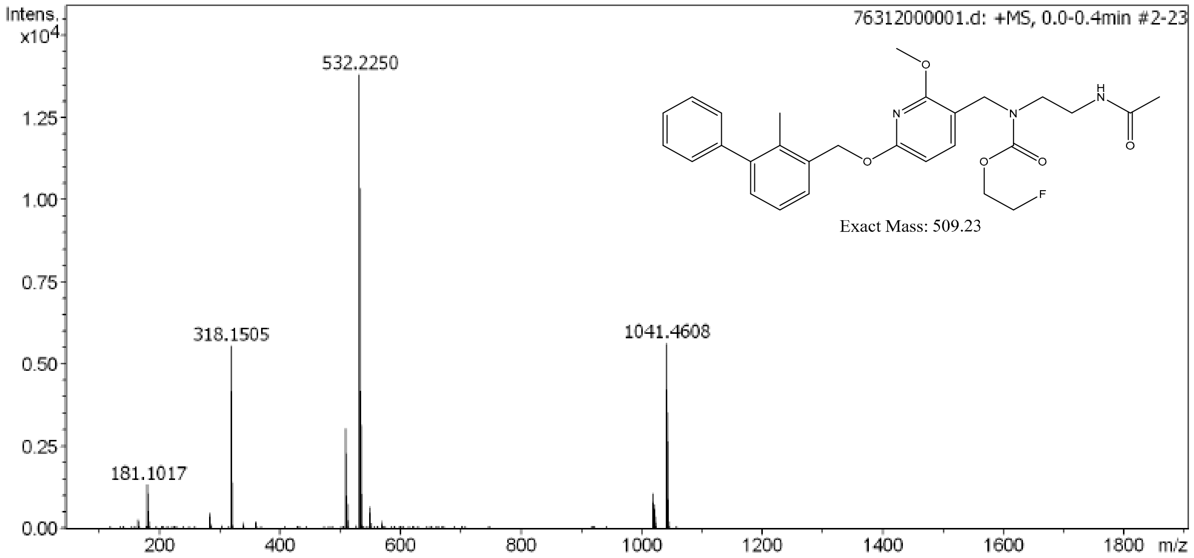

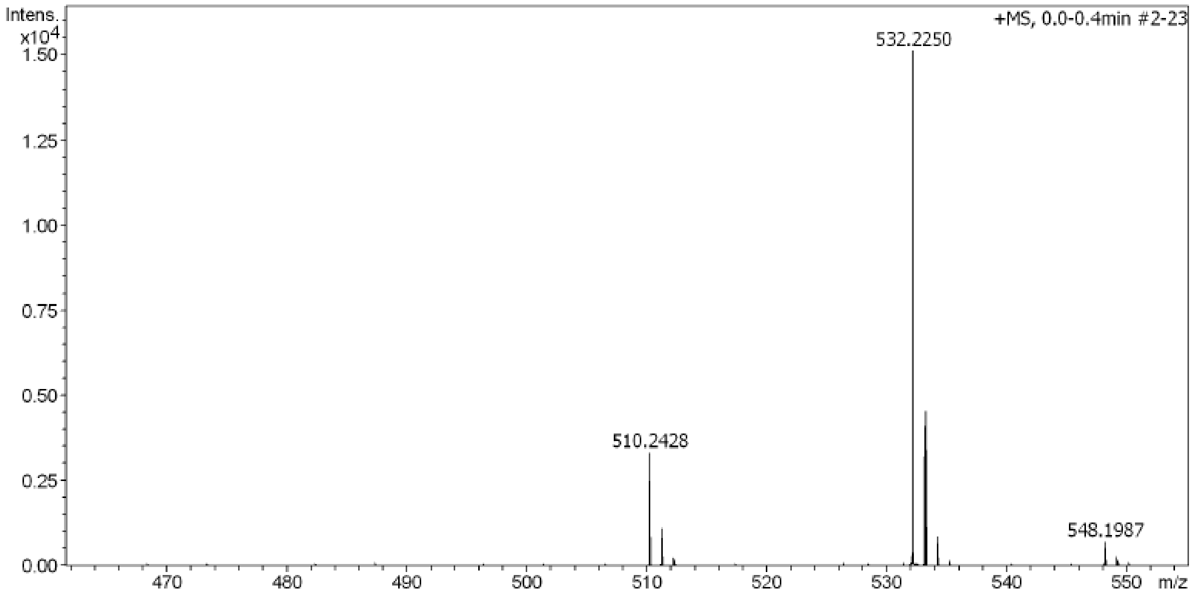

Supplement: Supplementary file 1 [file pharmaceuticals-16-01051-s001.zip › pharmaceuticals-2498599-supplementary.pdf]
